# Metagenomic ene-reductases for the bioreduction of sterically challenging enones†

**DOI:** 10.1039/c9ra06088j

**Published:** 2019-11-11

**Authors:** Dragana Dobrijevic, Laure Benhamou, Abil E. Aliev, Daniel Méndez-Sánchez, Natalie Dawson, Damien Baud, Nadine Tappertzhofen, Thomas S. Moody, Christine A. Orengo, Helen C. Hailes, John M. Ward

**Affiliations:** Department of Biochemical Engineering, University College London Bernard Katz Building London WC1H 6BT UK j.ward@ucl.ac.uk; Department of Chemistry, University College London 20 Gordon Street London WC1H 0AJ UK h.c.hailes@ucl.ac.uk; Structural and Molecular Biology, University College London London WC1E 6BT UK; Almac, Department of Biocatalysis & Isotope Chemistry 20 Seagoe Industrial Estate Craigavon BT63 5QD N. Ireland UK

## Abstract

Ene-reductases (ERs) of the Old Yellow Enzyme family catalyse asymmetric reduction of activated alkenes providing chiral products. They have become an important method in the synthetic chemists' toolbox offering a sustainable alternative to metal-catalysed asymmetric reduction. Development of new biocatalytic alkene reduction routes, however needs easy access to novel biocatalysts. A sequence-based functional metagenomic approach was used to identify novel ERs from a drain metagenome. From the ten putative ER enzymes initially identified, eight exhibited activities towards widely accepted mono-cyclic substrates with several of the ERs giving high reaction yields and stereoselectivities. Two highly performing enzymes that displayed excellent co-solvent tolerance were used for the stereoselective reduction of sterically challenging bicyclic enones where the reactions proceeded in high yields, which is unprecedented to date with wild-type ERs. On a preparative enzymatic scale, reductions of Hajos–Parish, Wieland–Miescher derivatives and a tricyclic ketone proceeded with good to excellent yields.

## Abstract 2

Exceptional organic solvent tolerant ene-reductases mined from a drain metagenome library are highly versatile catalysts for difficult enones.

## Graphical abstract

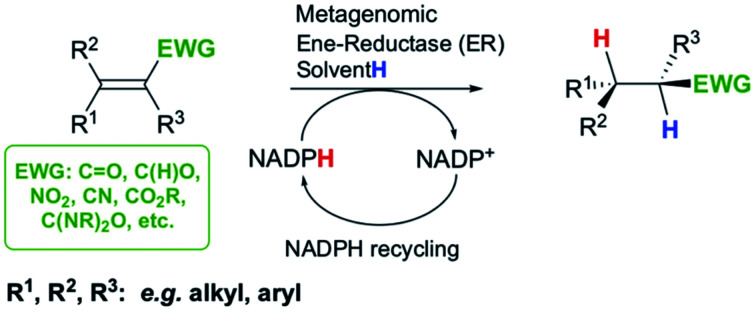

## Introduction

**Fig. 1:**
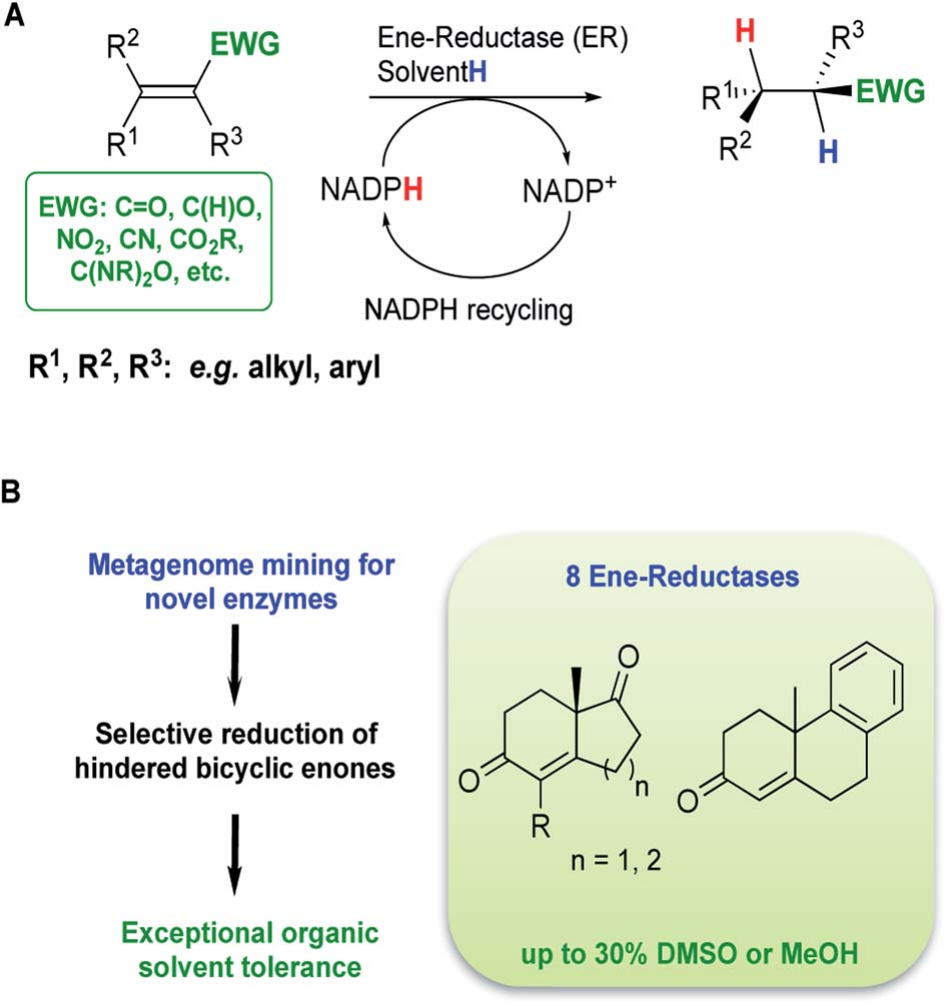
(A) Reduction of activated alkenes catalysed by ERs from the OYE family, (B) overview of this study.

Over the last 30 years, regulations from the FDA and the EMA have become more stringent in relation to product specifications requiring higher optical purities for chiral drug substances.^1^ In addition, the substantial growth in demand for chiral drug substances in new chemical entity pipelines has urged synthetic chemists to discover more efficient and sustainable asymmetric transformations to afford enantiopure molecules.^2^ Biocatalysis is frequently a methodology of choice to achieve selective transformations with minimal environmental impact.^3^ Biocatalytic transformations are generally performed in aqueous-based media with enzymes acting as chiral catalysts. Enzymes exhibit excellent chemo-selectivities, avoiding the need for protecting groups, and possess high asymmetric induction potential. In synthetic methodology, the asymmetric reduction of alkenes allows the possible formation of two chiral centers *via* a formal addition of hydrogen across a double bond.^4^ On an industrial scale, processes typically involve a transition metal complexed to a chiral ligand. Even though these methods achieve good selectivities and yields, catalyst removal is often challenging. Biocatalytic alkene reductions with ene-reductases (ERs) offer an alternative with potential industrial applications.^5–7^ ERs reduce alkenes activated with an electron-withdrawing group and use a co-factor (Fig. 1A). Four different classes of ERs have been explored for applications in biocatalysis, which differ in their stability, reaction mechanism, substrate scope, diastereomer-, and/or enantioselectivity. The most extensively investigated class of ERs is the Old Yellow Enzyme (OYE) family of NAD(P)H dependent flavin containing oxidoreductases (EC 1.6.99.1)^8–10^ that catalyse the reduction of α,β-unsaturated compounds activated with keto, aldehyde and nitro moieties.^11^ Although OYEs have been employed on industrially relevant substrates, such as in the synthesis of high value compounds including pregabalin^12^ and menthol,^13,14^ wider industrial implementation has proved challenging. ERs suffer a number of limitations such as low tolerance to organic co-solvents, low substrate loading tolerance and a narrow substrate range with few reports describing the use of bulky substrates.^5^ Therefore, access to novel, robust and highly active enzymes is vital to develop biocatalytic routes using ERs for larger scale applications.

To date, novel ERs have been identified from bacteria, fungi and plants mainly by conventional microbial screening, genome mining and more recently by a protein data bank search guided by catalytic site structural information.^5,15–17^ Next-generation sequencing technologies and omics approaches have opened up the opportunity to speed up the discovery of useful biocatalysts by providing access to unexplored environmental microbial communities.^18^

In a previous study, we have reported a sequence-based functional metagenomics approach to identify novel biocatalysts.^19,20^ Microbial communities from the human oral cavity and more recently from a domestic drainpipe were investigated.^19–21^ The drain proved to be an interesting niche providing a highly valuable dataset for bioprospecting and led to the identification of native enzymes with notable characteristics, such as organic solvent stability often only observed in highly engineered enzymes.^21^ In the present study, *in silico* searches of the drain metagenome led to the identification of eight novel and active ERs from the OYE family (Fig. 1A) of which two ERs displayed high activity with bi- and tri-cyclic enones (Fig. 1B), on a preparative scale, to generate chiral synthons in good to excellent yields and stereoselectivities.

## Results and discussion

### Drain metagenome and the identification of ERs

Metagenomic DNA was isolated from a sample collected from a domestic shower drainpipe and sequenced by the Illumina MiSeq platform. *De novo* assembly of the drain metagenome resulted in 69 962 contigs with sizes ranging from 500 base pairs (bp) to 67 494 bp and a mean length of 1500 bp. Analysis revealed that 106 954 open reading frames (ORFs) were predicted with 39 915 genes being longer than 600 bp (see ESI†). Approximately 50% of the predicted ORFs had functional domains that could be annotated by the Pfam Protein Family database.^22^ To discover novel metagenomic ERs, predicted protein sequences assigned to the Pfam entry PF00724 were analyzed. According to Pfam, enzymes in this family are NADH flavin oxidoreductase/NADH oxidase that contain a TIM-barrel fold and commonly use FMN/FAD as a cofactor.^22^ Furthermore, we found that all 63 published and biochemically characterized prokaryotic ERs from the Old Yellow Enzyme family belonged to this entry.^9^ In the drain metagenome, from a total of 38 predicted proteins assigned to PF00724, 14 were encoded by full-length genes and 24 by truncated genes. When sequencing reads were assembled into contigs, some of the predicted ORFs extended beyond the boundaries of assembled contigs resulting in N- or C-terminally truncated genes. Although PCR amplification of nearly full-length genes is possible by designing the primer sets based on homologue sequence regions available in public databases, it was decided to focus on the analysis of the full-length genes and their corresponding proteins. Multiple sequence alignments of 14 full-length putative ERs with selected characterized members of the OYE protein family showed that active site residues^8^ were conserved in 10 of the metagenomic proteins (Fig. S1†). These ten putative ERs were taken forward as candidates for cloning and functional characterization. The other four proteins were identified as possible oxygen-sensitive enoate-reductases (EC 1.3.1.31) based on sequence analysis and were not investigated further in this work.

A total of nine out of ten genes encoding putative ERs were successfully amplified directly from the drain metagenomic DNA, cloned with a C-terminal His6 tag and named with plasmid numbers: pQR1439, pQR1440, pQR1442, pQR1443, pQR1445, pQR1446, pQR1907, pQR1908, pQR1909. The nine novel ERs shared sequence identity of between 76% and 100% to protein sequences in the NCBI database (Table S1†). The closest homologues of the metagenomic ERs belonged to different bacterial species, with all coming from *Proteobacteria* phylum, the predominant phylum in the drain metagenome.

Multiple sequence alignments of the nine ERs showed a sequence identity ranging from 22% to 54%. Based on differences in conserved sequence residues the majority of the ERs were assigned to subclasses of OYEs previously described as “classical” and “thermophilic-like” (Fig. S1†).^8^ ERs pQR1439, pQR1443 and pQR1446, however showed differences in the conservation of distinct residues. Phylogenetic analysis (Fig. S1†) further indicated that ERs pQR1907, pQR1908, pQR1445, and pQR1440 belonged to the “classical” group together with OYEs from *Saccharomyces pastorianus* and *Saccharomyces cerevisiae*, NCR from *Zymomonas mobilis*, and pentaerythritol tetranitrate reductase (PETNR) from *Enterobacter cloacae*, while pQR1909 and pQR1442 belonged to ‘thermophilic-like’ ERs populated with mesophilic (YqjM from *Bacillus subtilis*) as well as thermophilic (TOYE from *Thermoanaerobacter pseudoethanolicus*) enzymes.^8,9^ Three remaining ERs clustered apart from these two OYE groups, expanding further newly proposed subclasses in the OYE family phylogenetic tree.^9,39^ pQR1443 and pQR1446 clustered together with Chr-OYE1 from *Chryseobacterium* sp. while pQR1439 clustered close to Nox from *Rhodococcus erythropolis* and YqiG from *Bacillus subtilis* (Fig. S1†).^39^ All the metagenomic ERs were heterologously expressed in *E. coli* BL21 (DE3). The ER NCR has been well studied and successfully used with a range of linear and monocyclic substrates and was therefore used as a reference enzyme.^23^

### Initial screening of metagenomic ERs

To screen the panel of nine metagenomic ERs, purified enzymes were initially used (Fig. S2†). A qualitative spectrophotometric assay with substrates 1 and 2 that are readily accepted by the OYEs,^23^ indicated NADPH-dependent reductase activity for seven of the enzymes. No or low activity was observed for the enzymes expressed from pQR1439 and pQR1443 respectively (Table S2†). To further assess the ERs, activity was quantified using GC-analysis with substrates 1 and 2 to give 3 and 4 respectively, NADPH and glucose-6-phosphate dehydrogenase (G6PDH) to recycle the co-factor *in situ* and glucose-6-phosphate sodium salt (G6PNa) as co-substrate. Seven metagenomic ERs gave high yields (by GC analysis) with both substrates (Table 1), with higher activities towards compound 2 for all enzymes.

**Table 1:** Bioreduction of activated ketones 1 and 2^a^

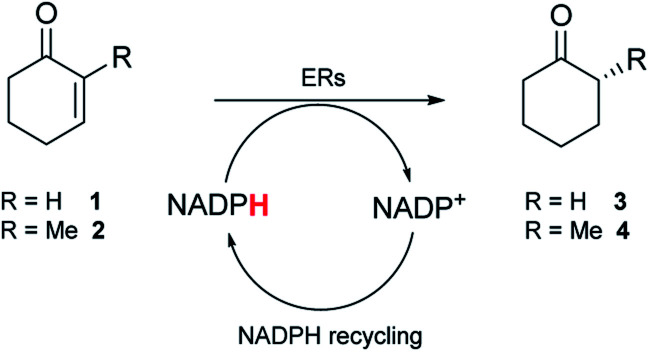			
ERs	3	4	
	Yield [%]	Yield [%]	ee [%]
pQR1907	83	>99	90 (*R*)
pQR1908	76	90	99 (*R*)
pQR1909	80	>99	92 (*R*)
pQR1439	0	0	—
pQR1440	70	87	92 (*R*)
pQR1442	72	86	90 (*R*)
pQR1443	29	39	83 (*R*)
pQR1445	84	>99	99 (*R*)
pQR1446	82	98	83 (*R*)
NCR	65	69	99 (*R*)

a Substrate (10 mM), purified ER (0.2–0.9 mg mL^−1^), NADP^+^ (2.8 mM), G6PDH (20 U), G6PNa (100 mM), in Tris–HCl (50 mM) and DMSO (10%) at pH 7.4, 30 °C, 20 h, 300 rpm. Reactions were performed in triplicate. Yields and ees were determined by GC analysis. 1 and 2 remained unchanged in control reactions with no enzyme present.

As observed in the spectrophotometric assay, the ER expressed from pQR1443 showed low activity, while no activity was observed with the ER from pQR1439; these two enzymes were not used in further experiments. For the active ERs, products were obtained in moderate to high stereoselectivities (83–99% ee) for the *R* enantiomer (Table 1). With NCR, yields and the selectivity obtained for the reduction of 1 and 2 were in agreement with reported values.^23^

Next, the industrially important substrate carvone 5 was tested. Reduction products of 5, (2*R*,5*S*)- and (2*R*,5*R*)-dihydrocarvones give access to chiral building-blocks for the synthesis of natural products (*e.g.* striatenic acid, pechueloic acid), antimalarial drugs, fragrances and bio-renewable polyesters.^24^ The bioreduction of carvone with ERs has been reported, with both carvone enantiomers converted in high yield and selectivity.^6,25,26^ Initial assays using GC analysis and the previously optimised reaction conditions with a reaction time of 20 h resulted in low yields (∼20–30%) of dihydrocarvones 6 and 7 for selected ERs (Fig. S3†). No background reaction was observed in the absence of enzyme. Reducing the reaction time to 3.5 h increased the yields to ∼40–60% (Fig. S4†). For the reduction of *R*-5, lower yields were probably due to previously reported solubility issues.^27^ The formation of 6 was then optimised further with the enzymes expressed by pQR1907 and pQR1445 as they were readily expressed and gave rise to high yields in the reduction of substrates 1, 2 and 5. Interestingly, *S*-5 was reduced to 6 in 1 h (Fig. S5†) with ERs from pQR1907 and pQR1445 in 82% and 81% yields, respectively, with complete selectivity for the (2*R*,5*S*)-isomer (Table 2). Under these reaction conditions turnover frequencies (TOFs) were high; pQR1907 achieved a high TOF of 722 h^−1^ while pQR1445 reached 590 h^−1^ molecules per hour, better than many reported ERs and close to the best reported TOF for (*S*)-carvone (≈1000 h^−1^), by LacER.^37,38^ The bioreduction of *S*-5 with NCR provided the same selectivity in 78% yield and with a TOF of 645 h^−1^ in accordance with the literature.^38^ With the ER from pQR1907 and NCR, isomer *R*-5 was reduced in 66% and 63% respectively, giving exclusively (2*R*,5*R*)-7.

**Table 2:** Reduction of carvones using the ERs pQR1907, pQR1445 and NCR^a^

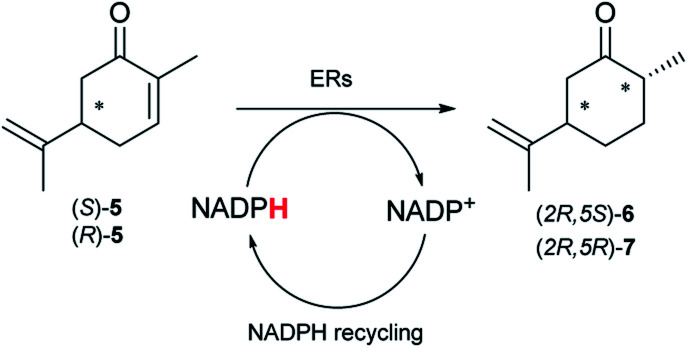		
ER	(2*R*,5*S*)-6	(2*R*,5*R*)-7
	Yield [%]	Yield [%]
pQR1907	82	66
pQR1445	81	n.d.
NCR	78	63

a Substrate (10 mM), purified ER (0.7–0.8 mg mL^−1^), NADP^+^ (2.8 mM), G6PDH (20 U), G6PNa (100 mM), in Tris–HCl (50 mM) and DMSO (10%) at pH 7.4, 30 °C, 1 h, 300 rpm. Reactions were performed in triplicate. Yields and ees were determined by GC analysis; n.d. not determined.

### Solvent tolerance of the enzymes from pQR1907 and pQR1445

Due to the high selectivity and yields towards the substrates used, metagenomic ERs from pQR1907 and pQR1445 were co-expressed with the cofactor-recycling enzyme G6PDH from *Saccharomyces cerevisiae* in *E. coli* BL21 (DE3) and prepared as clarified cell lysates. These co-expressed lysates were reacted with *S*-5, and 6 was obtained in 95% yield (by GC analysis) with the enzyme from pQR1907 (d.r. = 95 : 5) and 96% yield (by GC analysis) with the enzyme from pQR1445 (d.r. = 97 : 3) (Fig. S7†).

**Fig. 2:**
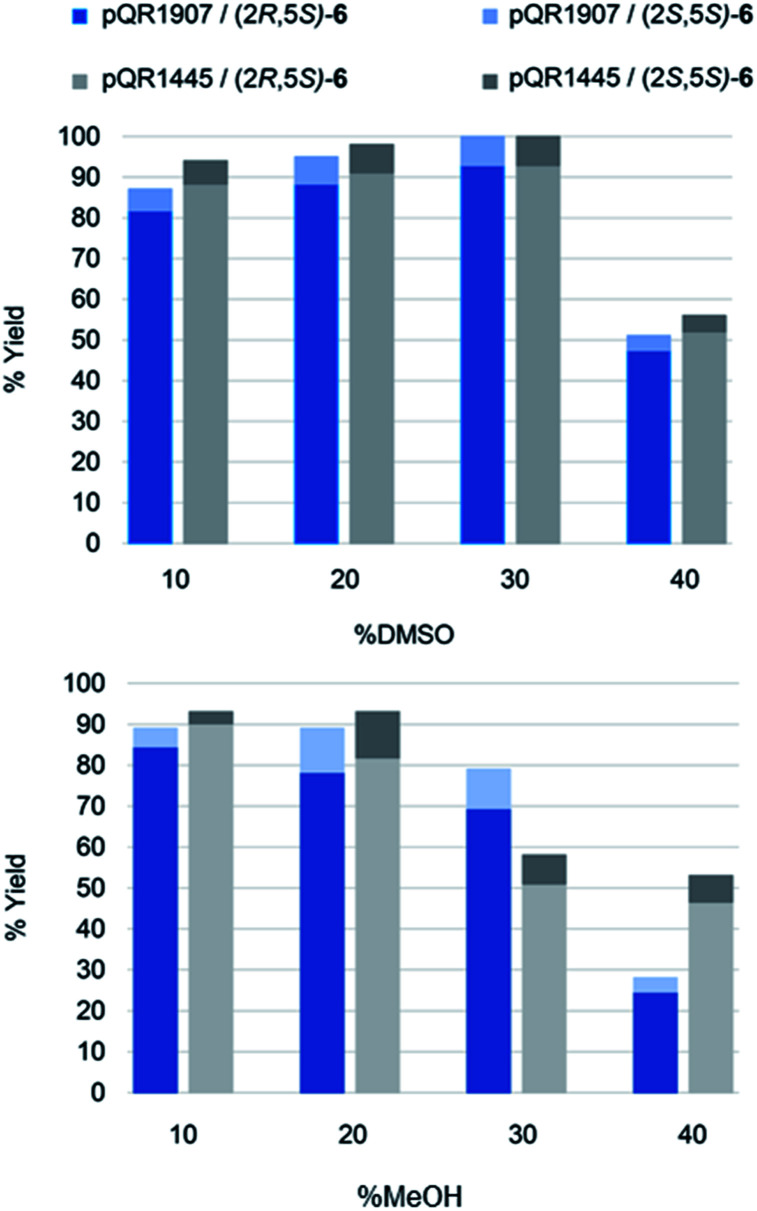
Effect of co-solvent on the reduction of *S*-5. Substrate (10 mM), co-expressed ER/G6PDH (1 mg mL^−1^), NADP^+^ (2.8 mM), G6PNa (100 mM), in Tris HCl (50 mM) and co-solvent (%) at pH = 7.4, 30 °C, 1 h, 300 rpm. Reactions were performed in triplicate. Yields and the diastereoselective ratio (d.r.) of 6 were determined by GC-analysis. Standard deviations were below 5%.

Activities towards *S*-5 were then investigated in the presence of different concentrations of the water-miscible co-solvents, DMSO and methanol (10–50% v/v) (Fig. 2). Both enzymes were tolerant of up to 30% DMSO, after which a significant drop in yields was observed. Methanol was also well tolerated with only a slight loss of activity at a 30% level. Additionally, the reaction selectivity decreased from d.r. = 95 : 5 to d.r. = 88 : 12 for the enzyme from pQR1907 and from d.r. = 97 : 3 to d.r. = 88 : 12 for the enzyme from pQR1445 when using *S*-5 and higher amounts of methanol, but little change in the selectivity was observed when using up to 40% of DMSO.

This high-co-solvent tolerance of both the ERs and the cofactor recycling enzyme is notable and highlights their potential utility. The use of DMSO in up to 50% v/v has been used with ERs^40^ but much lower conversions (and ees) were observed at greater than 20% v/v DMSO. No methanol was used in that study. In addition, that report added GDH to recycle the cofactor rather than co-expression of a GDH. Another study^41^ used cyclohexenone and NADH with methanol and DMSO as solvents and did not take into account co-factor recycling: while some decrease in yields was observed at 30% levels of co-solvent, 40% data was not included and very low yields were observed at 50% levels.

In addition to the ERs, a recently reported wild type transaminase originating from the same drain metagenome, but from a different bacterium, showed extraordinary organic solvent stability maintaining activity in up to 50% of DMSO demonstrating that valuable enzyme properties can be obtained from metagenomic samples obtained from niche environments.^21^

### Preparative scale reactions and expansion of the substrate scope

The two most promising ERs expressed from pQR1907 and pQR1445, were further investigated for the reduction of more complex and sterically challenging substrates including the bicyclic Wieland–Miescher ketone 8. Decalones derived from 8 are versatile chiral building blocks in the synthesis of natural products such as terpenoids and steroids.^28^ Little has been reported on their biocatalytic reduction presumably due to problems with substrate acceptance. Indeed, the bioreduction of 8 has only been described in a patent using an industrially engineered enzyme generated by gene shuffling of selected OYEs.^29^

Due to the absence of a chromophore in the reduced product, bioreductions of 8 were initially monitored by following the depletion of the starting material. The reduction of *rac*-8 and *S*-8 occurred with the complete conversion of the starting material with the two ERs from pQR1907 and pQR1445 used as purified enzymes (Table 3). The reactions required longer times to proceed compared to the carvone substrates and were run for 20 hours. A kinetic study with *rac*-8 revealed a faster depletion of the *S*-enantiomer with purified pQR1907 (Fig. S6†).

**Table 3:** Bioreductions of *rac*-8 and *S*-8 using the enzymes from pQR1907 and pQR1445^a^

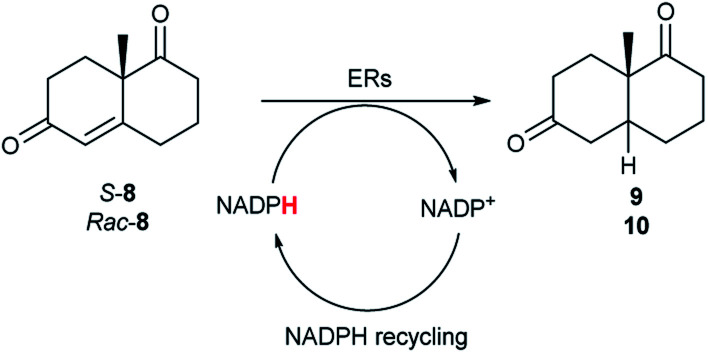		
ER	*S*-8	*Rac*-8
	Conv. [%]	Conv. [%]
pQR1907	>99	>99
pQR1445	>99	97

a Substrate (10 mM), purified ER (0.2–0.9 mg mL^−1^), NADP^+^ (2.8 mM), G6PDH (20 U), G6PNa (100 mM), in Tris–HCl (50 mM) and DMSO (10%) at pH = 7.4, 30 °C, 20 h, 300 rpm. Reactions were performed in triplicate. Conversions were determined by HPLC based on the depletion of starting material.

From this promising data, ERs from pQR1907 and pQR1445, and NCR, for comparison purposes, co-expressed with the recycling system G6PDH, were used as clarified cell lysates with *S*-5 and *S*-8 and the reactions conditions optimized (Fig. S7 and S8†). Biotransformations were then carried out on a preparative 25 mL scale with the initial concentration of 10 mM of *S*-5 and 20 mM of *S*-8 (see Fig. S8 and S9†). Under these conditions the ER expressed from pQR1445 fully converted *S*-5 in an hour affording (2*R*,5*S*)-dihydrocarvone 6 in 95% yield with only traces of the other diastereomer (NMR: d.r. = 98 : 2) (Table 4). The product 6 was readily isolated by extraction from the reaction mixture with ethyl acetate without further purification. The biocatalytic preparative scale reaction with the enzyme expressed from pQR1907 on *S*-8 resulted in a 95% yield of dione 9 in 20 hours. The selectivity and the stereochemistry were determined by ^1^H NMR spectroscopy (*J*-values) (Fig. S10–S16†). Analysis revealed that the reduction occurred with complete selectivity for the *syn*-isomer at the ring junction. Compound 9 was also obtained in high purity after extraction from the reaction mixture with ethyl acetate, without the need for any further purification. Under the same reaction conditions the performance of NCR was inferior to these novel ERs affording 9 in only 16% yield.

**Table 4:** Bioreductions on preparative scale^a^

Substrate	Enzyme (mg mL^−1^)	Product	Isolated yield (%)	d.r.
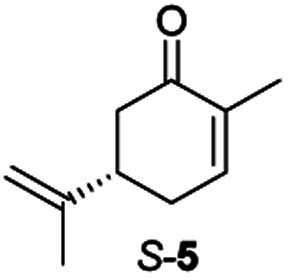	pQR1445 (0.5)	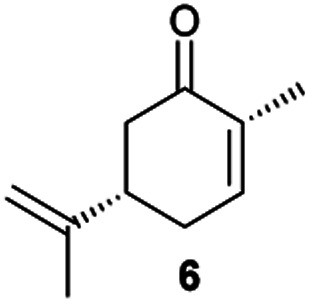	95^b^	98 : 2
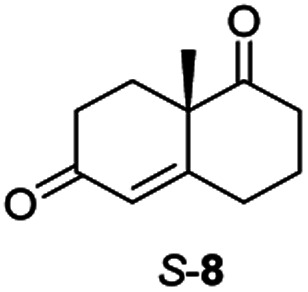	pQR1907 (1)	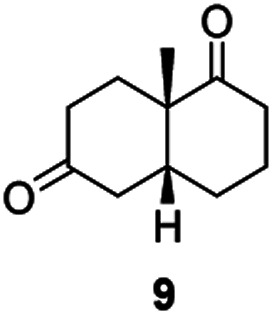	95^b^	*Syn* only
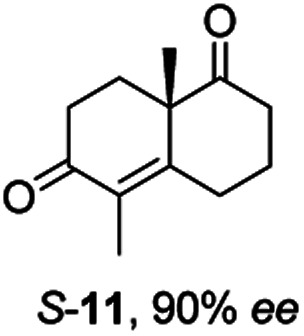	pQR1907 (2)	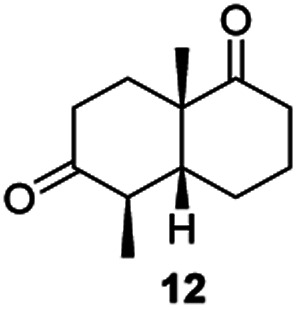	71^b^^,^^c^	*Syn* only
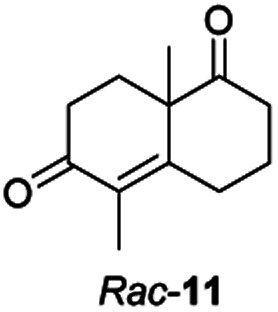	pQR1907 (2)	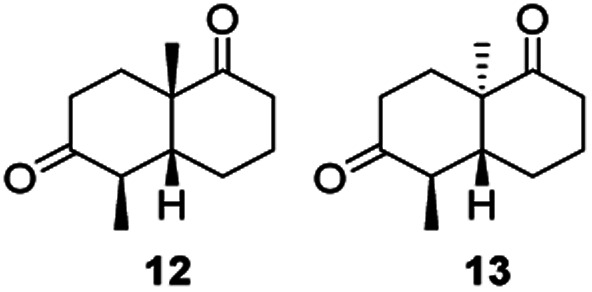	90^d^	58 : 42
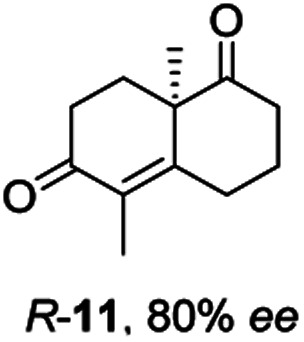	pQR1907 (2)	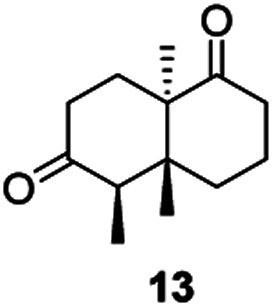	90^d^	85 : 15
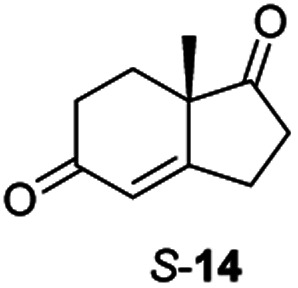	pQR1907 (1)	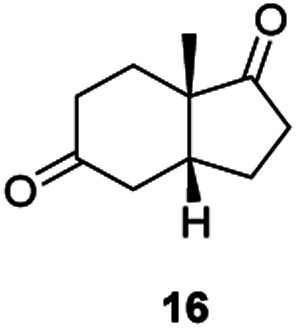	94^b^	*Syn* only
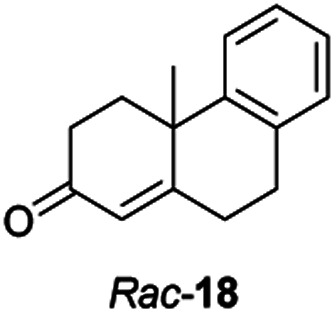	pQR1907 (4)	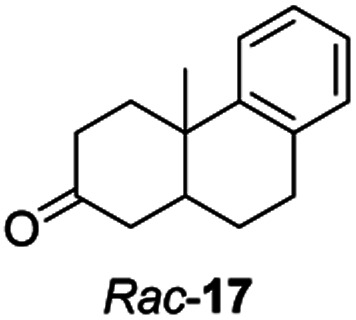	50^d^	—

a Substrate (5 mM for *rac*-16; 10 mM for *S*-5 and *R*-11 20 mM for *S*-8, *S*-11, *rac*-11, *S*-14), co-expressed ER/G6PDH (1–4 mg mL^−1^), NADP^+^ (2.8 mM), G6PNa (100 mM), in Tris–HCl (50 mM) and DMSO (10%) at pH = 7.4, 30 °C, 300 rpm.

b Isolated yield.

c Purification by column chromatography.

d 2.5 mL scale. Quantification by NMR using an internal standard (1,3,5-trimethoxybenzene).

**Fig. 3:**
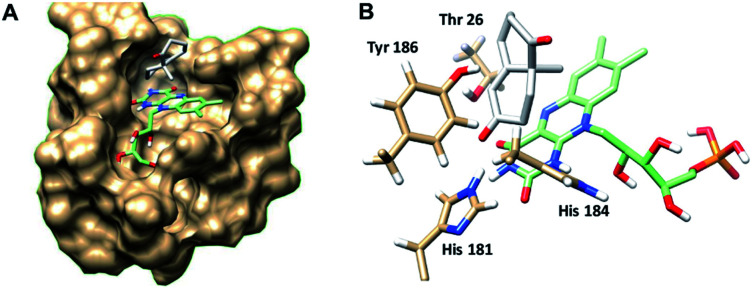
(A) Wieland–Miescher ketone *S*-8 docked in the active site of the ER pQR1907 homology model (residues coloured beige) using Autodock Vina.^32^ (B) Key residues surrounding the substrate *S*-8 (coloured white) and FMN cofactor (coloured green). Images generated using Chimera.^33^

In order to understand better the enzyme performance, molecular modelling was performed using a homology model of the ER pQR1907.^30,31^ Docking of the substrate *S*-8 into the active site was performed and its proximity to the FMN co-factor and the key-residues were determined (Fig. 3) (Table S3†). Notably, *S*-8 readily fitted into the active site close to the FMN co-factor (distance from the alkene to the nitrogen of the FMN ∼3.6 Å) providing the reductive capability. Also, the alkene was ∼3.7 Å from the key Tyr186 residue for protonation. In contrast, docking of *S*-8 into the active site of NCR (crystal structure 4A3U) did not result in a productive mode. These results could help to rationalize the discrepancy of reactivity between the two ERs with *S*-8, where it is readily accepted by the ER from pQR1907, but only low levels of product were formed with NCR.

Informed by the docking results the substrate scope was further explored towards other bicyclic enones using the ER expressed from pQR1907 (Table 4). Compound *S*-11 was prepared using established methods (90% ee),^34^ and was efficiently reduced affording 12 in 71% yield as the *syn* isomer (after column chromatography). *Rac*-11 was also converted into a mixture of 12 : 13 in 90% yield and the reduction afforded a mixture of isomers (d.r. = 58 : 42), highlighting that *R*-11 was converted more slowly. This rate discrepancy could result from preferred stereochemistries in the ER active site. However, by decreasing the substrate loading to 10 mM, *R*-11 (84% ee) was fully converted to the dione 13 with a high selectivity for the compound with the *trans* ring junction (Fig. S15 and S16†). In addition, the Hajos–Parrish ketone *S*-14 could also be readily reduced to give 15 in 94% yield. The product was also isolated by extraction from the reaction mixture with ethyl acetate and no purification step was required. The ^1^H NMR spectroscopic data of 15 revealed total selectivity in the reaction for the *syn* isomer. Finally, taking advantage of the metagenomic enzyme to tolerate higher concentrations of DMSO, a bulkier tricyclic enone *rac*-16 was also synthesized.^35^ Remarkably it was also accepted to give the reduced product *rac*-17 in 50% yield. This suggests that the presence of the aromatic ring may enable both faces of the C=C bond in 16 to be presented to the FMN co-factor. Such reductive reactivity with truncated steroids has not been reported before with wild type ERs and is reminiscent of the activity exhibited by androstenedione reductase, a 3-oxo-5-alpha-steroid 4-dehydrogenase family protein, which shares only 9% protein sequence identity with pQR1907.^36^

## Conclusions

In summary, we have demonstrated the value of using metagenomic data mining for the discovery of novel ERs for use in asymmetric bioreductions. Out of nine ERs from the drain metagenome that were identified, seven have displayed high activity and selectivity towards monocyclic enones demonstrating a high hit rate from the initial *in silico* selection.

Two ERs, from pQR1445 and pQR1907, displayed high activities in up to 30% DMSO and methanol and proved to be extremely efficient in preparative scale reactions, reducing the Hajos–Parish 14 and Wieland–Miescher ketones 8 in very high yields with complete selectivity. Until now, bioreductions of these types of substrates have not been reported with wild type enzymes. Sterically challenging substituted-WMKs 11 and a tricyclic derivative 16 were also converted in good yield and selectivity adding novelty to the substrate scope known for ene-reductases. The metagenomic ERs have significant potential in synthetic applications and could also serve as an excellent starting point for future protein engineering.

## Conflicts of interest

There are no conflicts to declare.

## Supplementary Material

RA-009-C9RA06088J-s001
