# Supplementary material for: Metagenomic ene-reductases for the bioreduction of sterically challenging enones
Source: RSC Adv. 2019 Nov 11;9(63):36608–14. doi: 10.1039/c9ra06088j (PMC9075147; doi:10.1039/c9ra06088j)
Supplement: RA-009-C9RA06088J-s001 [file RA-009-C9RA06088J-s001.pdf]

Electronic Supplementary Material (ESI) for RSC Advances.
This journal is © The Royal Society of Chemistry 2019

Supporting information

Metagenomic ene-reductases for the bioreduction of sterically challenging enones

Dragana Dobrijevic,[a]‡ Laure Benhamou,[b]‡ Abil E. Aliev,[b] Daniel Méndez-Sánchez,[b] Natalie Dawson,[c] Damien Baud,[b] Nadine Tappertzhofen, [b] Thomas S. Moody,[d] Christine A. Orengo, [c] Helen C. Hailes[b]* and John M. Ward[a]*

[a] Department of Biochemical Engineering, University College London, Bernard Katz Building, London WC1H 6BT, UK. E-mail: **j.ward@ucl.ac.uk**. [b] Department of Chemistry, University College London, 20 Gordon Street, London, WC1H 0AJ, UK. E-mail: **h.c.hailes@ucl.ac.uk**. [c] Structural and Molecular Biology, University College London, London, WC1E 6BT, UK. [d] Almac, Department of Biocatalysis & Isotope Chemistry, 20 Seagoe Industrial Estate, Craigavon, BT63 5QD, N. Ireland, UK.

Table of Contents

ENZYME DISCOVERY 4

Metagenome isolation, sequencing and assembly. 4

Cloning of selected genes. 5

Enzyme expression and purification 5

Table S1. Metagenomic ERs. 6

Figure S1. Protein sequence analysis. 7

Figure S2. Protein gels. 10

Gene and the corresponding protein sequences. 11

BIOCATALYSIS 15

General considerations 15

Spectrophotometric assay 15

Table S2: Qualitative results of the spectrophotometric assay. 16

Biotransformations - general protocol with purified ERs 16

Figure S3. Bioreduction of carvone **5**: 20 h reaction time. 17

Figure S4. Bioreduction of carvone **5**: 3.5 h reaction time. 18

Figure S5. Time-course experiment for the bioreduction of *S*-**5.** 18

Figure S6. Conversion profile for bioreduction of *rac*-**8** with pQR1907 19

Biotransformations with clarified cell lysates 19

Figure S7. Bioreduction of *S*-**5**. 20

Figure S8. Bioreduction of *S*-**8**. 21

MOLECULAR DOCKING 22

Table S3. Computational docking output (Autodock Vina) – pQR1907 model with ligands (FMN and *S*-**8**) 22

PREPARATIVE SCALE BIOTRANSFORMATIONS 22

Reactions on a 25 mL scale 22

Synthesis of (2*R,*5*S*)-2-methyl-5-(prop-1-en-2-yl)cyclohexan-1-one (**6**) 22

Synthesis of (4a*R,*8a*S*)-8a-methylhexahydronaphthalene-1,6(2*H*,5*H*)-dione (**9**) 23

Synthesis of (4a*R*,5*R,*8a*S*)-5,8a-dimethylhexahydronaphthalene-1,6(2*H*,5*H*)-dione (12) 24

Synthesis of (3a*R*,7a*S*)-7a-methylhexahydro-1H-indene-1,5(4*H*)-dione (15) 25

Procedure for 2.5 mL scale reaction using an internal standard 25

(4a*R*,5*R*)-5,8a-dimethylhexahydronaphthalene-1,6(2*H*,5*H*)-dione (12 + 13) 25

(4a*R*,5*R*,8a*R*)-5,8a-dimethylhexahydronaphthalene-1,6(2*H*,5*H*)-dione (13) 26

4a-methyl-3,4,4a,9,10,10a-hexahydrophenanthren-2(1*H*)-one (17) 28

Determination of the configuration of 9, 12 and 15 by NMR 30

Figure S9. The overlaid view of experimental (red) and fitted (black) ^{1}H NMR lineshapes of **9** (in $CDCl_3$, 700 MHz, 25 °C). 31

Figure S10. The overlaid view of experimental (red) and fitted (black) ^{1}H NMR lineshapes of **12** (in $CDCl_3$, 700 MHz, 25 °C). 32

Figure S11. The overlaid view of experimental (red) and fitted (black) ^{1}H NMR lineshapes of **16** (in $CDCl_3$, 700 MHz, 25 °C). 33

Figure S12. (a) 1D NOESY spectrum of **9** with a selective excitation of the methyl protons at 1.35 ppm (mixing time 300 ms); (b) ^{1}H NMR spectrum of **9**. 34
Figure S13. 2D NOESY spectrum of **12** in $CDCl_3$ (mixing time 600 ms, 700 MHz) at 25 °C. 36
Figure S14. The overlaid view of experimental (red) and fitted (black) ^{1}H NMR lineshapes of 13 (in $CDCl_3$, 700 MHz, 25 °C).... 38
Figure S15. 2D NOESY spectrum of **13** in $CDCl_3$ (mixing time 600 ms, 700 MHz) at 25 °C. 39
Figure S16. (a) 1D NOESY spectrum of **15** with selective excitation of the methyl protons at 1.24 ppm (mixing time 300 ms); (b) 1H NMR spectrum of **15**.... 40
ANALYTICAL METHODS.... 41
Chiral GC analysis (Cyclohexyl-derivatives).... 41
Table S4. Cyclohexyl-derivatives – retention times. 41
Figure S17. Calibration curve for cyclohexanone (**1**).... 42
Figure S18. Calibration curve for 2-methyl-cyclohexanone (**2**).... 42
Figure S19. Calibration curve for dihydrocarvone (**9**). 42
Chromatograms.... 43
Figure S20. *Rac*-2-methylcyclohexanone (commercial). 43
Figure S21. Transformation of 2-methylcyclohex-2-en-1-one (**2**) with NCR. 44
Figure S22. Transformation of 2-methylcyclohex-2-en-1-one (**2**) with pQR1907.... 44
Figure S23. Transformation of carvone derivative *R*-**5** with NCR. 45
Figure S24. Transformation of carvone derivative *R*-**5** with pQR1445. 45
Figure S25. Transformation of carvone derivative *S*-**5** with NCR. 46
Figure S26. Transformation of carvone derivative *S*-**5** with pQR1907.... 46
Analytical chiral HPLC (Wieland-Miescher ketone consumption) 47
Figure S27. Calibration curve for Wieland-Miescher ketone **8**.... 47
Figure S28. HPLC chromatogram - *rac*-**8**. 48
Figure S29. HPLC chromatogram - *R*-**8** 48
Figure S30. HPLC chromatogram - reduction of *S*-**8** with pQR1907 (no starting material remaining). 48
NMR spectra.... 49
Synthesis of (2*R*,5*S*)-2-methyl-5-(prop-1-en-2-yl)cyclohexan-1-one (6) 49
Synthesis of (8a*S*)-8a-methylhexahydronaphthalene-1,6(2*H*,5*H*)-dione (9) 50
Synthesis of (4a*R*,5*R*,8a*S*)-5,8a-dimethylhexahydronaphthalene-1,6(2*H*,5*H*)-dione (12) 51
Synthesis of (3a*R*,7a*S*)-7a-methylhexahydro-1*H*-indene-1,5(4*H*)-dione (15).... 53
REFERENCES.... 55

ENZYME DISCOVERY

Metagenome isolation, sequencing and assembly.

Total DNA was isolated from a sample collected from a domestic shower drainpipe. Hair blocking the drain was removed and a surrounding gel-like liquid mass (20 mL) was added to buffer (25 mM Tris-HCl, 150 mM NaCl and 25 mM EDTA) making the total volume of 250 mL. The sample was warmed to 60 °C for few minutes and 80 mL of phenol was added (buffered phenol solution, Sigma). The sample was mixed well and left, with occasional mixing, in a 65 °C water bath for 45 min, then centrifuged for 20 min at 7,000 rpm to separate the phases. The top aqueous phase was removed into two large centrifuge bottles and an equal volume of isopropanol was added to each. Samples were left on ice for 10 min then centrifuged for 10 min at 7,000 rpm. Each pellet was resuspended in 10 mL of TE buffer (10 mM Tris-HCl pH 7.5, 1 mM Na_2EDTA) and added to 10.7 g of cesium chloride (CsCl). Ethidium bromide (EtBr) was added to a final concentration of 20 µg/mL. The solutions were transferred into Beckman ultracentrifuge tubes and centrifuged at 40,000 rpm for 48 h to form a density gradient; clean gradients were visible. The DNA containing band in the bottom third of the tube was removed under UV illumination and the EtBr extracted with isopropanol saturated with CsCl solution. The DNA solutions (both approximately 2.2 ml) were diluted to 10 mL with TE buffer and 0.1 volumes of 5 M NaCl and 2 volumes of ethanol were added. A large white DNA precipitate formed at the interface after 20 mL of ethanol was layered onto the surface before mixing. The solution was left on ice for approx. 30 min, giving a clot of DNA after mixing. This was centrifuged for 10 min at 15 °C, each pellet was dried and resuspended in 2 ml of TE buffer.

Library preparation and sequencing were performed at UCL Genomics (University College London, UK). One run on Illumina MiSeq sequencer (paired–end 250 bp) generated 13,683,797 paired–end reads totaling 14.2 GB. Raw reads were submitted to the European Nucleotide Archive under sample accession number ERS811192. PERF, PETKit v1.1b (http://microbiology.se/software/petkit) was used for quality filtering. 11,127,204 quality checked reads were assembled by IDBA-UD 1.1.2 [1] using a kmer size iterated from 80 to 120. On assembled contigs, genes were predicted by Prodigal v2.6.2 by running it in the metagenomic mode.[2] Corresponding protein sequences were annotated by scanning the sequences against Pfam28.0 libraries of domain families by PfamScan. [3] Overlapping domain assignments were resolved by DomainFinder3 as previously described.[4] The completeness of the candidate gene and the corresponding protein sequence was assessed by BLASTP searches against the non-redundant GenBank database. Theoretical molecular weights and extinction coefficients were calculated with the ProtParam tool (ExPASy).

Cloning of selected genes.

Metagenomic ERs were PCR amplified directly from the drain metagenomic DNA and cloned into pET29a(+) (Novagen, Merck). Genes were cloned to start with an ATG within the *NdeI* restriction site and in fusion with a C-terminal Hisx6 tag. Resulting expression plasmids were verified by DNA sequencing (Eurofins Genomics). A gene encoding N-terminal Hisx6 - tagged enzyme nicotinamide-dependent cyclohex-2-en-1-one reductase (NCR) from *Zymomonas* mobilis (UniProtKB Accession: Q5NLA1)[5] was codon optimized for expression in *E.coli*, synthesized and cloned into pJ411 vector by DNA2.0, USA. The gene for the cofactor recycling enzyme glucose-6-phophate dehydrogenase from *Saccharomyces cerevisiae* SF838 (UniProtKB Accession: P11412) was amplified from genomic DNA and cloned into pACYCDuet (Novagen, Merck) using *Bam*HI and *Sal*I restriction sites.

Enzyme expression and purification.

All plasmids were transformed into *E. coli* expression strain BL21 (DE3). Precultures were grown overnight at 37 °C in Terrific broth (TB) containing kanamycin (50 µg/mL) and were used to inoculate fresh TB media (1% v/v). Cells were grown at 37 °C under shaking to an OD_{600} of 0.4 – 0.6. Protein expression was induced by addition of IPTG (final concentration 1 mM). Cells were harvested by centrifugation after 48 h at 16 °C. Pellets were thawed in the binding buffer (20 mM sodium phosphate, 0.5 M NaCl, 5 mM imidazole, pH 7.4) supplemented with an excess amount of flavin mononuceotide (FMN, final concentration 0.1 - 0.2 mg/mL). Cells were disrupted by sonication with cooling on ice, and then incubated on ice for another 30 min, to achieve high flavination. Cell debris was removed by centrifugation (12,000 rpm, 45 min, 4 °C). Supernatant was filtered through a 0.45 mm PES filter and loaded, at room temperature, onto Ni^{2+} Sepharose resin (Sepharose Fast Flow, GE Healthcare) equilibrated with the binding buffer (20 mM sodium phosphate, 0.5 M NaCl, 5 mM imidazole, pH 7.4). Protein was eluted by a step gradient with the following concentration of imidazole: 50, 100 and 500 mM in 20 mM sodium phosphate, 0.5 M NaCl, pH 7.4. Proteins were precipitated directly in the elution buffer with ammonium sulfate (final concentration 3.2 M) and stored at 4 °C. The purity of the recombinant proteins was checked by SDS-PAGE and protein concentration of desalted protein determined by A_{280}. Clarified lysates were prepared from BL21 (DE3) *E. coli* co-expressing the ER and the cofactor recycling enzyme glucose-6-phophate dehydrogenase. Precultures were grown at 37 °C in TB containing kanamycin (50 µg/mL) and chloramphenicol (34 µg/mL). Protein expression and cell harvesting were performed as described earlier. Pellets were resuspended in 50 mM Tris-HCl buffer pH 7.5, the cells disrupted by sonication, cell debris removed by centrifugation and supernatant was filtered through a 0.45 mm PES filter. The protein concentration of the clarified cell lysate was determined by Bradford protein assay with bovine serum albumin as a standard (Bio-Rad). Clarified lysates were stored in small batches at −80 °C.

Most of the enzymes were well expressed and soluble except pQR1439, pQR1440, pQR1442 and pQR1443 that gave lower yields of soluble protein using the standardised conditions used here. All proteins were purified by Ni-NTA chromatography as bright-yellow co-factor-enzyme complexes.

Table S1. Metagenomic ERs.

pQR	Length (amino acids)	MW (kDa)	**Closest homologue in GeneBank** (BLASTP, October 2019)			
			**Putative protein annotation**	**Phylum**	**Bacteria**	**% Identity**
**1439**	426	47.1	NADH:flavin oxidoreductase/NADH oxidase family protein	*Proteobacteria*	*Fluviicoccus keumensis*	76
**1440**	367	39.3	alkene reductase	*Proteobacteria*	*Sphingopyxis*	95
**1442**	372	40.5	NADH:flavin oxidoreductase/NADH oxidase	*Proteobacteria*	*Azonexus fungiphilus*	99
**1443**	387	42.7	12-oxophytodienoate reductase	*Proteobacteria*	*Sphingomonadales bacterium*	95
**1445**	355	37.9	alkene reductase	*Proteobacteria*	*unclassified Pseudomonas*	77
**1446**	375	41.3	NADH:flavin oxidoreductase	*Proteobacteria*	*Pseudomonas sequence ID: WP_061903666.1*	100
**1907**	370	40.5	alkene reductase	*Proteobacteria*	*Moraxellaceae bacterium*	85
**1908**	362	38.4	alkene reductase	*Proteobacteria*	*Acidovorax sp. 62*	93
**1909**	374	40.1	oxidoreductase	*Proteobacteria*	*Stenotrophomonas acidaminiphila*	98

Figure S1. Protein sequence analysis.

Multiple sequence alignments of metagenomic ERs and selected OYE family members showing shared conserved residues (**A**) and phylogenetic classification (**B**).

A

```
OYE1          MSFVKDFKPQALGDTNLFKPIKIGN-NELLHRAVIPPLTRMRALHPGNIPNRDWAVEYYT    59
pQR1440       ------------MAVSLFDPIKLGA-IDAPNRIIMAPLTRGRAG-PGFVPTE-LARDYYR    45
pQR1445       -------------MSNLFTPLQVGA-WQLPNRIIMAPLTRCRAS-EGRVPNA-LMAEYYA    44
pQR1908       ------------MHSSLFNPLQVGA-LTVPNRILLAPLTRARAD-AGHMPNA-LMAEYYA    45
pQR1907       ------------MSGKLFTPFSSGS-FTFPNRVIMAPLTRMRASQPGDIPNE-LMQTYYV    46
YqjM          ------------MARKLFTPITIKD-MTLKNRIVMSPMCMYSSHEKDGKLTP-FHMAHYI    46
pQR1442       -------------MSALFSNFKLKD-ITLRNRIAIPPMCQYSAVD--GLVND-WHRVHYA    43
pQR1909       -------------MSRLFSPLALGP-LPLSNRIVIAPMCQYSADE--GRATD-WHAFHWP    43
Ppo-ER3       -----------MNTELLFKPFKAGN-LSLPNRIVMAPMTRNFS--PQGIPGP-EVAAYYR    45
pQR1443       -MTTAPLDDLAALLAPLHAPFTCKS-LKAPNRFCMAPMSRYFA--PGGVLSD-EGAEYYR    55
pQR1446       ---------MTAPVQALFAPFRLGN-LELPTRVVMAPMTRSFS--PGGVPNA-QVVEYYR    47
YqiG          ---------MNPKYKPLFEPFTFKSGVTIDNRIAVAPMTHYASNEDGTISEA-ELDYIIP    50
pQR1439       ------MTKQLSPAQVLAQPFSLPNGSIIKNRLAKSAMSESMGTYDNRVTPG-LV-RLYD    52
                              *   :          *     :    .

OYE1          QRAQRPGTMIITEGAFIS-PQAGGYDNAPGVWSEEQMVEWTKIFNAI-HEKKSFVWVQLW    117
pQR1440       QRAS--AGLIISEATGIS-QEGLGWPSAPGLWTDAQVEGWKPVTDAV-HAAGGRIVAQLW    101
pQR1445       QRAS--AGLIISEATSVT-PMGVGYPNTPGIWSDAQVDGWKLITDAV-HQAGGRIVLQLW    100
pQR1908       QRAS--GGLLITECTMVA-PNTSAFIAEPGIYSPEQVAAWKQVTSAV-HAKGGRIYMQIW    101
pQR1907       QRAS--AGLIIAEATQIS-PQGKGYMDTPGIYSAEQVQGWRKITQAV-HEAGGHIALQLW    102
YqjM          SRAIGQVGLIIVEASAVN-PQGRITDQDLGIWSDEHIEGFAKLTEQV-KEQGSKIGIQLA    104
pQR1442       TLARGGAGLVIVEATGVA-PEGRITPACLGLWNDAQATELAKLAAAI-KAGGAVPGIQIG    101
pQR1909       NLAQSGAALAIIEATAVE-PRGRISWADLGLWDDTTEAAFARALAAARRYSTMPIGVQLA    102
Ppo-ER3       RRAENAVGLIITEGTAINHPAAVEHTSIPNFYGE-GLEGWAKVVEEV-HAVGGKIIPQLW    103
pQR1443       RRAAAGIGTIITEGTGVAIDHTVAADTVPIFAGDTPLAAWKGAVDAV-HAEGGMFVPQLW    114
pQR1446       RRAAAGVGLIVTEGTTVGHKAANGYPHVPRFYGEDALAGWKQVVDAV-HAEGGKIVPQLW    106
YqiG          R--SKEMGMVITACANVT-PDGKAFPGQPAIHDDSNIPGLKKLAQAI-QAQGAKAVVQIH    106
pQR1439       RWADGGIGLCITGNVMID-KRALGEPGNVVVEDESDLDMLKAWAEAG-TRNGTQLWMQIN    110
                        :     :             .                          *:

OYE1          VLGWAAFPDNL-----------ARDGLRYDSAS-----DNVFMDAEQEAKAKKANNPQHS    161
pQR1440       HMGRVVHSVF------------ND-GKPPVSASATQAPGKAHT-----PVGRLDYEVARP    143
pQR1445       HVGRISDPVY------------LD-GQLPVAPSAIAPQGHVSL-----VRPTKAFETPRA    142
pQR1908       HAGRAAHPAI------------NG-GTPTVSSTATPIEGDIHT-----PTGKVPHVPAHV    143
pQR1907       HVGRVSHHSLQ-----------PD-QQLPVSASAIPYQNRTTVRGEDGKPTRVDCDTPRA    150
YqjM          HAGRKAELEGD------IFA-----------PSAIAFDEQS--------------ATPVE    133
pQR1442       HAGRKASANRPWEGDD-HIADGDPRGWATLSPSAVAFGANL-------------AKVPKA    147
pQR1909       HAGRKASTHRPWEHHGAQIAPDAPQGWRTVSASSLPYAEGQ--------------HPPDS    148
Ppo-ER3       HVGTARKIGAD---------NQPNPEALPVGPSGISPAG---------------EKVVEP    139
pQR1443       HVGGCIDFNFP---------DSPHAE--LVSPSGFAGPD---------------VPGGRA    148
pQR1446       HVGNVRKAGTE---------P--DASVPGYGPSEKVKDG---------------TVVVHG    140
YqiG          HGGIECPSELV---------P----QQDVVGPSDV-----F----------DNGKQIARA    138
pQR1439       HPGKQVMRTLV---------S----DP--VAPSAIPFGKEM----------QAFFATPRA    145
                *                             :

OYE1          LTKDEIKQYIKEYVQAAKNSIAAGADGVEIHSANGYLLNQFLDPHSNTRTDEYGGS-IEN    220
pQR1440       LELGEIPRVIADYAKAAENAKRAGFDGVQLHGANGYLIDQFLRDGSNLRDDDYGGP-IEN    202
pQR1445       LDTAELVDVVAAYRLGAENAKKAGFDGVEIHGANGYLLDQFLQSSTNQRTDQYGGS-LEN    201
pQR1908       LTESEIPGIVEAFVQGAKNAIAAGFDGVEVHAANGYLIDQFLRDGANDRTDGYGGP-LEN    202
pQR1907       LELSEIPGVIEDYRRATVNSREAGFDMVEVHAAHGYLLHQFQSAESNKREDAYGGS-LEN    209
YqjM          MSAEKVKETVQEFKQAAARAKEAGFDVIEIHAAHGYLIHEFLSPLSNHRTDEYGGS-PEN    192
pQR1442       MTFEDIARVRDDFVAAAKRALDAGFEWLELHFAHGYLAQSFFSVHANQRTDQYGGD-YAG    206
pQR1909       LDQAGIDAIIAAFADSAHRAVRLGLDLIEIHAAHGYLLHQFLSPLSNRRDDAYGGA-LEN    207
Ppo-ER3       LTEAEIADIISAYAQAAADAQRVGFDGIELHGAHGYLIDQFFWDKTNKRTDQYGGN-LVQ    198
pQR1443       MTERDIADTAAAFAESARAAKEIGCDAIELHGAHGYIFDQFFWDRTNFRDDRYGGPDIGD    208
pQR1446       MTKDDIQEVIAAFAQAARDAKAIGMDGVEIHGAHGYLIDQFFWEGSNKRTDEYGGD-LAQ    199
YqiG          LKEEEVENIVKAFGEATRRAIEAGFDGVEIHGANGYLIQQFYSPKTNQRTDRWGGS-DEK    197
pQR1439       LTEAEIEDIIQRFGRTAAIAKKAGFSGVQIHGAHGYLVSQFLSGHHNQRDDRWGGN-LEN    204
              :    :      :   :  :   * . :::* *:**:  .*     * * * :**

OYE1          RARFTLEVVDALVEA----IGHEKV-GLRLSPYGVFNSMS--GGAETGIVAQYAYVAGEL    273
pQR1440       RIRLLREVTEALISV----WGADRV-GVRLSPNGDTQGVD--DSAPEQ---LFPVAAAAL    252
pQR1445       RARLLLEVVDACIEV----WGADRV-GVHLAPRGDAHDMG--DANPAE---TFGYVAEQL    251
pQR1908       RARFLFEVLTAVTAA----IGSDRV-GVRLSPLNSFNSMK--DSDPIA---FIGFLAEKL    252
pQR1907       RARLTLEALDAVIGA----WDAKHV-GIRISPLGTFNGLD--DKDGLE---MALYLTREF    259
YqjM          RYRFLREIIDEVKQV----WDGPL--FVRVSASDYTDKG----LDIADHIGFAK------    236
pQR1442       RSRFLLETLAAVREV----WPEHLPLTARFGVIEFDGRD---EETLAESIDLSV------    253
pQR1909       RMRLVLQVFDAIKAV----VPASMAVGVRISATDWVDGG----WDLAQSIALAK------    253
Ppo-ER3       RTRFAVEVIEACRRA----VGPNFPIVLRFSQWKMYHYEEKLAQTPQE---LEQFLT---    248
pQR1443       RATFAAEVVAACREA----VGEDFAIIMRVSQWKTYDYDVKLARDPDE---MHRWLD---    258
pQR1446       RSRFAIELIQAVRAA----VGPDFPIIFRFSQWKQQDYTARLVQTPEE---LGAFLK---    249
YqiG          RLAFPLAIVDEVKKAASEHAKGAFLVGYRLSPEEPETPG----LTMTETYTLVD------    247
pQR1439       RRRFVLEVYREMRAR----VGKGFPIGIKLNSADFQKGG----FTEEESLDVIR------    250
              *  :                        :.
```

```
OYE1        EKRAKAGKRLAFVHL-------VEPRVTNPFLTEG----EGEYEGGSNDFVYSIWKGPVI    322
pQR1440     DA-----LGIAFLEL-------REPGPEGTFGRTD----VPKQSP----AIRAAFKGPLI    292
pQR1445     GA-----RQVAFICT-------REYLADDS------------LAG----LIKAKFGGVYI    283
pQR1908     NA-----FKPAYLHV-------MRA----DFLQAQ----HGDVLS----VARAKYHGVLV    288
pQR1907     TK-----RGIAYLHL-------SEPDWAGG--PAH----GDEFRQ----ALRDAFPGTII    297
YqjM        -WMKEQGVDL-IDCSSGALVH-ADINVFPGYQVS--------FAE----KIREQADMATG    281
pQR1442     -AFRKAGLDM-LNVSVGFSTLKANIPWAPAFLAP--------IAE----KVRSATGMPVA    299
pQR1909     -ALDARGSHF-IHVSSGGLHPAQKIALGPGYQVP--------FAA----AIKREVAMPVI    299
Ppo-ER3     -PLVKAGVD-IFHCS-------SRRFWEPEFEGSD-----LNLAA----WTKKITGKPVI    290
pQR1443     -PMVRAGVD-IFHAS-------QRRFWEPEYAGDP-----KNLGG----WIKEVTGKPVI    300
pQR1446     -PLSDAGVD-IFHCS-------TRRFWEPEFEGSD-----LNLAG----WTRQLTGKPTI    291
YqiG        -ALGDKELDY-LHISLMDVNSKA------RRGADPTRTRMDLL---NE---RVGNKVPLI    293
pQR1439     -ALSDAGIDH-IEVSGGTYEAPVMAGKKNRFVKDSTRRREAYFLEFAEKARQAVPEMPLM    308

OYE1        RAGNFALHPEV-------------------------VREEVKDKRTLIGYGRFFISNPDL    357
pQR1440     LNSDYDVAKAE--------------------------AALADGLADAIAFGRPFIGNPDL    326
pQR1445     ANEKYDQAQAD--------------------------AAIASGAADAVAFGVKFIANPDL    317
pQR1908     GNMGYSAEEAE--------------------------QAIAGGKLDAVAFGTSFLANPDL    322
pQR1907     GAGNYTVEKSE--------------------------MLLAKGFIDAAAFGRPFIANPDL    331
YqjM        AVGMITDGSMA-------------------------EEILQNGRADLIFIGRELLRDPFF    316
pQR1442     SSWGIDMPATA-------------------------ERVVAEHQMDLVMIGRAHLANPHW    334
pQR1909     AVGLITAPQQA-------------------------EDILLQAQADAIAIARAVLYDPRW    334
Ppo-ER3     TVGSIGLEKAFLSDLEKNNNRQTDQSSSVEARLEQLVGQVEREEADLVAVGRALLVDPAF    350
pQR1443     TVGSIGMDRDLMQDFVEGIS------SPMLGRLEDLVAMFDRREFDLVALGRVLLADPNW    354
pQR1446     TVGSVGLDGEFLQFMVNTDK------VAEPASLENLLERLNKQEFDLVAVGRALLVDPDW    345
YqiG        AVGSIHSADD--------------------------ALAVIENGIPLVAMGREILVDPDW    327
pQR1439     VTGGFRSSAGM-------------------------AEALTGGAVDFVGVARSLAIEPDL    343
                                                              .      :*

OYE1        VDRLEKGL--PLNKYDR--------------DTFYQ-M----------SAHGYIDYPTYE    390
pQR1440     VERIRNGA--EWAADNP--------------QTWYS-P----------GPEGYTDYPALQ    359
pQR1445     PARFAQGA--VLNAPDP--------------STFYG-A----------GSKGYTDYLTL-    349
pQR1908     PERIQAGA--ALNAPNP--------------NTFYS-P----------GPVGYTDYPTMA    355
pQR1907     PVRLQKGA--ELNNVVA--------------ATLYG-G----------GAEGYTDYPALA    364
YqjM        ARTAAKQLNTEIPAPV-----------------QYERG----------W-----------    338
pQR1442     AYQAAKVLGVEKPSWVL--------------PAPYA-H----------WLERYSANV---    366
pQR1909     PWHAAASLGETIAIAP-----------------QYLRS----------APREVAASFVEA    367
Ppo-ER3     AVKLRDQQIEEIIPYSD--------------EVLKT-L----------N-----------    374
pQR1443     LEKVEQRRIDELTAYDR--------------ATALK-H----------YEHG--------    381
pQR1446     AVKVRDGRESDILPFSR--------------EALKQ-L----------V-----------    369
YqiG        TVKVKEGREKQIET--VIKGT-DKEKYHLPEP----------------------LWQAI-    361
pQR1439     PNRLLAGKEARHAVKDITTGIPMVDKMAMMEVMWYSRQLHRMGNGRRPRPNESGLWSLLA    403

OYE1        EALKLGWDKK-------    400
pQR1440     TA---------------    361
pQR1445     -----------------    349
pQR1908     G----------------    356
pQR1907     -----------------    364
YqjM        -----------------    338
pQR1442     -----------------    366
pQR1909     T----------------    368
Ppo-ER3     -----------------    374
pQR1443     -----------------    381
pQR1446     -----------------    369
YqiG        -VNTPGWVPYKD-----    372
pQR1439     TLAENGWGTLQTRRLRA    420
```

Catalytically important active site residues are highlighted in green, dark grey (classical) and light grey (thermophilic-like). The alignments were generated in Clustal Omega with default parameters.[6] Published sequences used for the analysis: **OYE1**, *Saccharomyces pastorianus*, **Q02899** (UniProt); **YqjM**, *Bacillus subtilis* 168, **BAA12619** (NCBI); **Ppo-ER3,** *Paenibacillus polymyxa*, **MK257767** (NCBI); **YqjG**, *Bacillus subtilis* 168, **BAA12582** (NCBI).

B

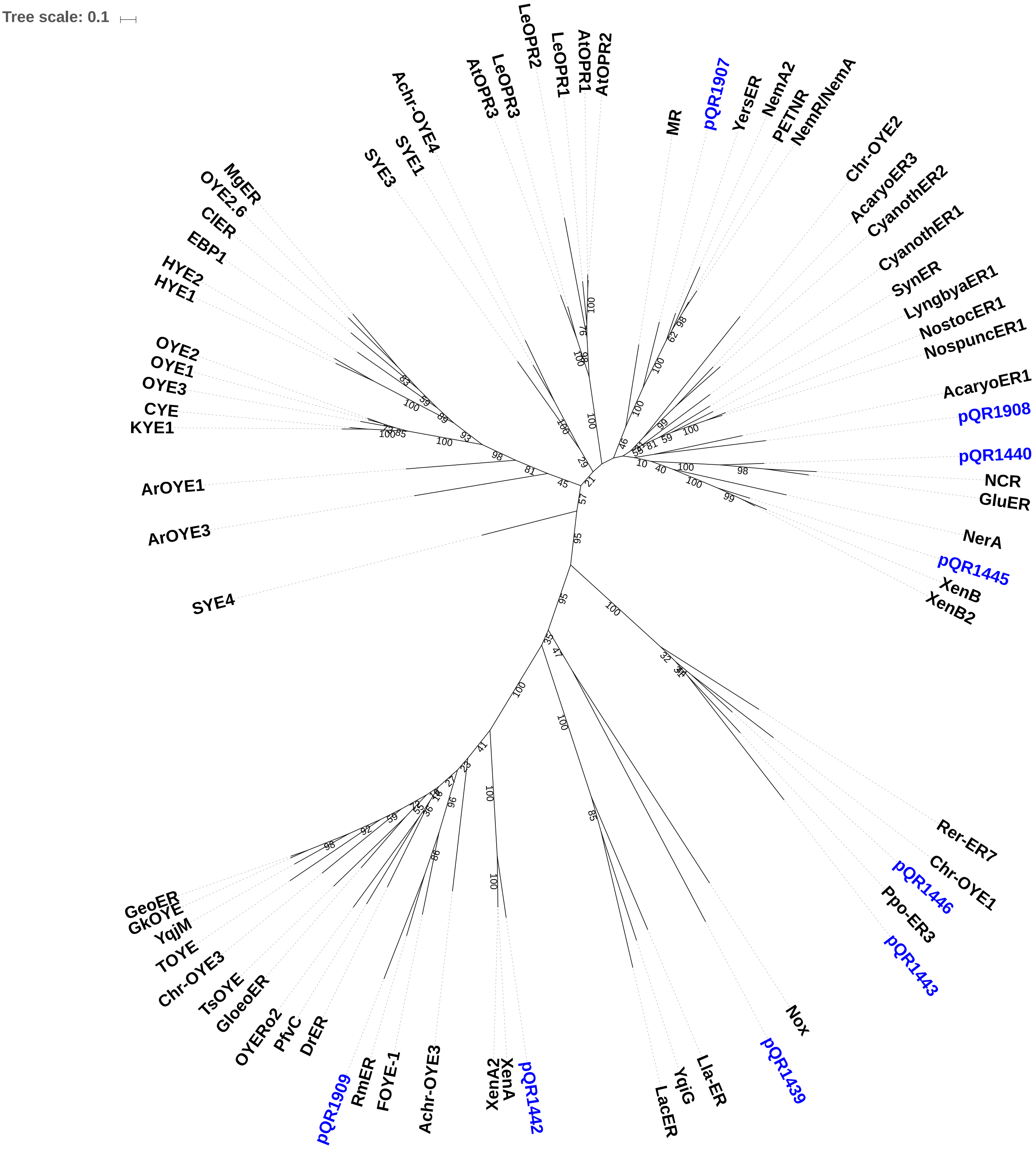

Unrooted phylogenetic tree of OYE family members was computed with MEGA7[7] (ClustalW was used for multiple sequence alignment with default parameters; maximum likelihood distance tree was run with default parameters and 100 replicates (bootstrap values are shown)); tree was visualized with iTOL.[8] Protein accessions and name abbreviations of 63 characterized eukaryotic and prokaryotic OYEs were taken from Scholtissek et al.[9] Protein sequences of PpO-ER3, Rer_ER7, and Lla-ER are from Peters et al.[10]

Figure S2. Protein gels.

(**A**) SDS-PAGE: metagenomic ERs (cell-free lysates). (**B**) SDS-PAGE: metagenomic ERs (purified). (**C**) Native-PAGE: purified metagenomic ERs adopt range of oligomeric states. (**D**) SDS-PAGE: “empty” vector - pET29a(+), glucose-6-phophate dehydrogenase (G6PDH, MW 57.5 kDa), co-expressed pQR1445/G6PDH and pQR1907/G6PDH (cell-free lysates). (**E**) SDS-PAGE: NCR (cell-free lysate and purified protein, MW 39.5 kDa).

SDS-PAGE was run on 12% Mini-PROTEAN® TGX™ Precast Gels (Bio-Rad). Gels A, D, E: protein MW marker (11–190 kDa, NEB #P7706S); Gel B: protein MW marker (14.4 – 116 kDa, Thermo Scientific #26610). Native-PAGE was run on 7.5 % Mini-PROTEAN® TGX™ Precast Gels in Tris/glycine buffer (all from Bio-Rad), NativeMark™ Unstained Protein Standard (20–1,200 kDa), BSA (bovine serum albumin, monomer MW 66.5 kDa) was run as a control.

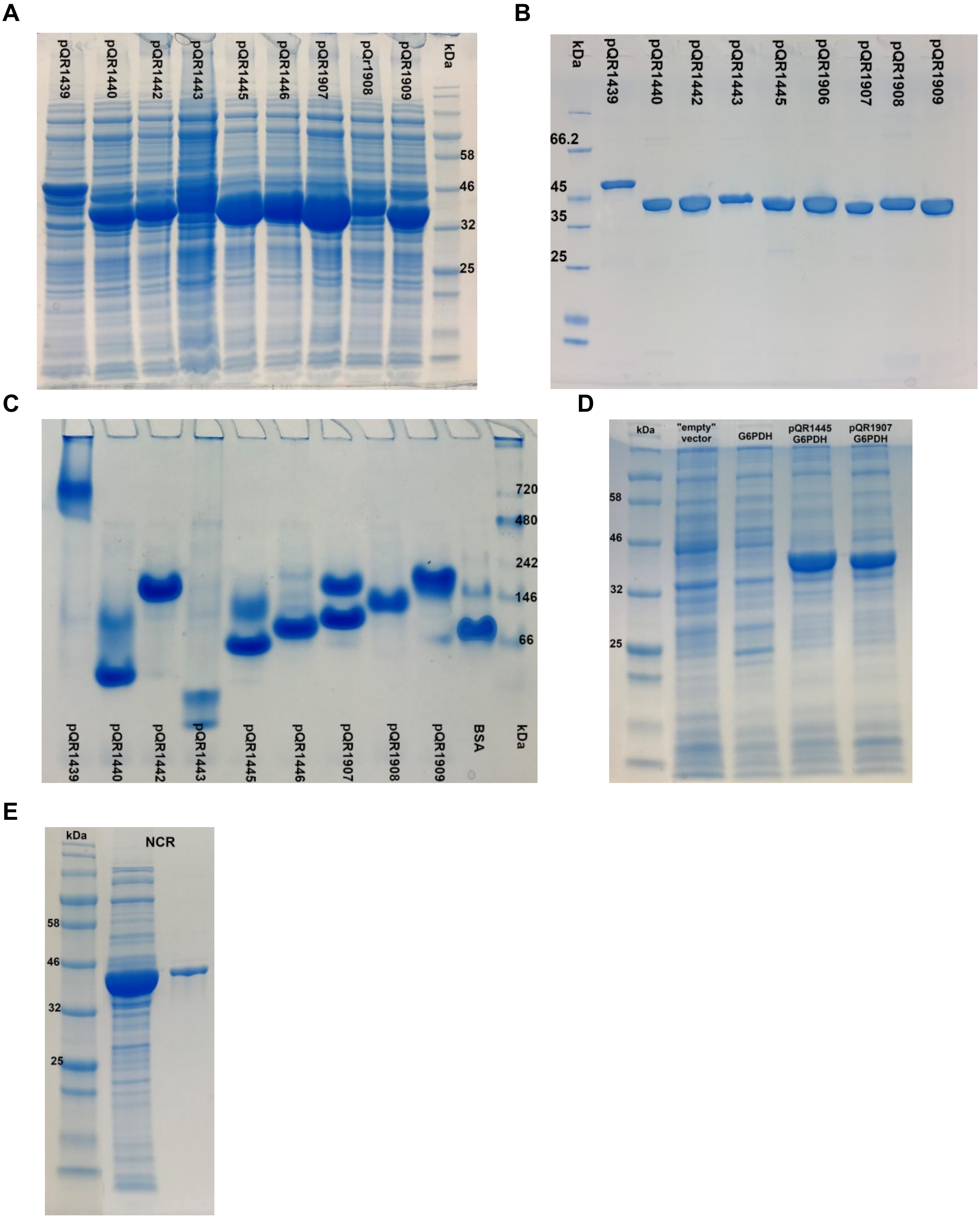

Gene and the corresponding protein sequences.
Nucleotides corresponding to the vector pET29-a (+) are in bold.

>pQR1439
ATGACAAAACAGCTTTCCCCGGCCCAGGTTCTGGCCCAGCCCTTCAGCCTGCCCAACGGCAGCATCATCA
AGAACCGCCTGGCCAAATCGGCCATGAGCGAATCCATGGGGACTTACGACAACCGCGTCACGCCCGGCCT
GGTGCGTCTTTACGATCGCTGGGCCGATGGCGGCATCGGCCTCTGCATCACTGGCAACGTGATGATAGAC
AAGCGCGCTCTGGGCGAGCCCGGCAATGTCGTGGTCGAAGATGAAAGCGATCTGGACATGCTCAAGGCCT
GGGCCGAAGCCGGCACGCGCAACGGCACCCAGCTCTGGATGCAGATCAATCATCCCGGCAAACAGGTGAT
GCGCACCCTGGTGTCCGATCCGGTGGCGCCTTCCGCCATTCCTTTCGGCAAGGAAATGCAGGCGTTTTTC
GCCACGCCGCGCGCGCTCACCGAAGCAGAAATCGAAGACATCATCCAGCGTTTCGGCCGCACCGCCGCCA
TTGCCAAGAAGGCCGGTTTTTCCGGCGTGCAGATTCATGGCGCCCACGGCTACCTGGTCAGCCAGTTCCT
TTCCGGCCACCACAACCAGCGCGATGACCGCTGGGGCGGCAATCTGGAAAACCGTCGCCGTTTCGTGCTC
GAGGTTTACCGCGAAATGCGCGCCCGCGTCGGCAAGGGTTTCCCCATCGGCATCAAGCTGAATTCGGCCG
ACTTCCAGAAAGGCGGTTTCACCGAAGAAGAATCGCTGGACGTGATACGCGCCCTCAGCGATGCCGGCAT
CGACCATATCGAAGTGTCGGGCGGTACCTATGAAGCCCCGGTCATGGCCGGCAAGAAAAACCGTTTCGTG
AAGGATTCCACGCGTCGTCGCGAAGCCTACTTCCTCGAGTTTGCGGAAAAGGCCCGCCAGGCCGTGCCCG
AGATGCCGCTGATGGTCACGGGTGGTTTCCGCAGTTCCGCGGGCATGGCGGAAGCCCTGACCGGTGGTGC
CGTGGATTTCGTGGGCGTGGCCCGTTCCCTGGCCATTGAGCCCGACCTGCCCAACCGCCTGCTGGCCGGC
AAGGAAGCGCGCCATGCCGTGAAGGACATCACCACCGGCATTCCCATGGTGGACAAGATGGCCATGATGG
AAGTGATGTGGTACTCGCGCCAGCTGCACCGCATGGGCAATGGCCGCCGTCCGCGTCCCAACGAGTCCGG
CCTGTGGTCGCTCTTGGCCACGCTGGCGGAAAACGGCTGGGGCACCCTGCAGACGCGTCGCCTGCGCGCC
CACCACCACCACCACCACTGA

>pQR1439
MTKQLSPAQVLAQPFSLPNGSIIKNRLAKSAMSESMGTYDNRVTPGLVRLYDRWADGGIG
LCITGNVMIDKRALGEPGNVVVEDESDLDMLKAWAEAGTRNGTQLWMQINHPGKQVMRTL
VSDPVAPSAIPFGKEMQAFFATPRALTEAEIEDIIQRFGRTAAIAKKAGFSGVQIHGAHG
YLVSQFLSGHHNQRDDRWGGNLENRRRFVLEVYREMRARVGKGFPIGIKLNSADFQKGGF
TEEESLDVIRALSDAGIDHIEVSGGTYEAPVMAGKKNRFVKDSTRRREAYFLEFAEKARQ
AVPEMPLMVTGGFRSSAGMAEALTGGAVDFVGVARSLAIEPDLPNRLLAGKEARHAVKDI
TTGIPMVDKMAMMEVMWYSRQLHRMGNGRRPRPNESGLWSLLATLAENGWGTLQTRRLRA
HHHHHH

>pQR1440
ATGGCCGTATCACTATTCGATCCGATTAAACTGGGCGCGATCGACGCCCCGAACCGCATCATCATGGCGC
CGCTGACGCGTGGCCGCGCCGGACCGGGCTTCGTGCCCACCGAACTCGCACGCGACTATTATCGTCAGCG
CGCTTCGGCGGGGCTCATCATCTCCGAAGCCACGGGCATTTCGCAGGAAGGCCTCGGCTGGCCGAGCGCG
CCGGGCCTGTGGACCGATGCGCAGGTTGAAGGCTGGAAGCCGGTGACCGACGCCGTCCACGCCGCCGGCG
GTCGCATCGTCGCACAGCTTTGGCATATGGGCCGCGTCGTCCATTCGGTGTTCAACGATGGGAAGCCGCC
GGTTTCGGCGTCGGCAACGCAGGCGCCGGGCAAGGCGCACACGCCCGTGGGCCGGCTCGATTATGAAGTC
GCGCGGCCGCTGGAGCTTGGCGAGATCCCGCGGGTGATAGCCGATTATGCCAAGGCGGCCGAAAATGCGA
AAAGGGCCGGCTTCGACGGCGTGCAGTTGCACGGCGCGAACGGCTATCTGATCGACCAGTTCCTGCGCGA
CGGCAGCAATCTGCGCGACGATGATTATGGCGGCCCGATCGAGAATCGCATCCGTTTGCTCCGCGAAGTC
ACAGAGGCGCTGATTTCGGTTTGGGGCGCCGACCGCGTGGGCGTCCGCCTGTCGCCCAACGGCGACACGC
AAGGTGTCGACGACAGCGCACCCGAACAGCTGTTCCCGGTCGCCGCCGCTGCGCTCGACGCGCTTGGCAT
CGCTTTCCTCGAACTGCGCGAGCCGGGCCCCGAGGGCACCTTCGGACGCACCGATGTTCCCAAACAGTCG
CCCGCGATCCGCGCGGCATTCAAAGGGCCGCTGATCCTCAACAGCGACTATGACGTCGCGAAAGCTGAAG
CTGCACTGGCCGACGGGCTCGCCGACGCGATCGCCTTTGGCCGTCCGTTCATCGGCAACCCCGACCTTGT
CGAGCGGATCCGTAACGGCGCCGAATGGGCCGCCGACAATCCGCAGACCTGGTATTCGCCGGGCCCCGAG
GGCTACACCGACTATCCGGCGCTGCAAACGGCG**CACCACCACCACCACCACTGA**

>pQR1440
MAVSLFDPIKLGAIDAPNRIIMAPLTRGRAGPGFVPTELARDYYRQRASAGLIISEATGI
SQEGLGWPSAPGLWTDAQVEGWKPVTDAVHAAGGRIVAQLWHMGRVVHSVFNDGKPPVSA
SATQAPGKAHTPVGRLDYEVARPLELGEIPRVIADYAKAAENAKRAGFDGVQLHGANGYL
IDQFLRDGSNLRDDDYGGPIENRIRLLREVTEALISVWGADRVGVRLSPNGDTQGVDDSA
PEQLFPVAAAALDALGIAFLELREPGPEGTFGRTDVPKQSPAIRAAFKGPLILNSDYDVA
KAEAALADGLADAIAFGRPFIGNPDLVERIRNGAEWAADNPQTWYSPGPEGYTDYPALQT
AHHHHHH

>pQR1442
ATGTCAGCACTGTTCAGCAACTTCAAACTCAAGGACATCACGCTCCGCAATCGCATCGCCATCCCTCCCA
TGTGCCAGTACAGTGCGGTGGATGGACTGGTGAATGACTGGCATCGCGTTCATTACGCCACACTGGCTCG
TGGCGGGGCTGGCTTGGTCATTGTGGAAGCAACCGGCGTTGCACCCGAGGGACGGATTACACCGGCTTGC
CTGGGTTTGTGGAATGATGCGCAAGCAACCGAATTAGCCAAGCTGGCAGCAGCGATCAAGGCAGGTGGTG

CCGTTCCCGGCATTCAGATTGGCCACGCTGGTCGCAAGGCCAGTGCTAATCGCCCCTGGGAGGGGGATGA
CCATATTGCCGATGGCGACCCTCGTGGATGGGCAACCCTTTCCCCATCAGCAGTGGCCTTCGGTGCCAAT
CTAGCGAAAGTCCCCAAGGCGATGACTTTTGAAGACATTGCCCGGGTGCGTGATGATTTCGTTGCTGCTG
CCAAACGGGCCCTTGACGCCGGATTCGAATGGTTGGAACTGCATTTTGCGCACGGCTATCTGGCCCAAAG
CTTCTTCTCGGTACACGCCAACCAGCGTACTGACCAATATGGTGGCGACTATGCGGGGCGTAGCCGCTTC
CTGCTGGAAACTCTCGCAGCTGTCCGCGAAGTATGGCCAGAGCATTTACCTCTGACTGCACGCTTCGGCG
TCATCGAATTCGATGGCCGTGACGAGGAAACGCTGGCGGAGTCCATCGACTTGTCTGTCGCCTTCCGTAA
GGCGGGGCTGGATATGTTGAACGTCAGCGTGGGTTTTTCCACGCTCAAGGCCAACATACCTTGGGCACCG
GCATTCCTGGCGCCAATCGCCGAGAAAGTACGCAGCGCCACCGGGATGCCGGTGGCTTCATCCTGGGGTA
TTGACATGCCGGCGACGGCTGAGCGAGTCGTGGCCGAGCATCAGATGGACTTGGTCATGATTGGCCGTGC
CCATCTGGCTAATCCTCACTGGGCTTATCAAGCAGCCAAGGTACTTGGCGTTGAAAAACCTTCCTGGGTC
TTGCCGGCGCCGTATGCTCACTGGCTTGAACGTTATAGCGCGAACGTG**CACCACCACCACCACCACTGA**

>pQR1442
MSALFSNFKLKDITLRNRIAIPPMCQYSAVDGLVNDWHRVHYATLARGGAGLVIVEATGV
APEGRITPACLGLWNDAQATELAKLAAAIKAGGAVPGIQIGHAGRKASANRPWEGDDHIA
DGDPRGWATLSPSAVAFGANLAKVPKAMTFEDIARVRDDFVAAAKRALDAGFEWLELHFA
HGYLAQSFFSVHANQRTDQYGGDYAGRSRFLLETLAAVREVWPEHLPLTARFGVIEFDGR
DEETLAESIDLSVAFRKAGLDMLNVSVGFSTLKANIPWAPAFLAPIAEKVRSATGMPVAS
SWGIDMPATAERVVAEHQMDLVMIGRAHLANPHWAYQAAKVLGVEKPSWVLPAPYAHWLE
RYSANVHHHHHH

>pQR1443
ATGACCACCGCGCCGCTCGACGATCTGGCCGCCCTGCTCGCGCCGCTCCATGCCCCCTTCACCTGCAAGT
CGCTAAAGGCGCCGAACCGCTTCTGCATGGCGCCGATGTCGCGCTATTTTGCGCCCGGCGGCGTGCTGAG
CGACGAAGGCGCCGAATATTACCGCCGCCGCGCCGCTGCGGGGATCGGCACGATCATTACCGAAGGCACC
GGCGTCGCCATCGACCATACTGTGGCGGCCGACACAGTGCCGATCTTCGCTGGCGATACCCCGCTCGCCG
CATGGAAAGGCGCCGTCGATGCCGTCCATGCCGAGGGCGGCATGTTCGTCCCCCAATTGTGGCATGTCGG
CGGCTGCATCGACTTCAACTTTCCCGACTCCCCGCATGCAGAGCTGGTTAGCCCGTCGGGCTTTGCCGGG
CCCGATGTGCCGGGCGGCCGCGCGATGACCGAACGCGATATCGCCGACACCGCCGCCGCCTTTGCAGAAT
CGGCGCGCGCCGCAAAAGAAATCGGCTGCGACGCGATCGAGCTTCACGGCGCCCATGGCTATATTTTCGA
CCAATTCTTCTGGGACCGGACCAATTTTCGCGACGATCGCTACGGCGGGCCTGACATCGGCGACCGCGCG
ACCTTCGCCGCCGAGGTCGTCGCCGCTTGCCGCGAAGCCGTGGGCGAGGATTTCGCGATCATCATGCGCG
TCTCGCAGTGGAAGACCTATGACTATGACGTGAAACTGGCGCGCGACCCCGACGAGATGCATCGCTGGCT
GGATCCGATGGTCCGCGCCGGCGTCGATATTTTCCACGCCTCGCAGCGCCGCTTCTGGGAACCCGAATAT
GCGGGCGATCCCAAGAACCTCGGCGGCTGGATCAAGGAGGTCACTGGCAAGCCGGTCATCACCGTCGGAT
CGATCGGCATGGACCGCGACCTGATGCAGGATTTCGTCGAGGGCATTTCATCACCGATGCTCGGCCGCCT
CGAGGATCTGGTGGCGATGTTCGATCGCCGCGAATTCGATCTCGTCGCGCTCGGCCGCGTGCTCCTCGCC
GACCCCAACTGGTTGGAGAAGGTCGAACAGCGCCGCATCGACGAGCTGACCGCCTACGACCGCGCGACCG
CGCTCAAACATTATGAGCATGGA**CACCACCACCACCACCACTGA**

>pQR1443
MTTAPLDDLAALLAPLHAPFTCKSLKAPNRFCMAPMSRYFAPGGVLSDEGAEYYRRRAAA
GIGTIITEGTGVAIDHTVAADTVPIFAGDTPLAAWKGAVDAVHAEGGMFVPQLWHVGGCI
DFNFPDSPHAELVSPSGFAGPDVPGGRAMTERDIADTAAAFAESARAAKEIGCDAIELHG
AHGYIFDQFFWDRTNFRDDRYGGPDIGDRATFAAEVVAACREAVGEDFAIIMRVSQWKTY
DYDVKLARDPDEMHRWLDPMVRAGVDIFHASQRRFWEPEYAGDPKNLGGWIKEVTGKPVI
TVGSIGMDRDLMQDFVEGISSPMLGRLEDLVAMFDRREFDLVALGRVLLADPNWLEKVEQ
RRIDELTAYDRATALKHYEHGHHHHHH

>pQR1445
ATGTCCAATTTATTCACCCCCCTACAGGTCGGCGCGTGGCAGTTGCCCAATCGGATCATCATGGCACCCC
TCACCCGTTGCCGTGCCAGCGAAGGCCGCGTGCCCAATGCGCTGATGGCAGAATACTACGCGCAGCGTGC
CAGTGCCGGTTTGATCATCAGTGAAGCCACCTCAGTTACACCAATGGGCGTGGGATATCCCAACACACCG
GGTATTTGGTCGGATGCGCAAGTGGACGGCTGGAAGCTGATCACCGATGCGGTGCATCAAGCCGGTGGGC
GGATTGTGCTGCAACTGTGGCATGTGGGGCGGATTTCTGATCCGGTGTATTTGGATGGTCAATTGCCGGT
TGCCCCTAGCGCGATTGCCCCACAAGGCCATGTCAGTTTGGTACGCCCGACCAAAGCGTTTGAAACCCCA
CGTGCACTCGACACCGCAGAGCTAGTCGACGTGGTCGCTGCCTATCGTTTGGGTGCAGAGAATGCGAAAA
AGGCTGGTTTTGATGGCGTTGAGATTCATGGAGCCAATGGCTATTTGCTCGATCAATTCTTGCAAAGCTC
AACCAATCAACGCACCGATCAATATGGCGGCAGCTTAGAGAACCGTGCACGCTTGCTGCTTGAAGTGGTC
GATGCTTGTATTGAGGTATGGGGTGCAGATCGGGTGGGGGTGCATCTGGCGCCGCGTGGCGATGCGCATG
ATATGGGTGATGCCAACCCTGCCGAAACCTTTGGCTATGTGGCGGAGCAATTGGGTGCGCGCCAAGTGGC
GTTTATTTGTACCCGTGAATATCTGGCCGACGACAGCCTTGCCGGTTTGATCAAAGCCAAATTTGGCGGC
GTGTATATCGCCAATGAAAAATACGACCAAGCCCAAGCCGATGCAGCCATTGCCAGCGGCGCTGCCGATG
CGGTGGCGTTTGGGGTGAAGTTTATTGCCAACCCCGATTTGCCTGCGCGTTTTGCTCAAGGTGCAGTGCT
GAATGCACCTGATCCGAGCACCTTTTATGGGGCAGGCAGTAAAGGCTATACCGACTATCTGACCTTG**CACCACCACC**
ACCACCACTGA

>pQR1445
MSNLFTPLQVGAWQLPNRIIMAPLTRCRASEGRVPNALMAEYYAQRASAGLIISEATSVT
PMGVGYPNTPGIWSDAQVDGWKLITDAVHQAGGRIVLQLWHVGRISDPVYLDGQLPVAPS
AIAPQGHVSLVRPTKAFETPRALDTAELVDVVAAYRLGAENAKKAGFDGVEIHGANGYLL
DQFLQSSTNQRTDQYGGSLENRARLLLEVVDACIEVWGADRVGVHLAPRGDAHDMGDANP
AETFGYVAEQLGARQVAFICTREYLADDSLAGLIKAKFGGVYIANEKYDQAQADAAIASG
AADAVAFGVKFIANPDLPARFAQGAVLNAPDPSTFYGAGSKGYTDYLTLHHHHHH

>pQR1446
ATGACCGCACCTGTTCAAGCCCTGTTCGCGCCCTTCCGCCTGGGTAACCTGGAGCTGCCCACCCGCGTGG
TGATGGCGCCGATGACCCGTTCCTTCTCGCCGGGCGGCGTGCCCAATGCCCAGGTGGTCGAGTACTACCG
CCGCCGTGCCGCCGCCGGCGTCGGCCTGATCGTCACCGAGGGCACCACCGTTGGCCACAAGGCCGCCAAC
GGCTATCCGCACGTGCCGCGCTTCTACGGCGAGGATGCCCTGGCCGGCTGGAAGCAGGTGGTCGATGCCG
TGCACGCCGAAGGCGGCAAGATCGTCCCGCAGCTGTGGCACGTGGGTAACGTGCGCAAGGCCGGCACCGA
GCCGGATGCCAGCGTGCCGGGTTACGGCCCGTCGGAGAAGGTCAAGGACGGCACCGTGGTCGTGCATGGC
ATGACCAAGGACGACATCCAGGAGGTGATCGCCGCCTTCGCCCAGGCCGCCCGCGACGCCAAGGCCATCG
GCATGGACGGCGTGGAGATCCACGGCGCCCACGGCTACCTGATCGACCAGTTCTTCTGGGAAGGCAGCAA
CAAGCGCACCGACGAGTACGGCGGCGACCTGGCCCAGCGCTCGCGCTTCGCCATTGAGCTGATCCAGGCC
GTGCGCGCCGCCGTTGGCCCGGACTTCCCGATCATCTTCCGCTTCTCCCAGTGGAAGCAGCAGGACTACA
CCGCACGCCTGGTACAGACCCCGGAAGAACTGGGGGCCTTCCTCAAGCCGCTGTCCGACGCCGGCGTGGA
TATCTTCCACTGCTCGACCCGCCGCTTCTGGGAGCCGGAGTTCGAAGGCAGCGACCTCAACCTGGCCGGC
TGGACCCGCCAGCTCACCGGCAAGCCGACCATCACCGTCGGCAGCGTCGGCCTGGACGGCGAGTTCCTGC
AGTTCATGGTCAACACCGACAAGGTGGCCGAGCCGGCCAGCCTGGAGAACCTGCTGGAGCGCCTGAACAA
GCAGGAGTTCGACCTGGTGGCCGTGGGCCGTGCCCTGCTGGTCGACCCGGACTGGGCGGTGAAGGTGCGC
GATGGCCGCGAGAGCGACATCCTGCCGTTCAGCCGCGAGGCGCTCAAGCAGCTGGTC**CACCACCACCACCACCAC
TGA**

>pQR1446
MTAPVQALFAPFRLGNLELPTRVVMAPMTRSFSPGGVPNAQVVEYYRRRAAAGVGLIVTE
GTTVGHKAANGYPHVPRFYGEDALAGWKQVVDAVHAEGGKIVPQLWHVGNVRKAGTEPDA
SVPGYGPSEKVKDGTVVVHGMTKDDIQEVIAAFAQAARDAKAIGMDGVEIHGAHGYLIDQ
FFWEGSNKRTDEYGGDLAQRSRFAIELIQAVRAAVGPDFPIIFRFSQWKQQDYTARLVQT
PEELGAFLKPLSDAGVDIFHCSTRRFWEPEFEGSDLNLAGWTRQLTGKPTITVGSVGLDG
EFLQFMVNTDKVAEPASLENLLERLNKQEFDLVAVGRALLVDPDWAVKVRDGRESDILPF
SREALKQLVHHHHHH

>pQR1907
ATGTCCGGCAAGTTGTTCACCCCGTTCAGCTCGGGTTCCTTCACCTTCCCCAACCGCGTTATCATGGCGC
CGCTGACGCGTATGCGCGCTTCGCAGCCGGGTGACATTCCCAACGAGCTGATGCAGACCTATTACGTGCA
GCGCGCCAGCGCCGGCCTCATCATCGCCGAGGCCACGCAGATCTCCCCGCAGGGCAAGGGCTATATGGAC
ACTCCGGGGATTTATTCCGCGGAGCAGGTGCAGGGCTGGCGCAAGATCACCCAGGCCGTGCATGAGGCCG
GTGGCCATATCGCCCTGCAGCTCTGGCATGTGGGTCGTGTTTCGCATCACAGCCTGCAGCCCGACCAGCA
ACTGCCGGTGTCCGCTTCTGCCATTCCCTACCAGAACCGCACCACGGTCCGTGGTGAAGACGGCAAGCCC
ACGCGCGTGGATTGCGATACCCCACGTGCGCTGGAACTGTCCGAAATCCCCGGTGTGATCGAAGACTACC
GCCGCGCCACCGTGAATTCGCGCGAAGCCGGTTTCGACATGGTGGAAGTGCATGCCGCGCATGGCTATCT
GCTGCACCAGTTCCAGTCCGCCGAAAGCAACAAGCGTGAAGACGCCTATGGTGGTTCGCTGGAAAACCGT
GCCCGCCTGACGCTGGAAGCCCTGGATGCCGTGATCGGTGCCTGGGATGCCAAGCATGTAGGTATCCGCA
TTTCCCCGCTGGGCACCTTCAACGGCCTGGACGACAAGGACGGCCTGGAAATGGCGCTGTATCTCACGCG
TGAATTCACCAAGCGCGGTATCGCCTACCTGCATCTGTCCGAGCCGGACTGGGCCGGCGGTCCGGCGCAT
GGCGACGAATTCCGCCAGGCCCTGCGCGACGCTTTCCCGGGCACCATCATCGGTGCCGGCAACTACACGG
TGGAAAAATCGGAGATGCTGCTGGCCAAGGGCTTTATCGATGCCGCCGCGTTTGGTCGTCCCTTTATTGC
CAATCCGGACCTGCCGGTGCGTCTGCAGAAGGGCGCTGAGTTGAACAATGTGGTGGCGGCTACGCTGTAT
GGCGGTGGCGCCGAAGGCTATACGGATTATCCGGCGCTGGCC**CACCACCACCACCACCACTGA**

>pQR1907
MSGKLFTPFSSGSFTFPNRVIMAPLTRMRASQPGDIPNELMQTYYVQRASAGLIIAEATQ
ISPQGKGYMDTPGIYSAEQVQGWRKITQAVHEAGGHIALQLWHVGRVSHHSLQPDQQLPV
SASAIPYQNRTTVRGEDGKPTRVDCDTPRALELSEIPGVIEDYRRATVNSREAGFDMVEV
HAAHGYLLHQFQSAESNKREDAYGGSLENRARLTLEALDAVIGAWDAKHVGIRISPLGTF
NGLDDKDGLEMALYLTREFTKRGIAYLHLSEPDWAGGPAHGDEFRQALRDAFPGTIIGAG
NYTVEKSEMLLAKGFIDAAAFGRPFIANPDLPVRLQKGAELNNVVAATLYGGGAEGYTDY
PALAHHHHHH

>pQR1908
ATGCACTCCTCACTCTTCAATCCCTTGCAAGTTGGCGCTCTCACGGTTCCTAACCGCATCCTGCTGGCGC
CTTTGACCCGCGCGCGCGCAGATGCTGGCCATATGCCCAACGCCCTCATGGCCGAGTACTACGCCCAGCG

CGCCAGCGGTGGCCTGCTGATCACTGAGTGCACCATGGTGGCCCCCAACACCTCGGCCTTCATCGCAGAG
CCGGGTATTTACTCGCCAGAGCAGGTGGCCGCCTGGAAGCAGGTCACCTCCGCCGTGCACGCCAAGGGCG
GGCGCATCTACATGCAAATCTGGCACGCGGGCCGCGCTGCGCACCCGGCCATCAACGGCGGCACGCCCAC
CGTGTCGTCCACCGCAACCCCCATCGAGGGCGACATCCACACCCCCACCGGCAAAGTTCCGCATGTGCCC
GCGCATGTGCTTACCGAGTCTGAAATCCCCGGCATCGTCGAAGCCTTCGTGCAGGGCGCCAAAAACGCCA
TCGCCGCCGGCTTTGATGGCGTGGAAGTGCACGCAGCCAACGGCTACCTCATCGACCAGTTCCTGCGCGA
CGGCGCGAACGACCGCACCGACGGCTACGGCGGCCCGTTGGAGAACCGCGCGCGCTTCCTGTTTGAAGTG
CTGACTGCCGTGACCGCCGCCATCGGCAGCGACCGCGTGGGCGTGCGCTTGTCGCCGCTGAACAGCTTCA
ACAGCATGAAAGACAGCGATCCCATCGCGTTCATCGGCTTCCTGGCCGAAAAGCTCAACGCCTTCAAGCC
GGCCTATCTGCATGTGATGCGGGCCGATTTTCTGCAGGCGCAGCATGGCGACGTGCTCTCGGTGGCGCGC
GCCAAGTACCACGGCGTACTGGTTGGAAATATGGGCTATAGCGCAGAAGAAGCCGAGCAGGCGATTGCCG
GCGGCAAGCTCGACGCCGTGGCCTTTGGCACCAGCTTTTTGGCCAATCCCGATCTGCCAGAACGCATCCA
GGCCGGTGCCGCGCTGAACGCACCTAACCCCAACACGTTCTACTCGCCCGGCCCCGTGGGCTACACCGAC
TACCCCACAATGGCCGGT**CACCACCACCACCACCACTGA**

>pQR1908
MHSSLFNPLQVGALTVPNRILLAPLTRARADAGHMPNALMAEYYAQRASGGLLITECTMV
APNTSAFIAEPGIYSPEQVAAWKQVTSAVHAKGGRIYMQIWHAGRAAHPAINGGTPTVSS
TATPIEGDIHTPTGKVPHVPAHVLTESEIPGIVEAFVQGAKNAIAAGFDGVEVHAANGYL
IDQFLRDGANDRTDGYGGPLENRARFLFEVLTAVTAAIGSDRVGVRLSPLNSFNSMKDSD
PIAFIGFLAEKLNAFKPAYLHVMRADFLQAQHGDVLSVARAKYHGVLVGNMGYSAEEAEQ
AIAGGKLDAVAFGTSFLANPDLPERIQAGAALNAPNPNTFYSPGPVGYTDYPTMAGHHHH
HH

>pQR1909
ATGAGTCGCCTGTTCTCGCCCCTCGCGCTGGGGCCGTTGCCGTTGTCCAACCGCATCGTCATCGCGCCCA
TGTGCCAGTACTCCGCCGACGAAGGTCGTGCGACCGACTGGCACGCCTTCCACTGGCCGAACCTGGCCCA
GTCCGGTGCTGCGCTGGCGATCATCGAAGCCACCGCGGTCGAGCCGCGCGGGCGCATCAGCTGGGCGGAC
CTTGGCCTGTGGGACGACACGACGGAAGCCGCCTTCGCCCGCGCGCTGGCGGCGGCACGCAGGTACTCGA
CGATGCCGATCGGCGTGCAGCTGGCGCATGCGGGACGCAAGGCATCCACGCACCGCCCGTGGGAGCATCA
CGGCGCACAGATAGCACCCGACGCGCCGCAGGGATGGCGGACGGTATCGGCGTCGTCGTTGCCCTATGCC
GAGGGGCAGCATCCGCCGGACTCGTTGGACCAGGCGGGCATCGACGCGATCATCGCCGCCTTCGCCGACA
GCGCGCATCGCGCTGTGCGACTCGGGCTGGACCTGATCGAGATCCATGCCGCCCACGGCTACCTGCTGCA
CCAGTTCCTGTCGCCGCTGAGCAACCGGCGCGACGACGCCTACGGCGGCGCGCTGGAAAATCGCATGCGC
CTGGTGTTGCAGGTGTTCGATGCGATCAAGGCCGTGGTGCCGGCGTCGATGGCGGTCGGGGTACGCATTT
CCGCGACCGACTGGGTCGACGGCGGCTGGGATCTGGCGCAGAGCATCGCGCTGGCGAAGGCGCTGGATGC
GCGTGGCAGCCACTTCATCCATGTGTCGAGCGGCGGCCTGCATCCGGCGCAGAAGATCGCGCTGGGGCCC
GGCTACCAGGTGCCCTTCGCAGCGGCGATCAAGCGTGAAGTCGCGATGCCGGTGATCGCGGTCGGCCTGA
TCACGGCGCCGCAACAGGCGGAGGACATCCTGCTGCAGGCGCAGGCCGATGCCATCGCCATCGCGCGCGC
CGTTCTCTACGATCCTCGTTGGCCGTGGCACGCGGCGGCATCGCTTGGCGAGACGATCGCCATCGCGCCG
CAGTACCTGCGCAGCGCACCGCGGGAGGTCGCGGCGAGCTTCGTGGAGGCGACG**CACCACCACCACCACCACTG**
A

>pQR1909
MSRLFSPLALGPLPLSNRIVIAPMCQYSADEGRATDWHAFHWPNLAQSGAALAIIEATAV
EPRGRISWADLGLWDDTTEAAFARALAAARRYSTMPIGVQLAHAGRKASTHRPWEHHGAQ
IAPDAPQGWRTVSASSLPYAEGQHPPDSLDQAGIDAIIAAFADSAHRAVRLGLDLIEIHA
AHGYLLHQFLSPLSNRRDDAYGGALENRMRLVLQVFDAIKAVVPASMAVGVRISATDWVD
GGWDLAQSIALAKALDARGSHFIHVSSGGLHPAQKIALGPGYQVPFAAAIKREVAMPVIA
VGLITAPQQAEDILLQAQADAIAIARAVLYDPRWPWHAAASLGETIAIAPQYLRSAPREV
AASFVEATHHHHHH

BIOCATALYSIS

General considerations

All chemicals were obtained from commercial suppliers and used as received. Thin layer chromatography was carried out using Merck TLC Silica gel 60 F_{254} plates and products were visualised using combinations of UV light (254 nm) and potassium permanganate staining solution. Filtrations on silica were carried out using silica gel (particle size 40–60 µm). GC analyses were performed using an Agilent 7820A Gas Chromatograph equipped with a FID detector, and a chiral column (Beta DEX 225, fused silica capillary column 30 m x 0.25 mm x 0.25 µm). HPLC analyses of the reactions were performed using an Agilent 1260 Infinity HPLC with an OJ chiralcel column (250 x 4.6 mm) or an OB chiralcel column (250 x 4.6 mm). Spectrophotometric assays were performed using Tecan GENios Microplate Reader.

^{1}H NMR spectra were recorded at 600 MHz, on a Bruker Avance 600 spectrometer using the residual protic solvent stated as the internal standard. Chemical shifts are quoted in ppm to the nearest 0.01 ppm using the following abbreviations: s (singlet), d (doublet), t (triplet), q (quartet), qn (quintet), sext (sextet), dd (doublet of doublets), dt (doublet of triplets), m (multiplet) defined as all multi-peak signals where overlap or complex coupling of signals make definitive descriptions of peaks difficult. ^{13}C{^{1}H} NMR spectra were recorded at 125, 150 or 175 MHz on a Bruker Avance 500, 600 and 700 MHz spectrometers at 25 °C using the stated solvent as standard. Chemical shifts are reported to the nearest 0.1 ppm. The coupling constants are defined as *J* and quoted in Hz. Mass spectra were performed in the Department of Chemistry (University College London). Infrared spectra were obtained as thin film on a Perkin Elmer Spectrum 100 FT-IR Spectrometer operating in ATR mode. Melting points were measured with a Gallenkamp apparatus and were uncorrected. All optical rotations were measured on a Perkin-Elmer 343 polarimeter with a path length of 1 dm.

Spectrophotometric assay

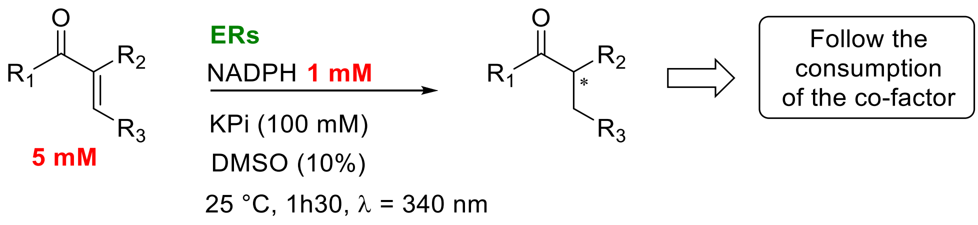

ERs spectrophotometric assays were performed with purified enzyme and in 96-well plates. The assays were initiated by the addition of NADPH (160 µL, 1.25 mM) dissolved in phosphate buffer (100 mM, pH = 8) to a mixture containing the substrate (10 µL, 100 mM in DMSO), water (25 µL) and the purified enzyme (5 µL in phosphate buffer). Depletion of NADPH was monitored for 1h 30 min using a UV-vis spectrometer at λ = 340 nm. In **Table S2** the qualitative results of these assays are reported.

Table S2. Qualitative results of the spectrophotometric assay.

	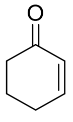	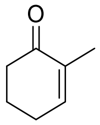
**pQR1439**	-	-
**pQR1440**	+	+
**pQR1442**	+	+
**pQR1443**	*n.t.*	*n.t.*
**pQR1445**	+	+
**pQR1446**	+	+
**pQR1907**	+	+
**pQR1908**	+	+
**pQR1909**	+	+

+ = activity; - = no activity; *n.t.* = not tested.

Biotransformations - general protocol with purified ERs

Purified enzymes were prepared from ammonium sulfate suspension by sampling the volume of enzyme needed into an Eppendorf tube. After centrifugation for 20 min at 4 °C at 13 000 rpm, the supernatant was discarded, and the residual pellet was dissolved in the desired volume of Tris buffer (50 mM) to reach a concentration between of 2.0 to 8.0 mg/mL depending on the enzyme. Concentrations were measured using a NanoDrop UV-Vis spectrophotometer.

Reactions using commercial G6PDH (Aldrich, *Leuconostoc mesenteroides* lyophilized powder, 550-1,100 units/mg) were performed as follows. The bioconversion was started by the addition of 25 µL of purified enzyme (2.0 - 8.0 mg/mL depending the enzyme) dissolved in Tris buffer (50 mM, pH = 7.4) to a mixture of $NADP^+$ (140 µL, 5 mM), glucose-6-phosphate sodium salt (25 µL, 1 M), G6PDH (35 µL, 20 U, 1 mg/mL) and the substrate stock solution in DMSO (25 µL, 100 mM), giving final concentrations between 0.2 - 0.8 mg/mL of enzymes, 10 mM of substrate, 2.8 mM of $NADP^+$, 100 mM of glucose-6-phosphate sodium salt and 10% of DMSO. The mixture was shaken at 30 °C and 300 rpm for 20 h or 3 h 30 min depending on the substrate used. The reaction was then quenched by addition of TFA (10% in H_2O, 10 µL) and centrifuged for 10 minutes at 4 °C at 13 000 rpm. The clear solution was placed in a new Eppendorf tube, EtOAc (250 µL) was added and the resulting biphasic solution was mixed using a vortex shaker and centrifuged for 20 minutes at 13 000 rpm. The organic layer (125 µL) was sampled into an Eppendorf tube and dried with Na_2SO_4. If the sample was analysed by GC, the dried organic phase (75 µL) was placed in a vial containing an insert and injected into the GC. In the case where the sample was analysed by HPLC, the dried organic phase (75 µL) was poured into an Eppendorf tube and the solvents were let to evaporate overnight before dissolution

in *i*PrOH (75 µL). After mixing, the mixture was transferred in a vial containing an insert and injected into HPLC. Each biotransformation was performed in triplicate.

Figure S3. Bioreduction of carvone **5**: 20 h reaction time.

Biotransformations were carried out following the general protocol with purified ERs. Quantification of the product was performed using GC analysis (retention time and calibration curves are reported the Analytical methods section)**.** Without ERs, no background reaction was observed.

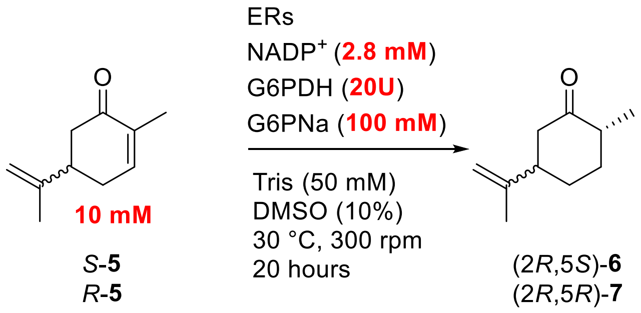

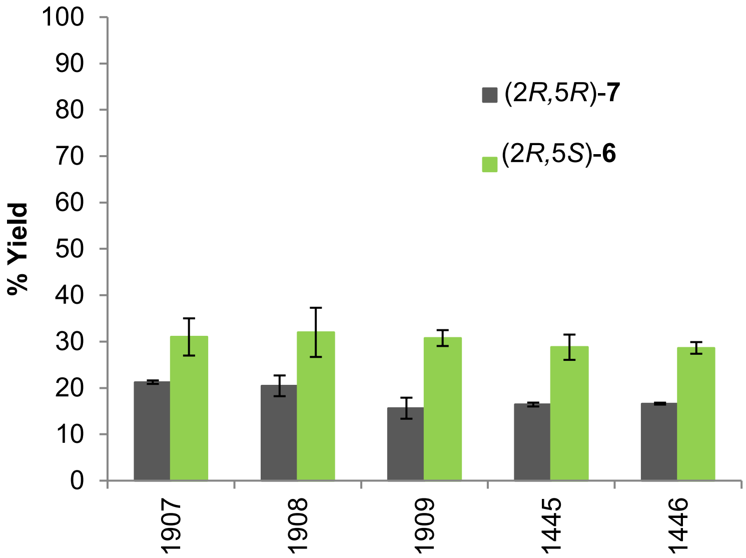

Figure S4. Bioreduction of carvone **5**: 3.5 h reaction time.

Reaction conditions: Substrate (10 mM), purified ER (0.2-0.9 mg/mL), NADP+ (2.8 mM), G6PDH (20 U), G6PNa (100 mM), in Tris-HCl (50 mM) and DMSO (10%) at pH 7.4, 30 °C, 300 rpm, 3.5 hours. Reactions were performed in triplicate. Yields were determined by GC analysis.

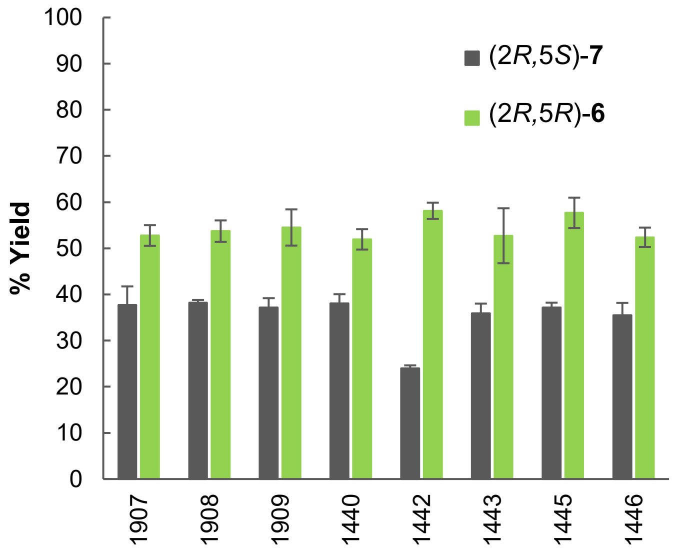

Figure S5. Time-course experiment for the bioreduction of *S*-**5.**

Reaction conditions: Substrate (10 mM), purified ER (0.2-0.9 mg/mL), NADP+ (2.8 mM), G6PDH (20 U), G6PNa (100 mM), in Tris-HCl (50 mM) and DMSO (10%) at pH 7.4, 30 °C, 300 rpm. Reactions were performed in triplicate. Yields were determined by GC analysis.

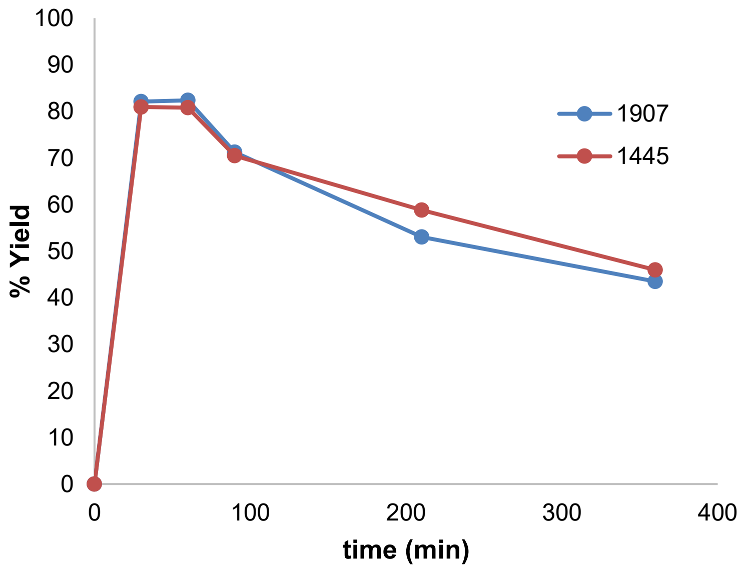

Figure S6. Conversion profile for bioreduction of *rac*-**8** with pQR1907.

Biotransformations were carried out following the procedure described. Conversions were measured using HPLC (retention time and calibration curves are reported in the Analytical methods section).
Substrate (10 mM), purified ER (0.2 - 0.9 mg/mL), $NADP^+$ (2.8 mM), G6PDH (20 U), G6PNa (100 mM), in Tris-HCl (50 mM) and DMSO (10%) at pH = 7.4, 30 °C, 20 h, 300 rpm. Reactions were performed in triplicate. Conversions were determined by HPLC based on the depletion of starting material.

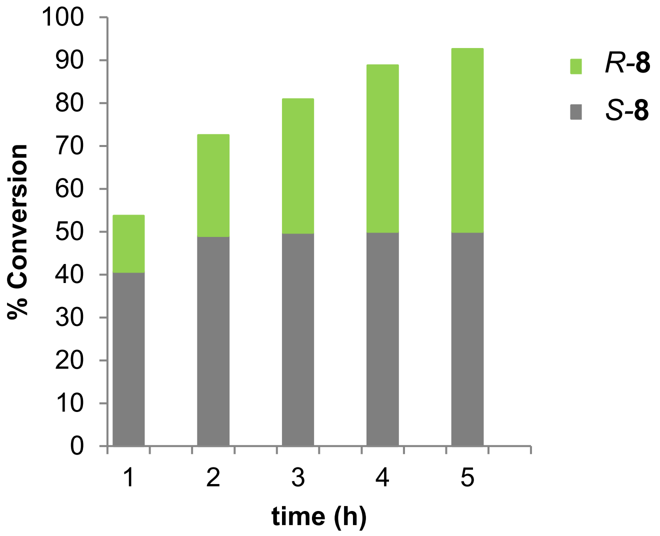

Biotransformations with clarified cell lysates

The bioconversion was started with the addition of 60 µL of co-expressed ene-reductase/G6PDH lysate (2 -4 mg/mL, total cell protein dissolved in Tris buffer (50 mM, pH = 7.4) to a mixture of $NADP^+$ (140 µL, 5 mM), glucose-6-phosphate sodium salt (25 µL, 1 M), and the substrate stock solution in DMSO (25 µL, 100 mM) giving final concentrations between 0.5 to 1 mg/mL of enzymes, 10 mM of substrate, 2.8 mM of $NADP^+$, 100 mM of glucose-6-phosphate sodium salt and 10% of DMSO. The mixture was shaken at 30 °C and 300 rpm for 20 h. The reaction was then quenched by the addition of TFA (10% in H_2O, 10 µL) and centrifuged for 10 minutes at 4 °C at 13 000 rpm. The clear solution was placed in a new Eppendorf tube, EtOAc (250 µL) was added and the resulting biphasic solution was mixed using a vortex shaker and centrifuged for 20 min at 13 000 rpm. The organic layer (125 µL) was sampled into an Eppendorf tube and dried with Na_2SO_4. If the sample was analysed by GC, the dried organic phase (75 µL) was placed in a vial containing an insert and injected into the GC. For HPLC analysis, the dried organic phase (75 µL) was poured into an Eppendorf tube and the solvent were left to evaporate overnight before dissolution in EtOH (75 µL). After mixing, the mixture was transferred in a vial containing an insert and injected into the HPLC. All biotransformations were performed in triplicate.

Figure S7. Bioreduction of *S*-**5**.

Quantification of the product was performed using GC analysis (retention time and calibration curves are reported in the Analytical methods section).

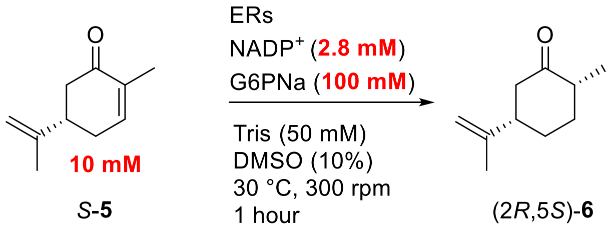

	Method	Enzyme [mg/mL]	Substrate [mM]	% Yield	% *ee*
**1907/G6PDH**	A	0,5	10	96	95
	B	0,5	20	68	96
	C	1	10	99	93
	D	1	20	65	96
**1445/G6PDH**	A	0,5	10	95	97
	B	0,5	20	61	97
	C	1	10	90	96
	D	1	20	59	97

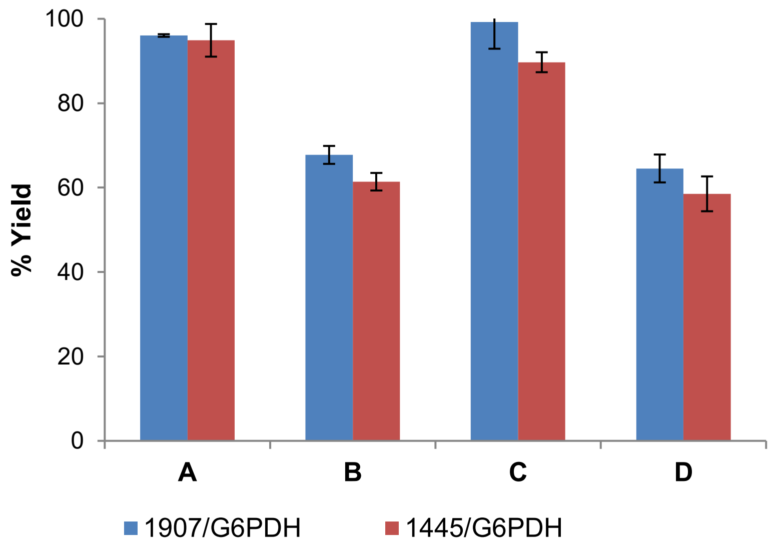

With *S*-**5** the highest yields and stereoselectivities were observed with the ER from pQR1445 (0.5 mg/mL) and 10 mM substrate. These conditions were chosen for the preparative scale.

Figure S8. Bioreduction of *S*-**8**.

Conversions were measured using HPLC (retention time and calibration curves are reported in the Analytical methods section).

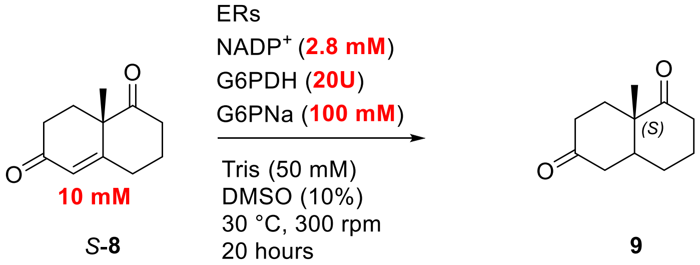

	Method	Enzyme [mg/mL]	Substrate [mM]	% Conversion
**1907/G6PDH**	**A**	0,5	10	100
	**B**	0,5	20	100
	**C**	1	10	100
	**D**	1	20	100
**1445/G6PDH**	**A**	0,5	10	95
	**B**	0,5	20	71
	**C**	1	10	100
	**D**	1	20	99

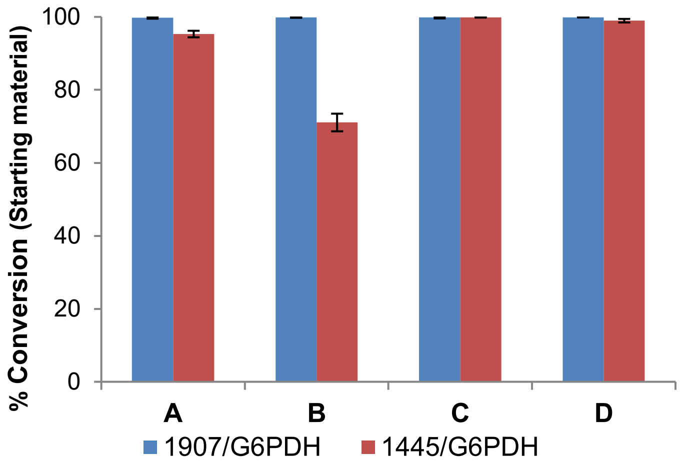

In comparison to *S*-**5**, *S*-**8** could be used at higher concentrations (20 mM) but the best yields were observed with the ER from pQR1907 (1 mg/mL).

MOLECULAR DOCKING

Homology model of pQR1907 was constructed by the Phyre^2 webserver.[11] The quality of the prediction was additionally checked with Qred parameters.[12–14] Ligands were MMFF94 energy minimised in Avogadro. Docking was performed with Chimera UCSF using the AutoDock Vina plug-in,[15] (docking box parameters: position (10, 1, 20) and size (50, 50, 50) and settings: energy-range 3, exhaustiveness 8, number of modes 9). Binding modes relevant to the explanation of enzyme reactivity were selected.

Table S3. Computational docking output (Autodock Vina) – pQR1907 model with ligands (FMN and *S*-**8**)

**Rank**	**Binding affinity (kcal/mol)**	
	**FMN**	***S*-8**
1	-10.7	-6.3
2	-10.5 best	-6.2
3	-9.9	-6.1
4	-9.2	-5.9 best
5	-9.0	-5.7
6	-8.8	-5.7
7	-8.8	-5.7
8	-8.6	-5.6
9	-8.6	-5.6

PREPARATIVE SCALE BIOTRANSFORMATIONS

Reactions on a 25 mL scale

Synthesis of (2*R*,5*S*)-2-methyl-5-(prop-1-en-2-yl)cyclohexan-1-one (6)

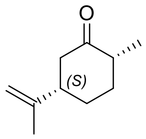

In a falcon tube (50 mL), the co-expressed 1445/G6PDH (6 mL, 2.2 mg/mL of total cell protein in Tris.HCl (50 mM, pH = 7.4)) was added to a mixture of NADP$^+$ (14 mL, 5 mM), glucose-6-phosphate sodium salt (2.5 mL, 1 M) and substrate (2.5 mL, 100 mM in DMSO) giving final concentrations of 0.5 mg/mL of enzymes, 10 mM of substrate, 2.8 mM of NADP$^+$, 100 mM of glucose-6-phosphate sodium salt and 10% of DMSO. The solution was incubated for 1 h at 30 °C and an aliquot was taken to quantify the conversion by GC (see chromatogram below, quantitative, *d.r.* = 96:4). Then an aqueous solution of TFA (5%, 1 mL) was added. The resulting mixture was extracted with EtOAc (3 x 20 mL). The combined organic layers were washed with water

(1 x 20 mL) and brine (1 x 20 mL) and dried over Na_2SO_4. After filtration and evaporation to dryness the colourless oil obtained was dissolved in Et_2O and filtered through a small pad of silica. After evaporation the desired compound was obtained as a colourless oil (27 mg, 95%, *d.r.* 98:2 (NMR)); $[\alpha]_D^{20}$ −12.0 (c 0.7, EtOH), lit $[\alpha]_D^{22}$ −16.9 (c 1.2, $CHCl_3$),[16] ^1H NMR (600 MHz, $CDCl_3$) δ = 4.84 (br s, 1H, CCH*H*), 4.69 (br s, 1H, CC*H*H), 2.61 (m, 1H, =CC*H*), 2.56 (dd, *J* = 14.2, 6.7, 1H, CH*H*(C=O)), 2.36 - 2.55 (m, 2H, CH*H*(C=O), C*H*(C=O)), 1.80 - 1.92 (m, 3H, (C=O)CHC*H*H, =CCHCH_2), 1.73 (s, 3H, CH_3(C=C)), 1.58 - 1.65 (m, 1H, (C=O)CHC*H*H), 1.09 (d, *J* = 6.9, 3H, CHCH_3) ppm; $^{13}C\{^1H\}$ NMR (151 MHz, $CDCl_3$) δ =214.0 (*C*=O), 147.1 (=*C*), 111.7 (=*C*H_2), 44.7 (*C*H), 44.2 (*C*H_2), 44.1 (*C*H), 30.7 (*C*H_2), 26.3 (*C*H_2), 21.7 (*C*H_3), 15.7 (*C*H_3) ppm; FT-IR (ATR) υ_{max} = 2959, 2924, 2855, 1706, 1642 cm^{-1}; HRMS (EI) calcd for $C_{10}H_{16}O$ $[M]^+$ 152.1196 found 152.1196.

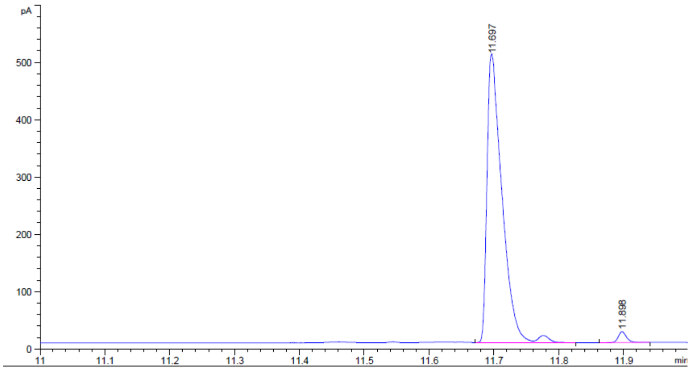

Peak #	RetTime [min]	Type	Width [min]	Area [pA*s]	Height [pA]	Area %
1	11.697	BV R	0.0235	813.37421	499.69772	98.00624
2	11.898	BB	0.0143	16.54667	18.80960	1.99376
Totals :				829.92088	518.50732	

Synthesis of (4a*R*,8a*S*)-8a-methylhexahydronaphthalene-1,6(2*H*,5*H*)-dione (9)

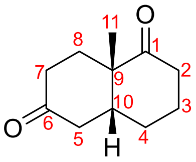

In a falcon tube (50 mL), the co-expressed 1907/G6PDH (6 mL, ≈ 4.0 mg/mL of total cell protein in Tris.HCl (50 mM, pH = 7.4) was added to a mixture of $NADP^+$ (14 mL, 5 mM), glucose-6-phosphate sodium salt (2.5 mL, 1 M) and substrate (2.5 mL, 200 mM in DMSO) giving final concentrations of 1.0 mg/mL of enzymes, 20 mM of substrate, 2.8 mM of $NADP^+$, 100 mM of glucose-6-phosphate sodium salt and 10% of DMSO. The solution was incubated for 20 h at 30 °C before the addition of an aqueous solution of TFA (5%, 1 mL). The resulting mixture was extracted with EtOAc (3 x 20 mL). The combined organic layers were washed with water (1 x 20 mL) and brine (1 x 20 mL) and dried over Na_2SO_4. After filtration and evaporation to dryness the colourless oil obtained was dissolved in Et_2O and filtered through a small pad of silica. After evaporation the colourless oil crystallized to give the product as a white solid (87

mg, 95%, *syn* isomer only); mp = 49 °C (Et_2O); $[\alpha]_D^{20}$ +1 (c 1.0, EtOH), lit $[\alpha]_D^{25}$ +9 (c 1.0, C_6H_6, 75% ee)[17*] ; ^{1}H NMR (600 MHz, $CDCl_3$) δ = 2.57 (ddd, *J* = 15.1, 10.1, 6.6, 1H, $H_{2\text{-ax}}$); 2.46 - 2.54 (m, 2H, $H_{8\text{-ax}}$, $H_{7\text{-ax}}$), 2.41 (ddd, *J* = 15.1, 5.6, 1.2; 1H, $H_{2\text{-eq}}$), 2.24 - 2.36 (m, 4H, $H_{7\text{-eq}}$, $H_{5\text{-ax\&eq}}$, H_{10}), 2.10 (dddd, *J* = 14.4, 10.3, 4.5, 3.6, 1H, $H_{4\text{-ax}}$), 1.88 - 2.02 (m, 2H, $H_{3\text{-ax\&eq}}$), 1.52 (m, 1H, $H_{4\text{-eq}}$), 1.39 - 1.48 (m, 1H, $H_{8\text{-eq}}$), 1.35 (s, 3H, CH_3) ppm; ^{13}C{^{1}H} NMR (151 MHz, $CDCl_3$) δ = 214.0 (*C*=O), 211.0 (*C*=O), 48.5 (C_9), 46.2 (C_{10}), 43.9 (C_5), 38.6 (C_7), 37.4 (C_2), 33.7 (C_8), 26.9 (C_4), 24.1 (CH_3), 23.0 (C_3) ppm; FT-IR (ATR) $υ_{max}$ = 2971, 2935, 2922, 2870, 2858, 1695, cm^{-1}; HRMS (CI) calcd for $[M+NH_4]^+$ 198.1489 found 198.1488.

Synthesis of (4a*R*,5*R*,8a*S*)-5,8a-dimethylhexahydronaphthalene-1,6(2*H*,5*H*)-dione (12)

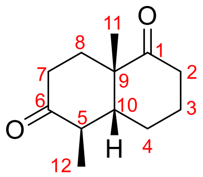

In a falcon tube (50 mL), the co-expressed 1907/G6PDH (6 mL, ≈ 8.0 mg/mL of total cell protein in Tris.HCl (50 mM, pH = 7.4) was added to a mixture of $NADP^+$ (14 mL, 5 mM), glucose-6-phosphate sodium salt (2.5 mL, 1 M) and substrate (2.5 mL, 200 mM in DMSO, 90 %ee)* giving final concentrations of 2.0 mg/mL of enzymes, 20 mM of substrate, 2.8 mM of $NADP^+$, 100 mM of glucose-6-phosphate sodium salt and 10% of DMSO. The solution was incubated for 20 h at 30 °C before the addition of an aqueous solution of TFA (5%, 1 mL). The resulting mixture was extracted with EtOAc (3 x 20 mL). The combined organic layers were washed with water (1 x 20 mL) and brine (1 x 20 mL) and dried over Na_2SO_4. After filtration and evaporation to dryness the colourless oil obtained was dissolved in Et_2O and filtered through a small pad of silica. After evaporation to dryness the crude oil was analysed by NMR and revealed a mixture of diastereomers 95:5 (90% *d.e.*). Purification by column chromatography on silica using petroleum ether (40 – 60 °C)/ether as eluent (100:0 to 60.40) gave the product as a colourless oil (75 mg, 71%, *syn* isomer only); $[\alpha]_D^{20}$ −63 (c 1.0, $CHCl_3$); ^{1}H NMR (700 MHz, $CDCl_3$) δ = 2.67 (ddd, *J* = 15.5, 13.4, 7.6, 1H, $H_{2\text{-ax}}$), 2.53 – 2.63 (m, 2H, $H_{8\text{-ax}}$, $H_{7\text{-ax}}$), 2.35 (ddt, *J* = 15.5, 5.3, 1.8, 1H, $H_{2\text{-eq}}$), 2.22 – 2.30 (m, 2H, H_5, $H_{7\text{-eq}}$), 2.14 (tt, *J* = 14.2, 4.0, 1H, $H_{4\text{-ax}}$), 1.94 - 2.01 (m, 1H, $H_{3\text{-ax}}$), 1.83 – 1.92 (m, 2H, $H_{10\text{-ax}}$, $H_{3\text{-aq}}$), 1.77 – 1.83 (m, 1H, $H_{4\text{-eq}}$), 1.31 (ddd, *J* = 13.8, 5.0, 1.9, 1H, $H_{8\text{-eq}}$), 1.28 (s, 3H, C$H_{3\text{-}11}$), 0.98 (d, *J* = 6.5, 3H, C$H_{3\text{-}12}$); ^{13}C{^{1}H} NMR (176 MHz, $CDCl_3$) δ = 214.3 (*C*=O), 213.4 (*C*=O), 52.9 (C_{10}), 49.5 (C_9), 44.0 (C_5), 38.9 (C_7), 37.7 (C_2), 35.3 (C_8), 26.7 (C_{11}), 22.8 (C_4), 21.1 (C_3), 11.4 (C_{12}). ppm; FT-IR (ATR) $υ_{max}$ = 2953, 2926, 2874, 1700 cm^{-1}; HRMS (ESI) calcd for $[M+H]^+$ 217.1199 found 217.1202.

* Substrate *S*-**5** was synthesised following the procedure described in *Org. Lett.* **2017**, *19*, 1527 and the *ee*. was determined by HPLC (Chiralcel OB, *i*PrOH:hexane (10:90), tr_1 = 52.6 min , tr_2 = 86.0 min)

Synthesis of (3a*R*,7a*S*)-7a-methylhexahydro-1H-indene-1,5(4*H*)-dione (15)

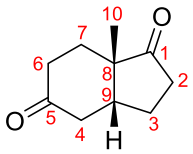

In a falcon tube (50 mL), the co-expressed 1907/G6PDH (6 mL, ≈ 8.0 mg/mL of total cell protein in Tris.HCl (50 mM, pH = 7.4) was added to a mixture of $NADP^+$ (14 mL, 5 mM), glucose-6-phosphate sodium salt (2.5 mL, 1 M) and substrate (2.5 mL, 200 mM in DMSO) giving final concentrations of 2.0 mg/mL of enzymes, 20 mM of substrate, 2.8 mM of $NADP^+$, 100 mM of glucose-6-phosphate sodium salt and 10% of DMSO. The solution was incubated for 20 h at 30 °C before the addition of an aqueous solution of TFA (5%, 1 mL). The resulting mixture extracted with EtOAc (3 x 20 mL). The combined organic layers were washed with water (1 x 20 mL) and brine (1 x 20 mL) and dried over Na_2SO_4. After filtration and evaporation to dryness the colourless oil obtained was dissolved in Et_2O and filtered through a small pad of silica. After evaporation the colourless oil crystallized to give the product as a white solid (83 mg, 94%, *syn* isomer only); mp = 58 °C (EtOAc); $[\alpha]_D^{20}$ + 105 (c 1.0, $CHCl_3$), lit $[\alpha]_D^{25}$ + 79 (c 0.28, $CHCl_3$, 86% ee)[17]; ^1H NMR (700 MHz, $CDCl_3$) δ = 2.58 (ddd, *J* = 15.1, 6.4, 1.1, 1H, $H_{4\text{-ax}}$), 2.43 (dddd, *J* = 19.3, 8.9, 4.2, 0.7, 1H, $H_{2\text{-eq}}$), 2.40 (br m, 1H, $H_{9\text{-eq}}$) 2.38 (dddd, *J* = 15.1, 10.6, 5.5, 1.1, 1H, $H_{6\text{-ax}}$), 2.32 (ddd, *J* = 19.3, 9.3, 8.4, 1H, $H_{2\text{-ax}}$), 2.28 (ddd, *J* = 15.1, 5.2, 1.6, 1H, $H_{4\text{-eq}}$), 2.22 (dddd, *J* = 15.1, 6.3, 5.0, 1.6, 1H, $H_{6\text{-eq}}$), 2.11 (dddd, *J* = 13.5, 9.3, 6.9, 4.3, 1H, $H_{3\text{-eq}}$), 2.01 (ddd, *J* = 14.1, 10.6, 4.9, 1H, $H_{7\text{-aq}}$), 1.64 (dddd, *J* = 14.2, 6.3, 5.5, 1.1, 1H, $H_{7\text{-eq}}$), 1.61 (ddt, *J* = 13.3, 8.4, 8.9, 1H, $H_{3\text{-ax}}$), 1.24 (s, 3H, CH_3) ppm; $^{13}C\{^1H\}$ NMR (151 MHz, $CDCl_3$) δ = 214.0 (*C*=O), 211.0 (*C*=O), 48.5 (C_q), 44.7 (C_9), 42.0 (C_4), 37.2 (C_6), 35.5 (C_2), 30.0 (C_7), 25.6 (C_3), 20.5 (CH_3) ppm; FT-IR (ATR) $υ_{max}$ = 2969, 2952, 2935;2911, 2875, 1729, 1702 cm^{-1}; HRMS (ESI) calcd for $[M+H]^+$ 167.1067 found 167.1073.

Procedure for 2.5 mL scale reaction using an internal standard (4a*R*,5*R*)-5,8a-dimethylhexahydronaphthalene-1,6(2*H*,5*H*)-dione (12 + 13)

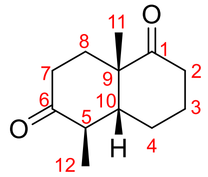

12

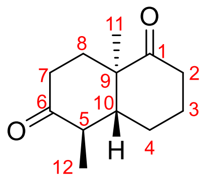

13

In a falcon tube (15 mL), the co-expressed 1907/G6PDH (0.6 mL, ≈ 8.0 mg/mL of total cell protein in Tris.HCl (50 mM, pH = 7.4) was added to a mixture of $NADP^+$ (1.4 mL, 5 mM), glucose-6-phosphate sodium salt (250 µL, 1 M) and substrate (250 µL, 200 mM in DMSO) giving final concentrations of 2.0 mg/mL of enzymes, 20 mM of substrate, 2.8 mM of $NADP^+$, 100 mM of glucose-6-phosphate sodium salt and 10% of DMSO. The solution was incubated for 20 h at 30 °C before the addition of an aqueous solution of TFA (5%, 100 µL). 1,3,5-Trimethoxybenzene (50 µL, 1 M in EtOAc) was added as internal standard. The resulting mixture was extracted with EtOAc (3 x 5 mL). The combined organic layers were washed with water, brine and dried over Na_2SO_4. After filtration and evaporation to dryness the colourless oil obtained was dissolved in $CDCl_3$ and analysed by NMR showing 90% yield of a mixture of **12**:**13** (*58:42*).

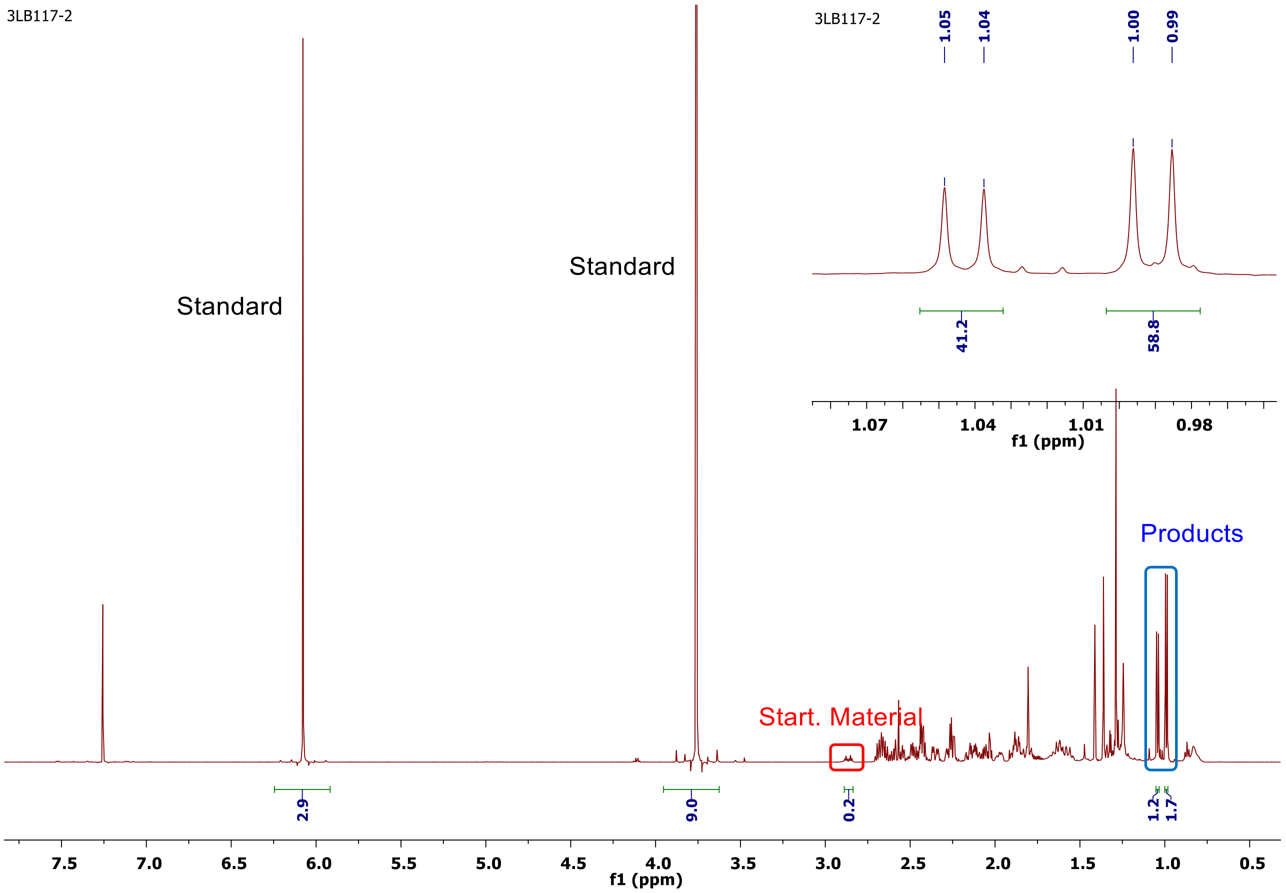

(4a*R*,5*R*,8a*R*)-5,8a-dimethylhexahydronaphthalene-1,6(2*H*,5*H*)-dione (13)

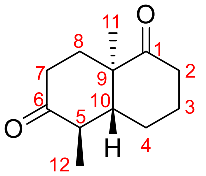

13

In a falcon tube (15 mL), the co-expressed 1907/G6PDH (0.3 mL, ≈ 8.0 mg/mL of total cell protein in Tris.HCl (50 mM, pH = 7.4) was added to a mixture of $NADP^+$ (1.4 mL, 5 mM), glucose-6-phosphate sodium salt (250 µL, 1 M) and substrate (250 µL, 100 mM in DMSO) giving final concentrations of 2.0 mg/mL of enzymes, 10 mM of substrate, 2.8 mM of $NADP^+$, 100 mM of glucose-6-phosphate sodium salt and 10% of DMSO. The solution was incubated for 5 hours before addition of more enzyme 1907/G6PDH (0.3 mL, ≈ 8.0 mg/mL of total cell protein in Tris.HCl). The mixture was then stirred for 20 h at 30 °C before the addition of an aqueous solution of TFA (5%, 100 µL). 1,3,5-Trimethoxybenzene (84 µL, 100 mM in EtOAc) was added as internal standard. The resulting mixture was extracted with EtOAc (3 x 5 mL). The combined organic layers were washed with water, brine and dried over Na_2SO_4. After filtration and evaporation to dryness the colourless oil obtained was dissolved in $CDCl_3$ and analysed by NMR. Integration revealed complete conversion of the starting material with a quantitative formation of a mixture of hydrogenated product **12**:**13** in a ration 15:85.

Determination of the conversion by NMR

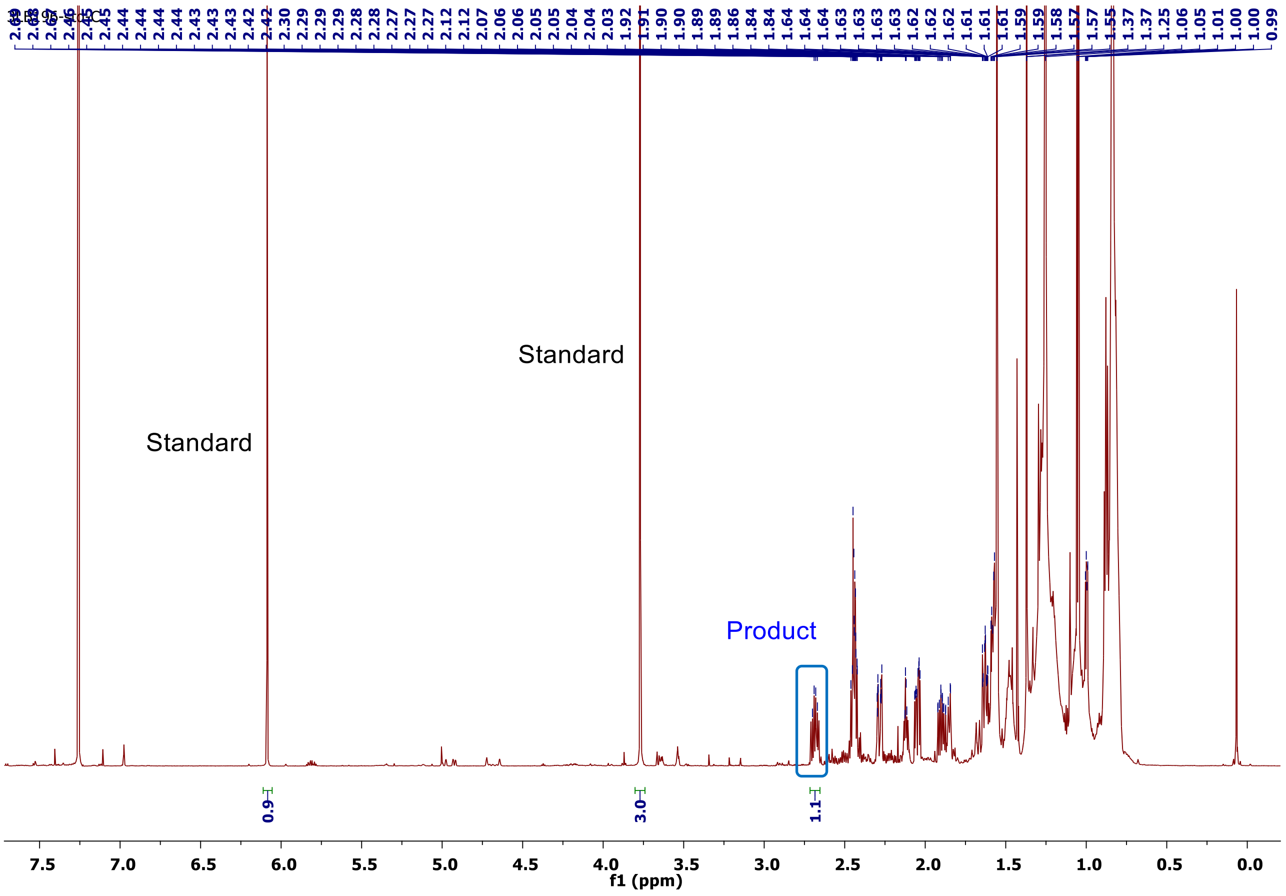

Determination of the ratio of 2 isomers by NMR

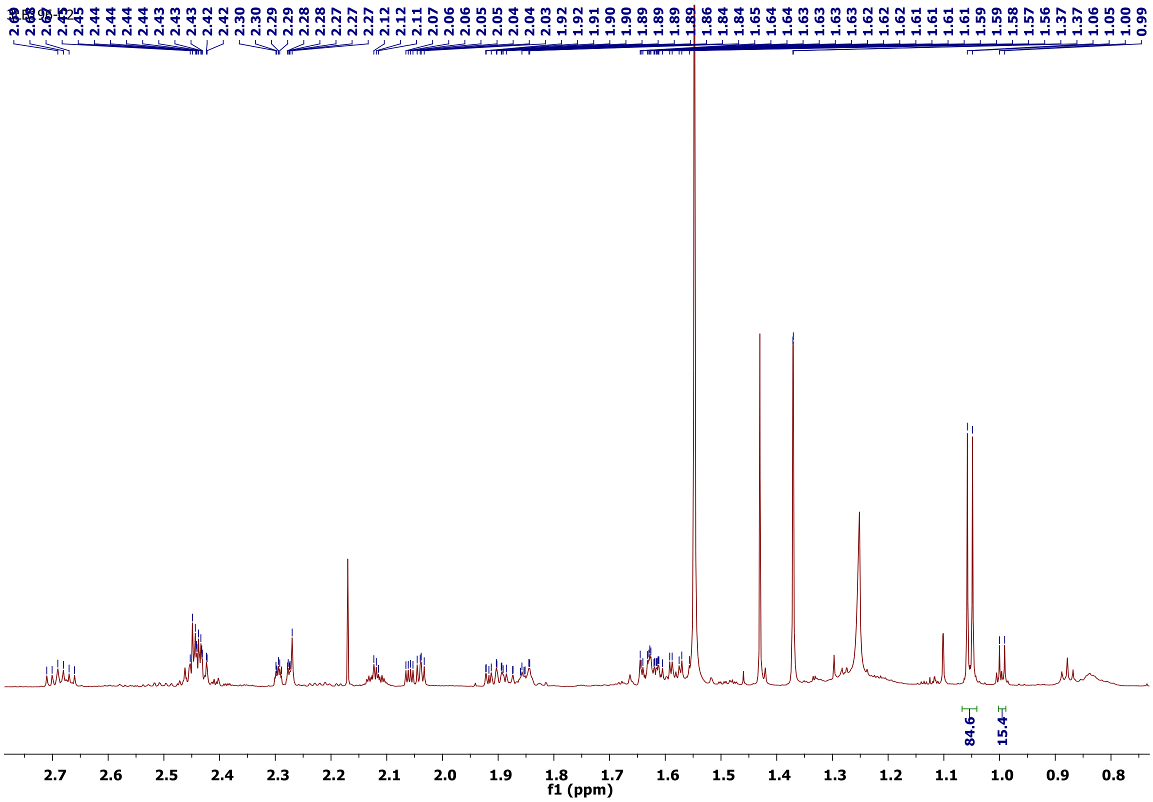

13 was purified by preparative HPLC using a Discovery® BIO Wide Pore C18 HPLC Column (25 cm x 21.2 mm, 10 μm) using a gradient of H_2O:CH_3CN (50:50 to 5:95) with both solvents supplemented with 0.1 % of TFA with a flow rate of 8 mL/min (tr_{12} = 11.9 min) ; 1H NMR (700 MHz, $CDCl_3$) δ = 2.72 – 2.64 (m, 1H, $H_{2\text{-ax}}$), 2.48 – 2.39 (m, 3H, $H_{5\text{-ax}}$, $H_{7\text{-ax\&eq}}$), 2.30 – 2.26 (m, 1H, $H_{2\text{-eq}}$), 2.15 – 2.09 (m, 1H, $H_{3\text{-eq}}$), 2.05 (ddd, *J* = 14.2, 5.3, 3.5, 1H, $H_{8\text{-eq}}$), 1.92 – 1.87 (m, 1H, $H_{8\text{-ax}}$), 1.87 – 1.82 (m, 1H, $H_{4\text{-eq}}$), 1.67 – 1.54 (m, 3H, $H_{3\text{-ax}}$, $H_{4\text{-ax}}$, $H_{10\text{-ax}}$), 1.37 (d, *J* = 0.6, 1H, CH_3C), 1.05 (d, *J* = 6.6, 1H, CH_3CH); $^{13}C\{^1H\}$ NMR (176 MHz, $CDCl_3$) δ = 214.3 (C_1), 211.3 (C_6), 51.8 (C_{10}), 48.2 (C_9), 44.7 (C_1), 37.4 (C_7), 37.2 (C_2), 33.1 (C_8), 25.9 (C_3), 24.9 (C_4), 16.4 (C_9CH_3), 11.9 ($CHCH_3$) ppm.

4a-methyl-3,4,4a,9,10,10a-hexahydrophenanthren-2(1*H*)-one (17)

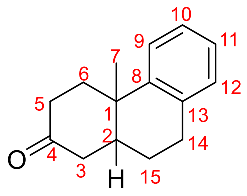

17

In a falcon tube (15 mL), the co-expressed 1907/G6PDH (480 μL, ≈ 8.0 mg/mL of total cell protein in Tris.HCl (50 mM, pH = 7.4) was added to a mixture of NADP+ (1.10 mL, 6.3 mM), glucose-6-phosphate sodium salt (170 μL, 1.47 M) and substrate (750 μL, 17 mM in DMSO) giving final concentrations of 2.0 mg/mL of enzymes, 5 mM of substrate, 2.8 mM of NADP+, 100 mM of glucose-6-phosphate sodium salt and 30% of DMSO. The solution was incubated for 20 h at 30 °C before a second addition of 1907/G6PDH (480 μL, ≈ 8.0 mg/mL of total cell protein in Tris.HCl (50 mM, pH = 7.4). The solution was then incubated for 48 hours before the addition of an aqueous solution of TFA (5%, 100 μL). 1,3,5-Trimethoxybenzene (250 μL, 17 mM in EtOAc, 0.33 eq.) was added as internal standard. The resulting mixture was extracted with Et_2O (3 x 5 mL). The combined organic layers were washed with water, brine and dried over Na_2SO_4. After filtration and evaporation to dryness the colourless oil obtained was dissolved in $CDCl_3$ and analysed by NMR. Integration of the signal corresponding to the starting material and product compared to the standard revealed that the starting material was converted at 60% and 50% of product was formed.

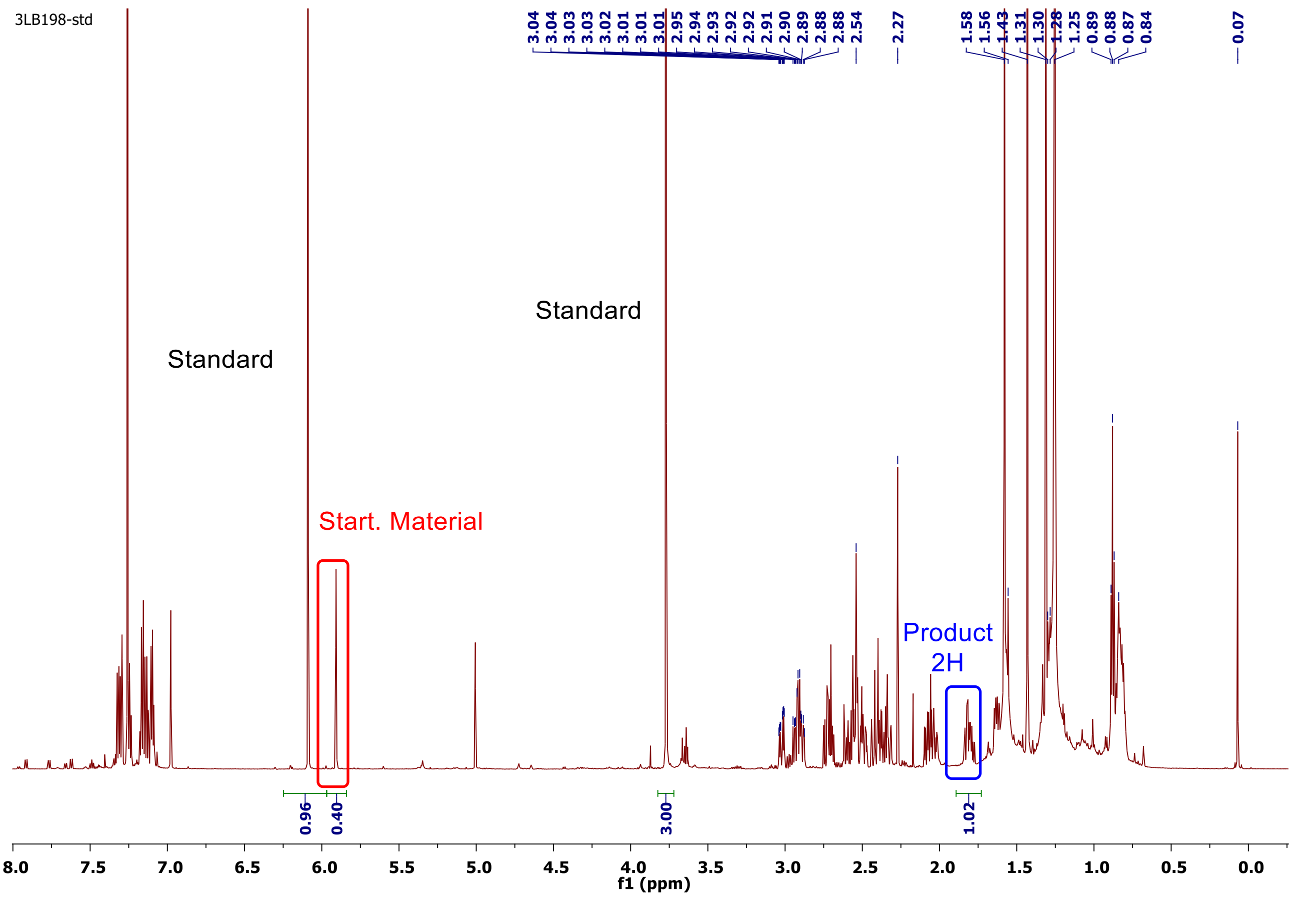

17 was purified by preparative HPLC using a Discovery® BIO Wide Pore C18 HPLC Column (25 cm x 21.2 mm, 10 µm) using a gradient of H_2O:CH_3CN (50:50 to 5:95) with both solvents supplemented with 0.1 % of TFA with a flow rate of 8 mL/min ($tr_{\mathbf{16}}$ = 15.8 min) ; ^{1}H NMR (700 MHz, $CDCl_3$) δ = 7.32 (d, *J* = 7.3, 1H, H_{12}), 7.17 (t, *J* = 7.4, 1H, H_{11}), 7.13 (td, *J* = 7.3, 1.3, 1H, H_{10}), 7.09 (d, *J* = 7.4, 1H, H_9), 2.98 – 2.87 (m, 2H, H_5), 2.63 – 2.52 (m, 2H, H6, H_3), 2.50 (dt, *J* = 5.0, 1.8, 1H, H_3), 2.45 – 2.38 (m, 1H, H_{15}), 2.32 (ddd, *J* = 15.3, 3.9, 2.2, 1H, H_{15}), 2.04 (ddd, *J* = 14.0, 6.7, 3.3, 1H, H_2), 1.81 (tdd, *J* = 16.5, 11.6, 5.4, 2H, H_{14}, H_6), 1.65 – 1.61 (m, 1H, H_{14}), 1.31 (s, 3H, $CH_{3\text{-}7}$) ; ^{13}C{^{1}H} NMR (176 MHz, $CDCl_3$) δ = 211.2 (*C*=O), 145.6 (C_8), 135.3 (C_{13}), 129.6 (C_9), 126.2 (C_{10}), 126.1 (C_{11}), 125.4 (C_{12}), 44.8 (C_{15}), 42.5 (C_2), 38.5 (C_3), 37.5 (C_6), 36.5 ($C_{q\text{-}1}$), 29.7 (*C*5), 25.8 (C_{14}), 21.3 (CH_3) ppm; HRMS (ESI) calcd for $C_{15}H_{19}O$ $[M+H]^+$ 215.1436 found 215.1434.

Determination of the configuration of 9, 12 and 15 by NMR

Accurate values of ^{1}H NMR vicinal *J* couplings for **9, 12** and **15** were determined using iterative full lineshape analysis (**Figures S9 - S11**).[†,18,19]

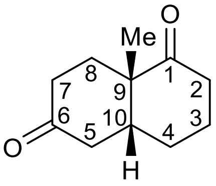

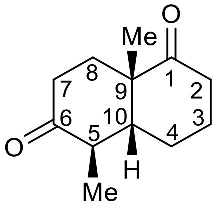

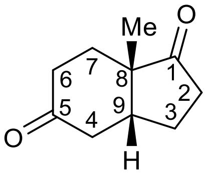

9 **12** **15**

Once all the *J* couplings were determined (included in **Figures S9 - S11**), we then considered three possible molecular geometries together with the predicted $^3J_{HH}$ values using Karplus-type equations of Haasnoot *et al.*[20]

Compound **9**

For compound **9**, the values of *trans*-$^3J_{HH}$ couplings for proton 10 calculated for optimised molecular geometries of conformers using the MMX force field[‡] are shown below (*cis* and *trans* in the configuration labelling below refer to *cis*- and *trans*-fusion of the two cycles, while *cis1* and *cis2* indicate to the two possible conformers for the *cis*-fusion of the two cycles):

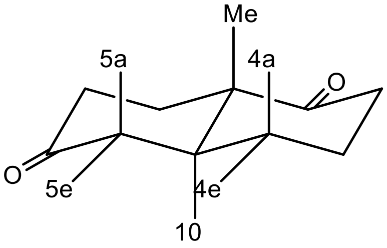

9-*trans*

^3J(5a,10)=12.33 Hz
^3J(4a,10)=12.36 Hz

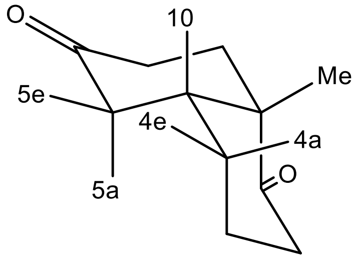

9-*cis1*

^3J(5a,10)=12.32 Hz
^3J(4e,10)=2.51 Hz

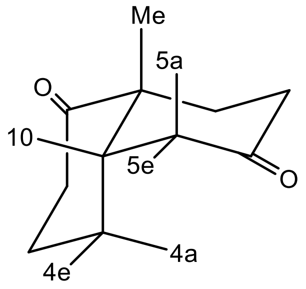

9-*cis2*

^3J(5e,10)=1.99 Hz
^3J(4a,10)=12.34 Hz

The experimental values of *trans*-$^3J_{HH}$ couplings for proton 10 were 10.56 Hz with proton 5a and 4.75 Hz for proton 4e. These values agree best with those of **9-*cis1*** conformer shown above. Some decrease of the experimental *trans*-^3J(5,10) coupling and increase of the experimental *trans*-^3J(4,10) coupling compared to predicted values suggest that there is a conformational equilibrium between **9-**

† gNMR, Version 5.0.6; NMR Simulation Program, Budzelaar, P. H. M., **2006**

‡ (a) M.F. Schlecht, *Molecular Modeling on the PC*, Wiley-VCH, New York, **1998**. (b) Software used: PCMODEL (version 8.5, Serena Software), Bloomington, IN, **2003**.

cis1 and **9-*cis2*** conformers in $CDCl_3$ solution with the preference of the **9-*cis1*** conformer. Using the predicted boundary values of *trans*-$^3J_{HH}$ couplings shown above, the populations of **9-*cis1*** and **9-*cis2*** conformers in $CDCl_3$ solution can be estimated to be 80% and 20%, respectively (83.0% and 17.0% from *trans*-^3J(5,10) coupling and 77.2% and 22.8% from *trans*-^3J(4,10) coupling).

Figure S9. The overlaid view of experimental (red) and fitted (black) ^{1}H NMR lineshapes of **9** (in $CDCl_3$, 700 MHz, 25 °C). The screenshot of the table of fitting results is also shown (chemical shifts are in ppm; *J* couplings and linewidths are in Hz).

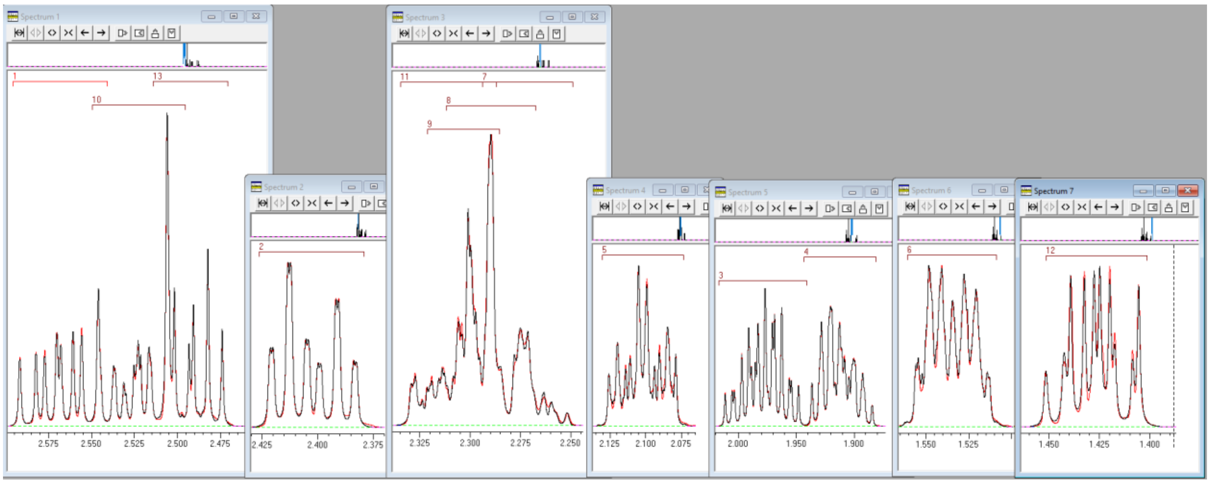

#	Nucleus	n	Shift	Width	J[1]	J[2]	J[3]	J[4]	J[5]	J[6]	J[7]	J[8]	J[9]	J[10]	J[11]	J[12]
1	1H	1	2.568	0.82												
			s2a	w2a												
2	1H	1	2.403	0.78	-15.07											
			s2e	w2e	j2a2e											
3	1H	1	1.979	0.82	10.31	5.62										
			s3a	w3a	j2a3a	j2e3a										
4	1H	1	1.912	0.87	6.50	5.59	-14.06									
			s3e	w3e	j2a3e	j2e3e	j3a3e									
5	1H	1	2.102	0.77	-0.53	0.00	10.57	4.35								
			s4a	w4a	j2a4a	-	j3a4a	j3e4a								
6	1H	1	1.535	0.90	0.00	-1.06	4.23	5.76	-14.29							
			s4e	w4e	-	j2e4e	j3a4e	j3e4e	j4a4e							
7	1H	1	2.271	0.68	0.00	-0.58	0.53	0.62	4.05	5.49						
			s10	w10	-	j2e10	j3a10	j3e10	j4a10	j4e10						
8	1H	1	2.290	0.61	0.00	0.00	0.00	0.00	0.00	0.00	10.56					
			s5a	w5a	-	-	-	-	-	-	j5a10					
9	1H	1	2.303	0.85	0.00	0.00	0.00	0.00	0.00	0.00	4.75	-14.39				
			s5e	w5e	-	-	-	-	-	-	j5e10	j5a5e				
10	1H	1	2.522	0.68	0.00	0.00	0.00	0.00	0.00	0.00	0.00	1.36	0.00			
			s7a	w7a	-	-	-	-	-	-	-	j5a7a	-			
11	1H	1	2.311	0.85	0.00	0.00	0.00	0.00	0.00	0.00	0.00	0.00	1.88	-14.61		
			s7e	w7e	-	-	-	-	-	-	-	-	j5e7e	j7a7e		
12	1H	1	1.426	1.02	0.00	0.00	0.00	0.00	0.00	0.00	0.00	0.00	0.00	10.35	5.40	
			s8a	w8a	-	-	-	-	-	-	-	-	-	j7a8a	j7e8a	
13	1H	1	2.492	0.79	0.00	0.00	0.00	0.00	0.00	0.00	0.00	0.00	0.00	5.97	6.02	-13.76
			s8e	w8e	-	-	-	-	-	-	-	-	-	j7a8e	j7e8e	j8a8e

Figure S10. The overlaid view of experimental (red) and fitted (black) ^{1}H NMR lineshapes of **12** (in $CDCl_3$, 700 MHz, 25 °C). To simplify the multiplicity of the signal due to the axial proton 5a at 2.25 ppm, the lineshape of the corresponding multiplet from the ^{1}H NMR spectrum with homonuclear decoupling from 5-Me protons was used in iterative fittings. The screenshot of the table of fitting results is also shown (chemical shifts are in ppm; *J* couplings and linewidths are in Hz).

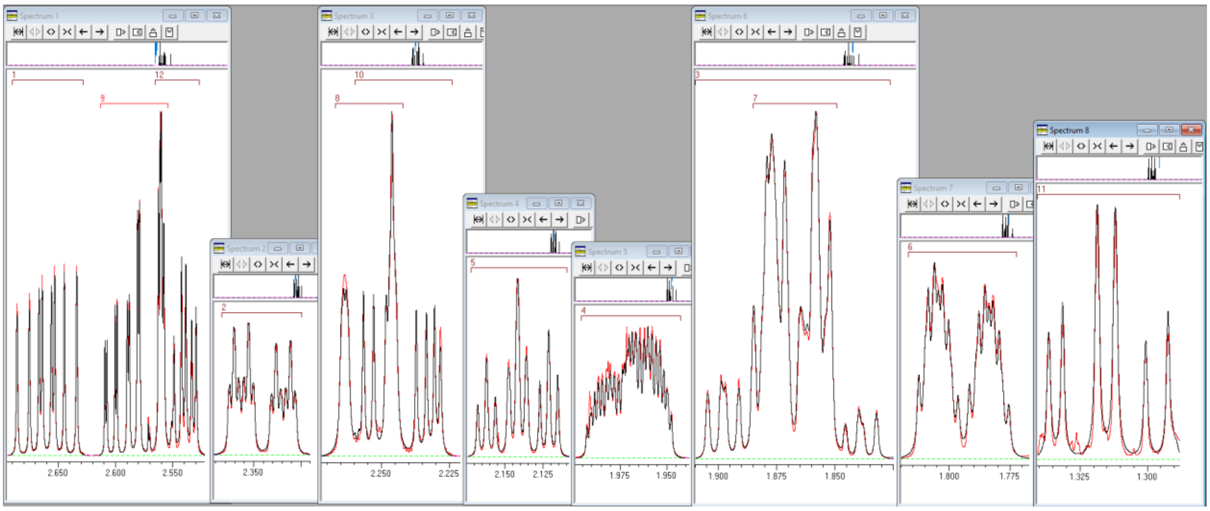

#	Nucleus	n	Shift	Width	J[1]	J[2]	J[3]	J[4]	J[5]	J[6]	J[7]	J[8]	J[9]	J[10]	J[11]
1	1H	1	2.659	0.64											
			s2a	w2a											
2	1H	1	2.346	0.80	-15.44										
			s2e	w2e	j2a2e										
3	1H	1	1.870	0.65	13.55	5.36									
			s3a	w3a	j2a3a	j2e3a									
4	1H	1	1.969	0.74	7.55	1.69	-14.06								
			s3e	w3e	j2a3e	j2e3e	j3a3e								
5	1H	1	2.141	0.89	0.35	0.00	13.98	4.28							
			s4a	w4a	j2a4a	-	j3a4a	j3e4a							
6	1H	1	1.795	1.09	0.00	1.83	4.05	2.74	-14.77						
			s4e	w4e	-	j2e4e	j3a4e	j3e4e	j4a4e						
7	1H	1	1.867	0.85	0.00	0.53	0.29	1.20	3.83	2.80					
			s10	w10	-	j2e10	j3a10	j3e10	j4a10	j4e10					
8	1H	1	2.254	0.83	0.00	0.00	0.60	0.00	0.00	0.00	12.36				
			s5a	w5a	-	-	j3a5a	-	-	-	j5a10				
9	1H	1	2.583	0.50	0.00	0.00	0.00	0.00	0.00	0.00	0.00	1.24			
			s7a	w7a	-	-	-	-	-	-	-	j5a7a			
10	1H	1	2.242	0.56	0.00	0.00	0.00	0.00	0.00	0.00	0.00	0.00	-13.68		
			s7e	w7e	-	-	-	-	-	-	-	-	j7a7e		
11	1H	1	1.315	1.01	0.00	0.00	0.00	0.00	0.00	0.00	0.00	0.00	14.16	4.70	
			s8a	w8a	-	-	-	-	-	-	-	-	j7a8a	j7e8a	
12	1H	1	2.546	0.88	0.00	0.00	0.00	0.00	0.00	0.00	0.00	0.00	6.21	2.74	-13.69
			s8e	w8e	-	-	-	-	-	-	-	-	j7a8e	j7e8e	j8a8e

Figure S11. The overlaid view of experimental (red) and fitted (black) ^{1}H NMR lineshapes of **16** (in $CDCl_3$, 700 MHz, 25 °C). The screenshot of the table of fitting results is also shown (chemical shifts are in ppm; *J* couplings and linewidths are in Hz). Due to the overlap of multiplets of protons 7e (number 11 in in the spin system used) and 3a (number 3) with the peak due to water, the corresponding signal at 1.626 ppm with the linewidth of 3.12 Hz was also included in spectral fittings (denoted as 2-1 in Spectrum 5 below).

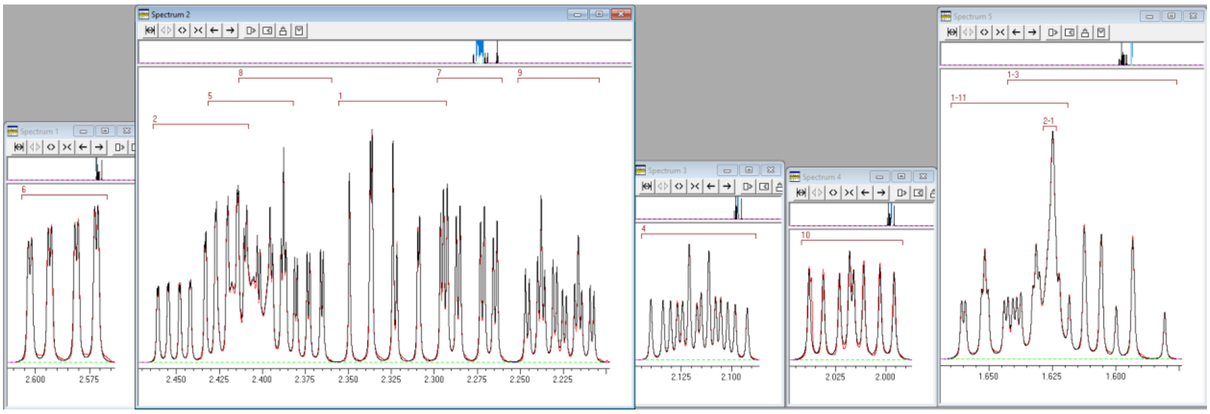

#	Nucleus	n	Shift	Width	J[1]	J[2]	J[3]	J[4]	J[5]	J[6]	J[7]	J[8]	J[9]	J[10]
1	1H	1	2.324	0.53										
			s2a	w1										
2	1H	1	2.436	0.52	-19.36									
			s2e	w2	j2a2e									
3	1H	1	1.609	0.60	8.48	8.92								
			s3a	w3	j2a3a	j2e3a								
4	1H	1	2.116	0.59	9.36	4.14	-13.38							
			s3e	w4	j2a3e	j2e3e	j3a3e							
5	1H	1	2.407	0.84	0.22	0.60	8.92	6.87						
			s9	w5	j2a9	j2e9	j3a9	j3e9						
6	1H	1	2.587	0.52	0.00	0.20	0.25	0.35	6.46					
			s4a	w6	-	j2e4a	j3a4a	j3e4a	j4a9					
7	1H	1	2.279	0.54	0.00	0.09	0.06	0.27	5.22	-15.11				
			s4e	w7	-	j2e4e	j3a4e	j3e4e	j4e9	j4a4e				
8	1H	1	2.387	0.58	0.00	0.00	0.00	0.00	0.00	1.11	0.00			
			s6a	w8	-	-	-	-	-	j4a6a	-			
9	1H	1	2.228	0.66	0.00	0.00	0.00	0.00	0.00	0.00	1.58	-15.11		
			s6e	w9	-	-	-	-	-	-	j4e6e	j4a4e		
10	1H	1	2.017	1.00	0.00	0.00	0.00	0.00	0.00	0.00	0.00	10.63	4.94	
			s7a	w10	-	-	-	-	-	-	-	j6a7a	j6e7a	
11	1H	1	1.642	0.68	0.00	0.00	0.00	0.00	1.06	0.00	-0.22	5.48	6.31	-14.15
			s7e	w11	-	-	-	-	j7e9	-	j4e7e	j6a7e	j6e7e	j7a7e

Figure S12. (a) 1D NOESY spectrum of **9** with a selective excitation of the methyl protons at 1.35 ppm (mixing time 300 ms); (b) ^{1}H NMR spectrum of **9**. Spectra were recorded in $CDCl_3$ at 25 °C using a 700 MHz spectrometer.

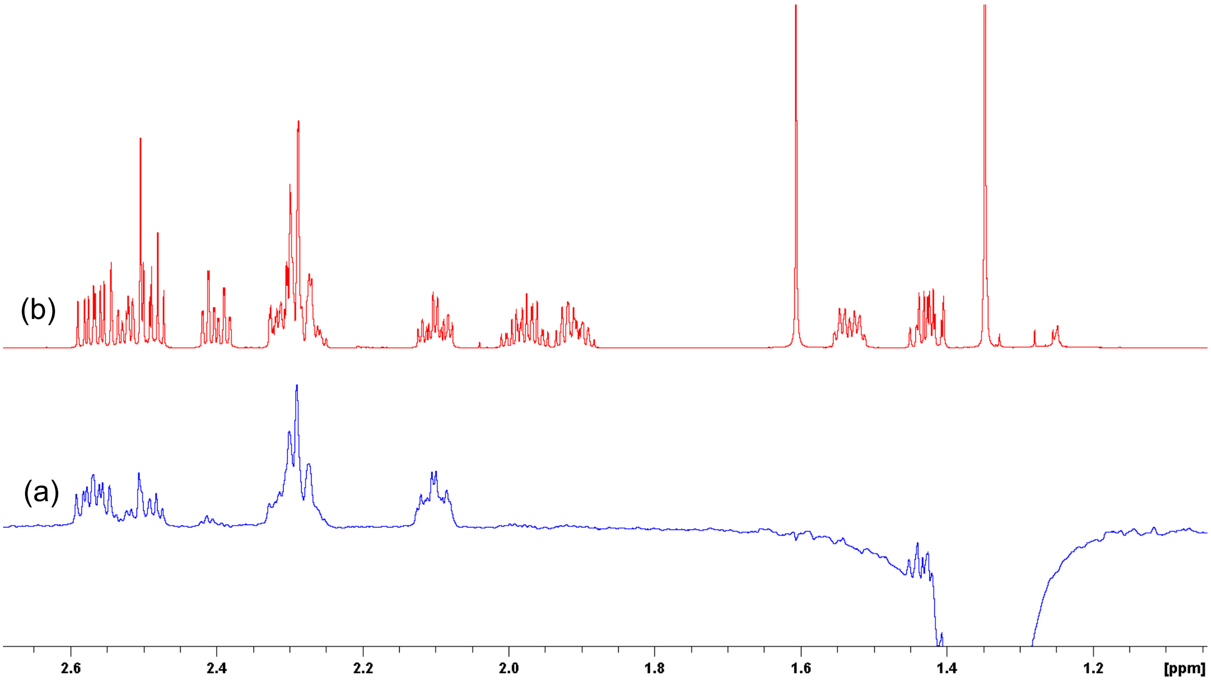

Assuming the predicted boundary values are accurate within ±0.5 Hz,[21] the uncertainty in the population determinations of two conformers is estimated to be ±4%. The preference of the **9-*cis1*** conformer is also confirmed by a 1D NOESY spectrum (**Figure S12**), which shows NOEs at 2.10 ppm for the proton pair (Me, 4a) and at 2.57 ppm for the proton pair (Me, 2a).

Compound **12**

In a similar manner we have also considered predicted values of ^{3}J couplings in compound **12** for various plausible configurations and conformations (the first occurrence of *cis* and *trans* in the configuration labelling below refers to *cis*- and *trans*-fusion of the two cycles, while the second occurrence of *cis* and *trans* reflects the configuration of the two methyl groups; *cis1* and *cis2* indicate to the two possible conformers for the *cis*-fusion of the two cycles):

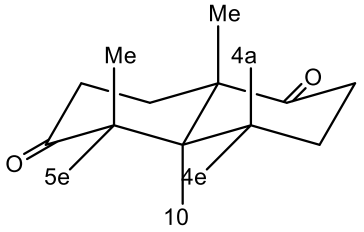

12-*trans-cis*

^{3}J(5e,10)=5.19 Hz
^{3}J(4a,10)=12.29 Hz

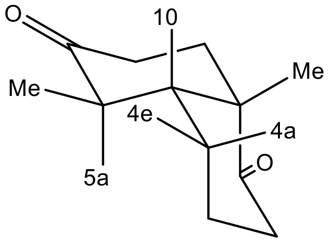

12-*cis1-cis*

^{3}J(5a,10)=12.34 Hz
^{3}J(4e,10)=2.96 Hz

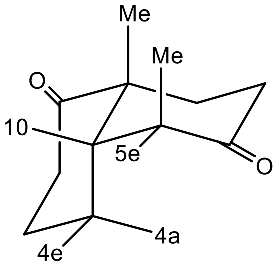

12-*cis2-cis*

^{3}J(5e,10)=3.59 Hz
^{3}J(4a,10)=12.32 Hz

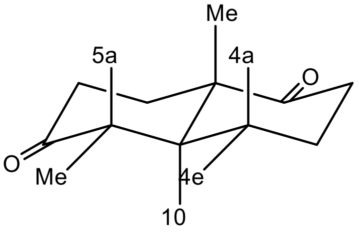

12-*trans-trans*

^{3}J(5a,10)=12.33 Hz
^{3}J(4a,10)=12.36 Hz

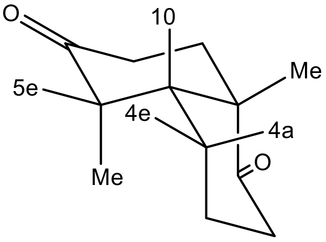

12-*cis1-trans*

^{3}J(5e,10)=5.28 Hz
^{3}J(4e,10)=1.59 Hz

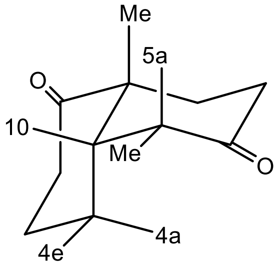

12-*cis2-trans*

^{3}J(5a,10)=3.59 Hz
^{3}J(4a,10)=12.32 Hz

The experimental values of *trans*-$^{3}J_{HH}$ couplings for proton 10 were 12.36 Hz with proton 5a and 2.80 Hz for proton 4e. These values agree best with those of **12-*cis1-cis*** conformer shown above and, unlike compound **9**, the presence of the second methyl group in position 5 leads to the predominance of the **12-*cis1-cis*** conformation with a nearly 100% population, as the second possible conformer **12-*cis2-cis*** is destabilised by interactions between two axial methyl groups. The preference of the **12-*cis1-cis*** conformer is also confirmed by a 2D NOESY spectrum (**Figure S13**), which shows NOEs at (1.28 ppm, 2.14 ppm) for the proton pair (9-Me, 4a) and at (1.28 ppm, 2.66 ppm) for the proton pair (9-Me, 2a).

Figure S13. 2D NOESY spectrum of **12** in $CDCl_3$ (mixing time 600 ms, 700 MHz) at 25 °C.

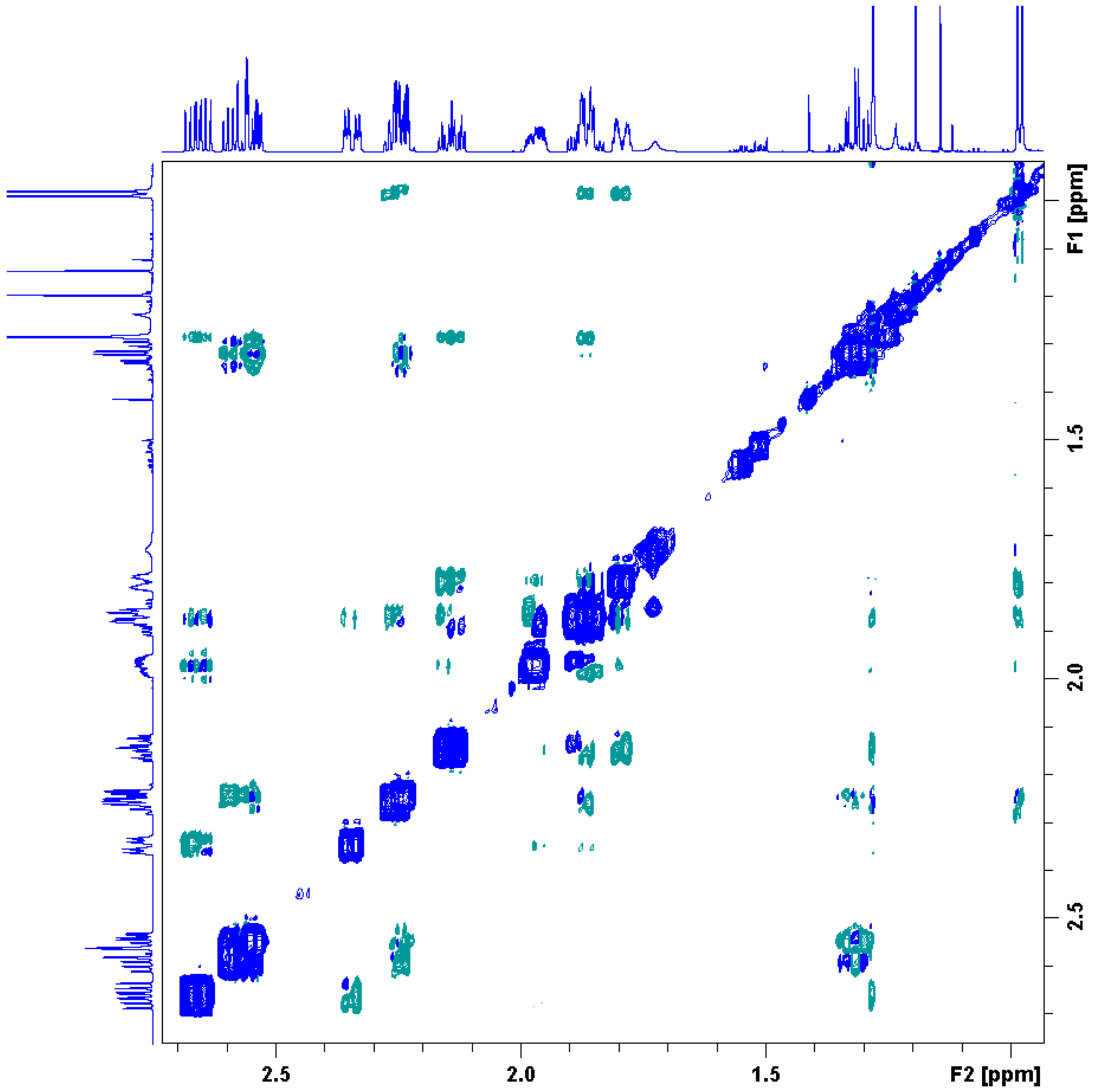

The trans-isomer **13** was also considered, which is expected to be conformationally homogeneous.

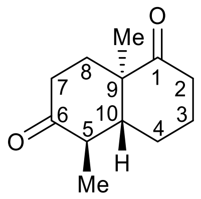

13

From the analysis of the NMR data (**Figure S14**), the experimental values of $^3J_{HH}$ couplings for proton 10 were 12.34 Hz with proton 4a, 12.21 Hz with proton 5a and 3.41 Hz with proton 4e. A ^4J coupling of 0.73 Hz was observed between protons 9-Me and 8a, which is in favour of the axial orientation of the 9-Me group. A 2D NOESY spectrum of **13** (Figure S15) showed NOEs for proton pairs (9-Me, 2a),

(9-Me, 4a), (9-Me, 5a), (9-Me, 7a) and (9-Me, 8e) at (1.36 ppm, 2.68 ppm), (1.36 ppm, 1.62 ppm), (1.36 ppm, 2.44 ppm), (1.36 ppm, 2.43 ppm) and (1.36 ppm, 2.04 ppm), respectively. These $^{3}J_{HH}$ couplings and NOEs are in favour of the *trans*-fusion of the two six-membered rings together with the *trans*-configuration of the two methyl groups, as shown below:

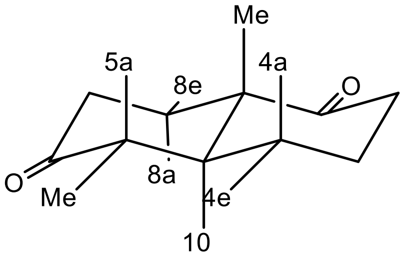

13

Figure S14. The overlaid view of experimental (red) and fitted (black) ^{1}H NMR lineshapes of 13 (in $CDCl_3$, 700 MHz, 25 °C). To simplify the multiplicity of the signal due to the axial proton 5a at 2.25 ppm, the lineshape of the corresponding multiplet from the ^{1}H spectrum with homonuclear decoupling from 5-Me protons was used in iterative fittings. Similarly, to simplify the multiplicity of the signal due to the axial proton 8a at 1.89 ppm (with ^4J(8a,9-Me)=0.73 Hz), the lineshape of the corresponding multiplet from the 1H spectrum with homonuclear decoupling from 9-Me protons was used in iterative fittings. The screenshot of the table of fitting results is also shown (chemical shifts are in ppm; J couplings and linewidths are in Hz). The singlet at 1.585 ppm due to water in $CDCl_3$ was included as "molecule 2" in iterative fittings (see 2-1 in Spectrum 7 below).

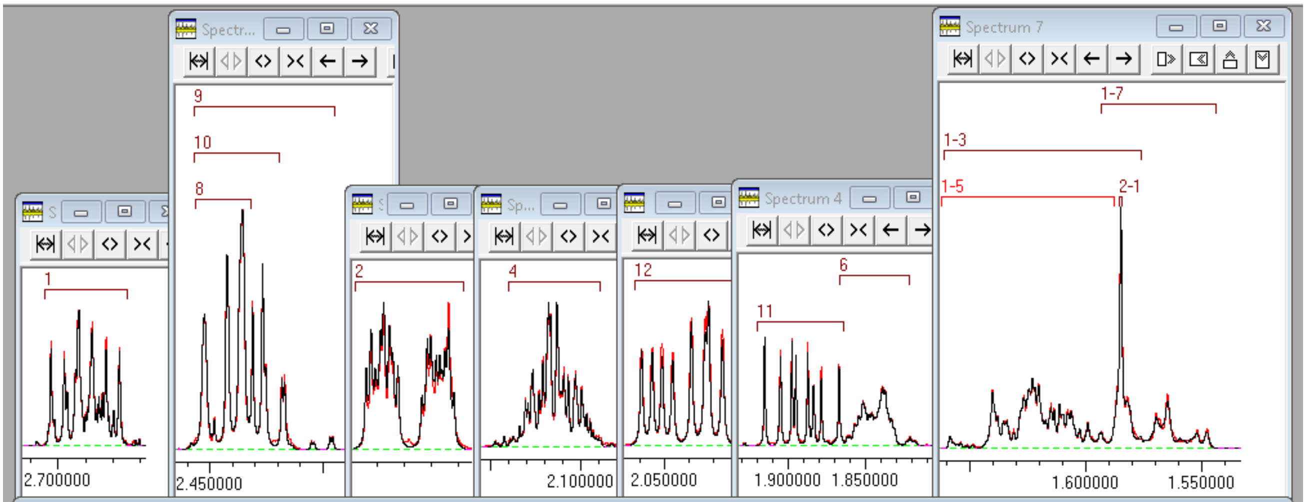

Molecule 1

#	Nucleus	n	Shift	Width	J[1]	J[2]	J[3]	J[4]	J[5]	J[6]	J[7]	J[8]	J[9]	J[10]	J[11]
1	1H	1	2.679829	0.72											
			s2a	w2a											
2	1H	1	2.277914	0.70	-14.45										
			s2e	w2e	j2a2e										
3	1H	1	1.618545	0.78	14.04	4.95									
			s3a	w3	j2a3a	j2e3a									
4	1H	1	2.114775	0.74	6.84	2.33	-13.74								
			s3e	w3e	j2a3e	j2e3e	j3a3e								
5	1H	1	1.624850	0.69	-0.29	-0.43	13.11	4.13							
			s4a	w4a	j2a4a	j2e4a	j3a4a	j3e4a							
6	1H	1	1.844101	1.06	-0.32	1.40	4.45	2.90	-13.83						
			s4e	w4e	j2a4e	j2e4e	j3a4e	j3e4e	j4a4e						
7	1H	1	1.568540	1.25	0.00	0.00	-0.40	-0.16	12.34	3.41					
			s10	w10	-	-	j3a10	j3e10	j4a10	j4e10					
8	1H	1	2.444132	0.92	0.00	0.00	0.00	0.00	-0.34	-0.15	12.21				
			s5a	w5a	-	-	-	-	j4a5a	j4e5a	j5a10a				
9	1H	1	2.425872	0.70	0.00	0.00	0.00	0.00	0.00	0.00	0.00	1.00			
			s7a	w7a	-	-	-	-	-	-	-	j5a7a			
10	1H	1	2.438446	0.60	0.00	0.00	0.00	0.00	0.00	0.00	0.00	-0.12	-15.00		
			s7e	w7e	-	-	-	-	-	-	-	j5a7e	j7a7e		
11	1H	1	1.892113	0.62	0.00	0.00	0.00	0.00	0.00	0.00	-0.01	-0.08	14.52	5.07	
			s8a	w8a	-	-	-	-	-	-	j8a10	j5a8a	j7a8a	j7e8a	
12	1H	1	2.042097	0.76	0.00	0.00	0.00	0.00	0.00	0.00	0.16	-0.18	6.55	2.30	-14.19
			s8e	w8e	-	-	-	-	-	-	j8e10	j5a8e	j7a8e	j7e8e	j8a8e

Figure S15. 2D NOESY spectrum of **13** in $CDCl_3$ (mixing time 600 ms, 700 MHz) at 25 °C.

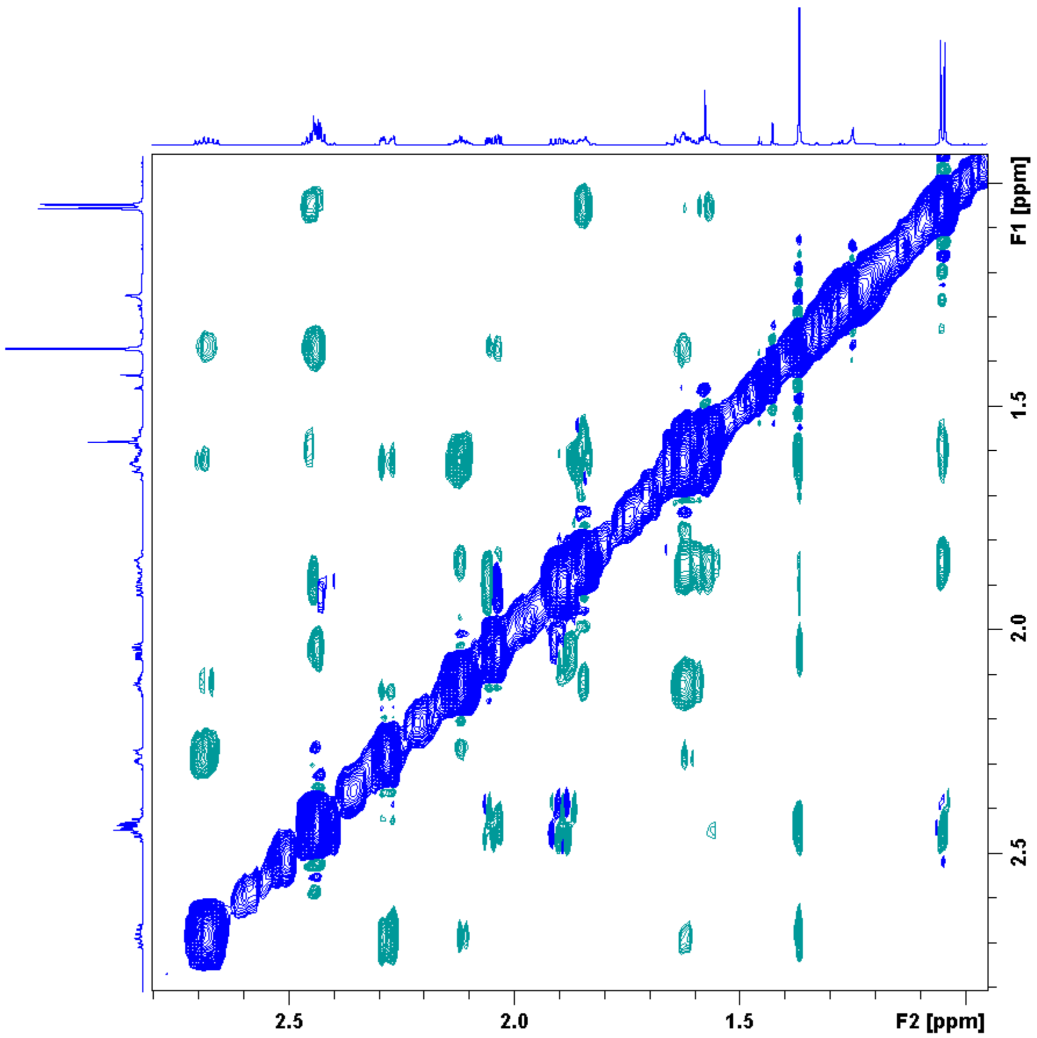

Compound **15**

For compound **15**, the values of *trans*-$^3J_{HH}$ couplings for proton 9 calculated for optimised molecular geometries of conformers using MMX force field,§ are shown below:

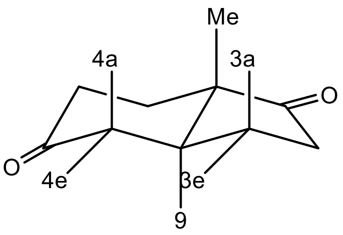

14-*trans*

^3J(4a,9)=12.35 Hz
^3J(3a,9)=11.96 Hz

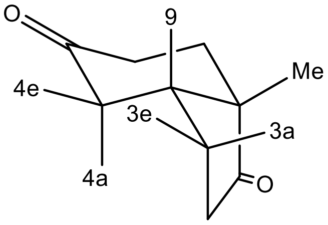

14-*cis1*

^3J(4a,9)=11.43 Hz
^3J(3e,9)=1.03 Hz

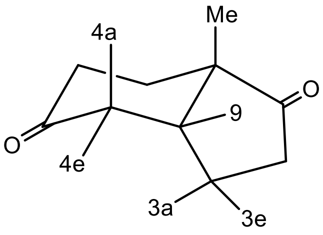

14-*cis2*

^3J(4e,9)=1.66 Hz
^3J(3a,9)=11.85 Hz

The experimental values of *trans*-$^3J_{HH}$ couplings for the proton pairs (4a,9) was less than 6.5 Hz, hence the *trans*-fusion of the two cycles with the predominance of the conformer shown above as ***15-trans*** can be ruled out. This suggests that the methyl group and proton 9 have a ***cis***-configuration.

Figure S16. (a) 1D NOESY spectrum of **15** with selective excitation of the methyl protons at 1.24 ppm (mixing time 300 ms); (b) 1H NMR spectrum of **15**. Spectra were recorded in $CDCl_3$ at 25 °C using a 700 MHz spectrometer. From these spectra, protons of the 3-CH_2 and 4-CH_2 groups having a cis-configuration with respect to the methyl group (as well as with proton 9) can be identified as those resonating at 2.12 ppm and 2.59 ppm, respectively.

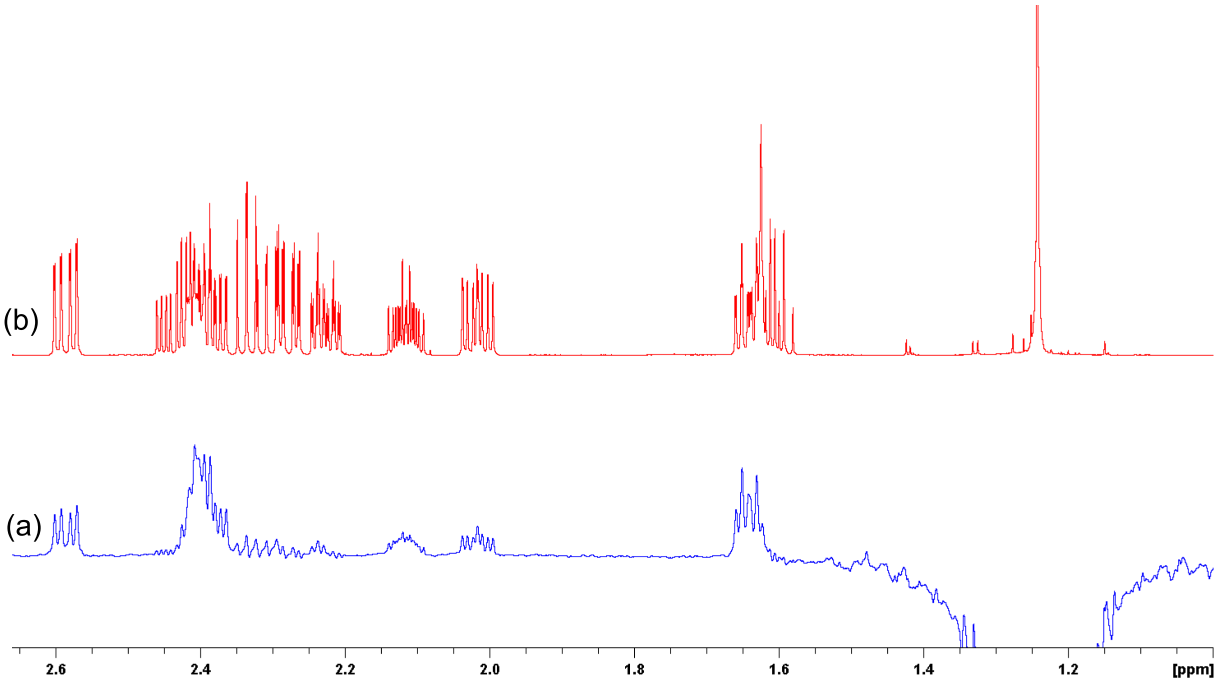

From the 1D NOESY spectrum (**Figure S16**), protons of the 3-CH_2 and 4-CH_2 groups having a *cis*- or *trans*-configuration with respect to the methyl group (hence with proton 9) can be identified, thus allowing to assign *cis*- and *trans*-$^3J_{HH}$ couplings of proton 9. The experimental values of *trans*-$^3J_{HH}$ couplings for proton pairs (4,9) and (3,9) were 5.22 Hz and 8.92 Hz, respectively. These values are intermediate between those predicted for **15-*cis1*** and **15-*cis2*** conformers shown above, suggesting a conformational equilibrium between these two conformers. Using the predicted boundary values of *trans*-$^3J_{HH}$ couplings shown above, the populations of **15-*cis1*** and **15-*cis2*** conformers in $CDCl_3$ solution can be estimated as 32±5% and 68±5%, respectively (36.4% and 63.6% from the *trans*-^3J(4,9) coupling and 27.1% and 72.9% from the *trans*-^3J(3,9) coupling).

ANALYTICAL METHODS

Chiral GC analysis (Cyclohexyl-derivatives)

Analyses were performed using an Agilent 7820A Gas Chromatograph equipped with a chiral column (Beta DEX 225, fused silica capillary column 30 m x 0.25 mm x 0.25 µm). The samples (5 µL) were injected with an autosampler tower G4513A and applied by split injection (ratio 20:1) at an injection temperature of 250 °C with a split flow of 36 mL/min and an oven temperature of 80 °C. For every substrate the sequence applied was as followed:

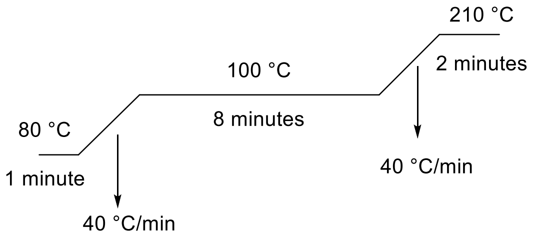

Table S4. Cyclohexyl-derivatives – retention times.

Substrate	Retention time (min)	Product	Retention time (min)
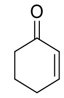	11.81	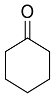	10.26
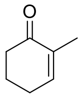	9.93	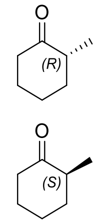	Enant. 1 = 6.70<br>Enant. 2 = 6.88
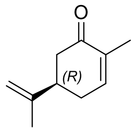	12.23	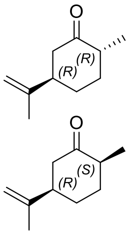	Diast. 1 = 11.78<br>Diast. 2 = 11.94
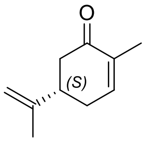	12.20	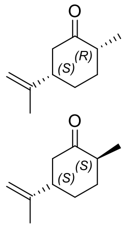	Diast. 1 = 11.73<br>Diast. 2 = 11.94

Figure S17. Calibration curve for cyclohexanone (**1**).

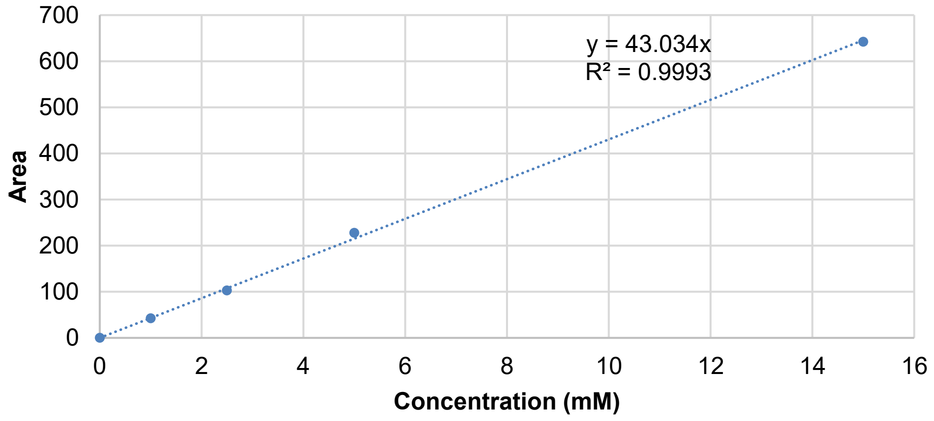

Figure S18. Calibration curve for 2-methyl-cyclohexanone (**2**).

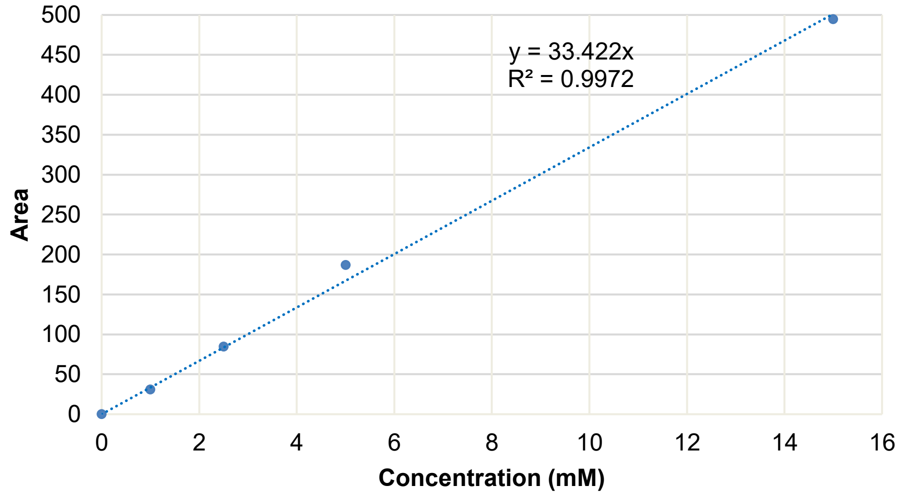

Figure S19. Calibration curve for dihydrocarvone (**9**).

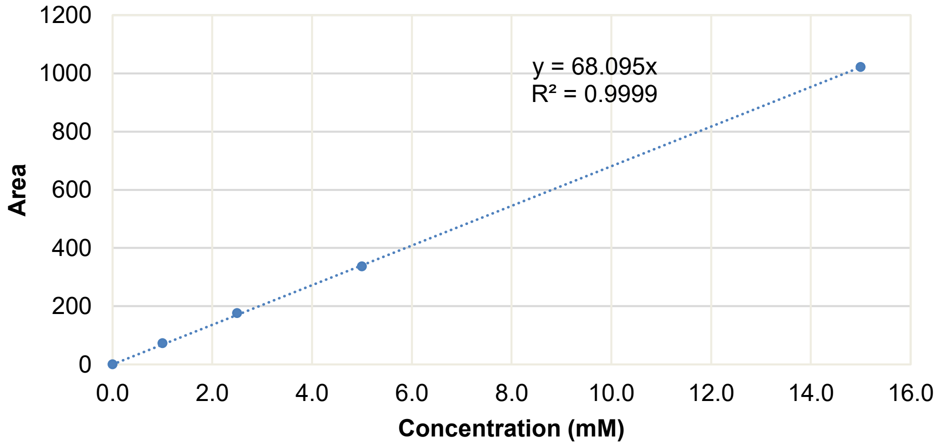

Chromatograms

The selectivity of the product was determined using the results from the biotransformation using NCR and by comparing with literature results reported for NCR (R selectivity).[22–25]

Figure S20. *Rac*-2-methylcyclohexanone (commercial).

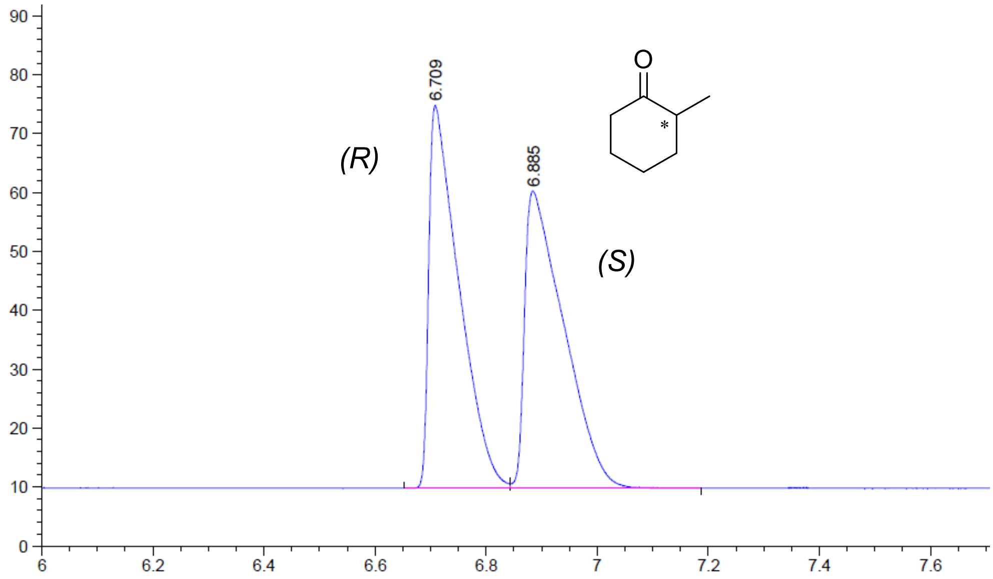

Peak #	RetTime [min]	Type	Width [min]	Area [pA*s]	Height [pA]	Area %
1	6.709	BV	0.0569	246.25069	65.08887	49.78034
2	6.885	VB	0.0701	248.42389	50.54990	50.21966
Totals :				494.67458	115.63876	

Figure S21. Transformation of 2-methylcyclohex-2-en-1-one (**2**) with NCR.

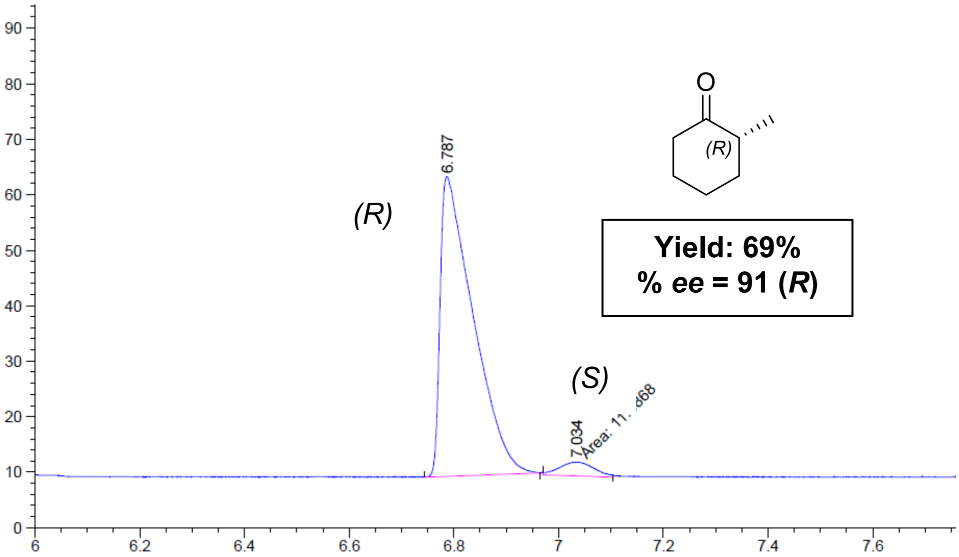

Peak #	RetTime [min]	Type	Width [min]	Area [pA*s]	Height [pA]	Area %
1	6.787	BB	0.0591	236.18710	53.89630	95.40066
2	7.034	MM	0.0771	11.38677	2.46290	4.59934
Totals :				247.57387	56.35920	

Figure S22. Transformation of 2-methylcyclohex-2-en-1-one (**2**) with pQR1907.

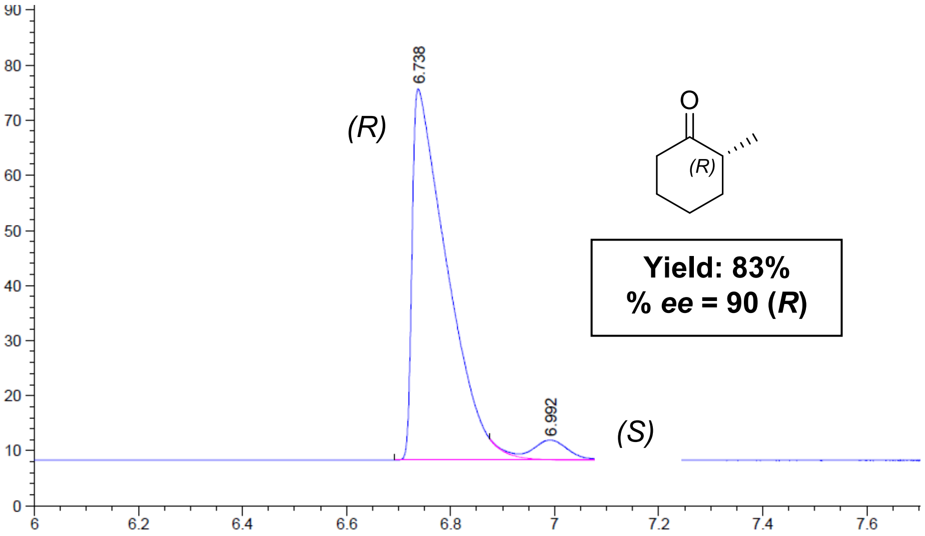

Peak #	RetTime [min]	Type	Width [min]	Area [pA*s]	Height [pA]	Area %
1	6.738	BV R	0.0655	314.43442	67.51037	94.78343
2	6.992	VB E	0.0739	17.30543	3.58331	5.21657
Totals :				331.73985	71.09368	

Figure S23. Transformation of carvone derivative *R*-**5** with NCR.

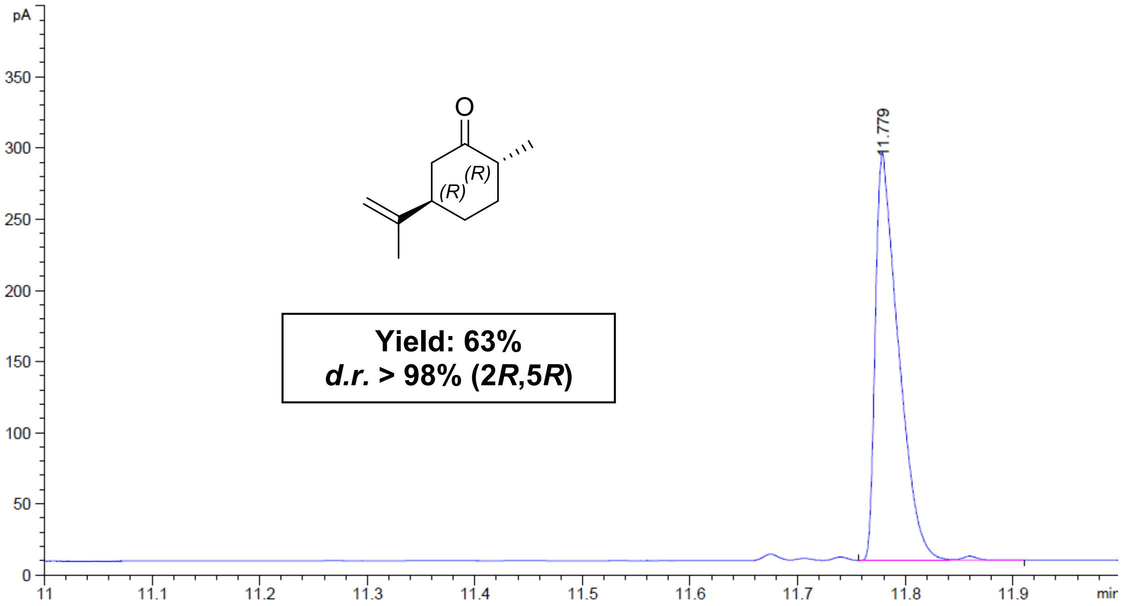

Peak #	RetTime [min]	Type	Width [min]	Area [pA*s]	Height [pA]	Area %
1	11.779	BV R	0.0234	428.65027	280.54117	1.000e2
Totals :				428.65027	280.54117	

Figure S24. Transformation of carvone derivative *R*-**5** with pQR1445.

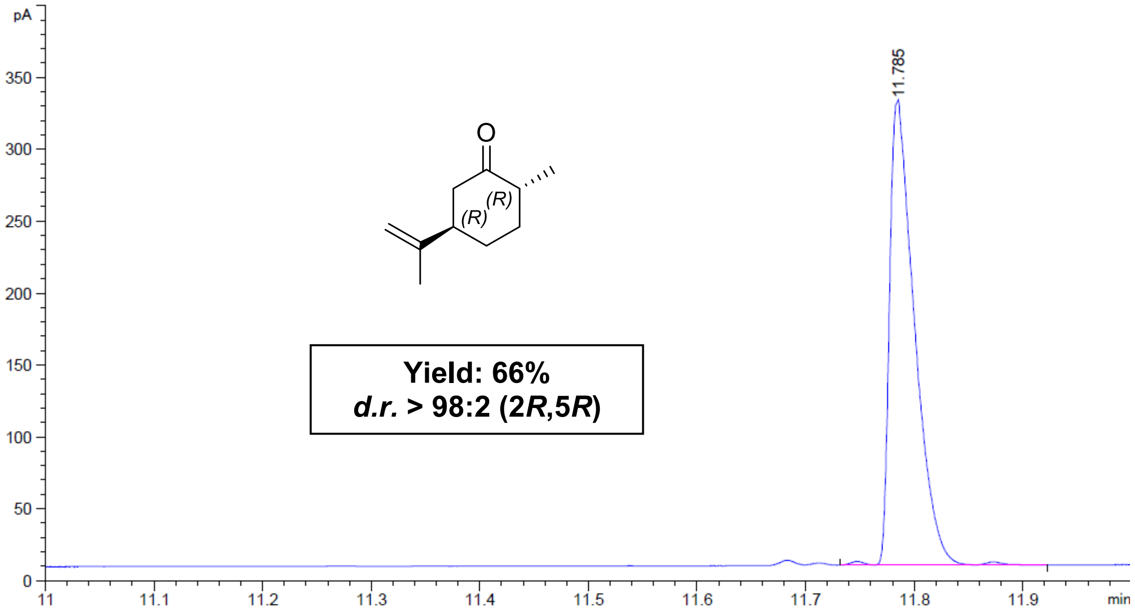

Peak #	RetTime [min]	Type	Width [min]	Area [pA*s]	Height [pA]	Area %
1	11.785	VV R	0.0243	516.21198	322.02209	1.000e2
Totals :				516.21198	322.02209	

Figure S25. Transformation of carvone derivative *S*-**5** with NCR.

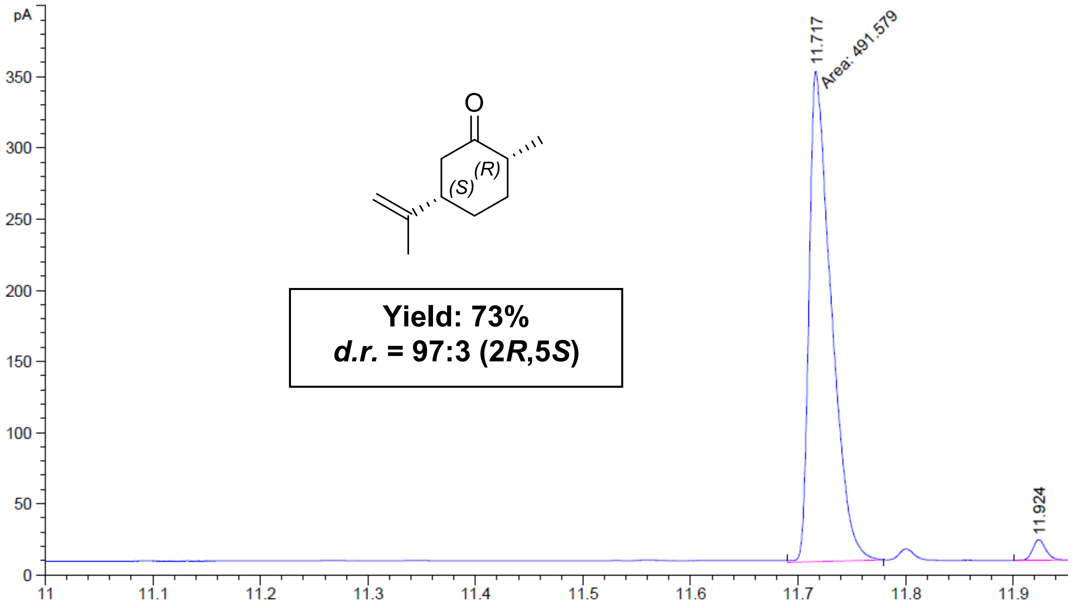

Peak #	RetTime [min]	Type	Width [min]	Area [pA*s]	Height [pA]	Area %
1	11.717	MM	0.0237	491.57895	345.78677	97.56321
2	11.924	BB	0.0141	12.27796	14.20669	2.43679
Totals :				503.85690	359.99346	

Figure S26. Transformation of carvone derivative *S*-**5** with pQR1907.

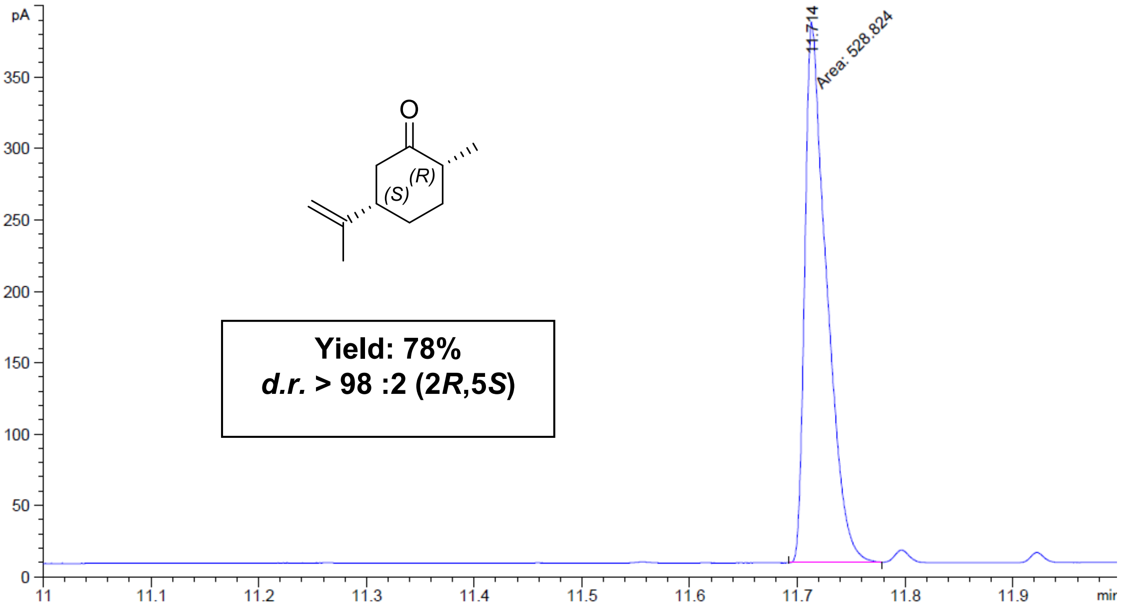

Peak #	RetTime [min]	Type	Width [min]	Area [pA*s]	Height [pA]	Area %
1	11.714	MM	0.0230	528.82355	382.68533	1.000e2
Totals :				528.82355	382.68533	

Analytical chiral HPLC (Wieland-Miescher ketone consumption)

Analyses of the reactions were performed using an Agilent 1260 Infinity HPLC with an OJ chiralcel column (250 x 4.6 mm). After injection of the sample (10 µL), elution was carried out at 0.95 mL/min with an isocratic elution mode using a mixture of hexane: *i*PrOH (90:10). The UV-detection was performed at λ = 230 nm.

Figure S27. Calibration curve for Wieland-Miescher ketone **8**.

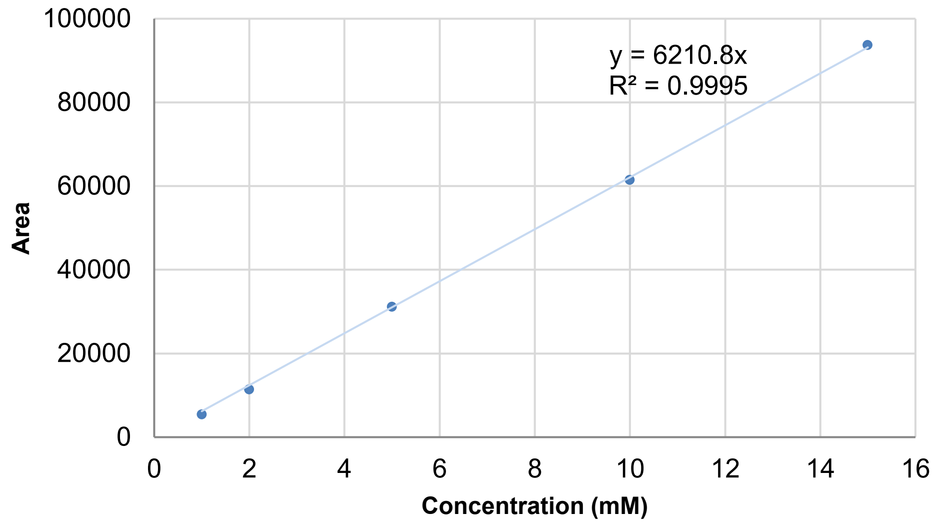

Figure S28. HPLC chromatogram - *rac*-**8**.

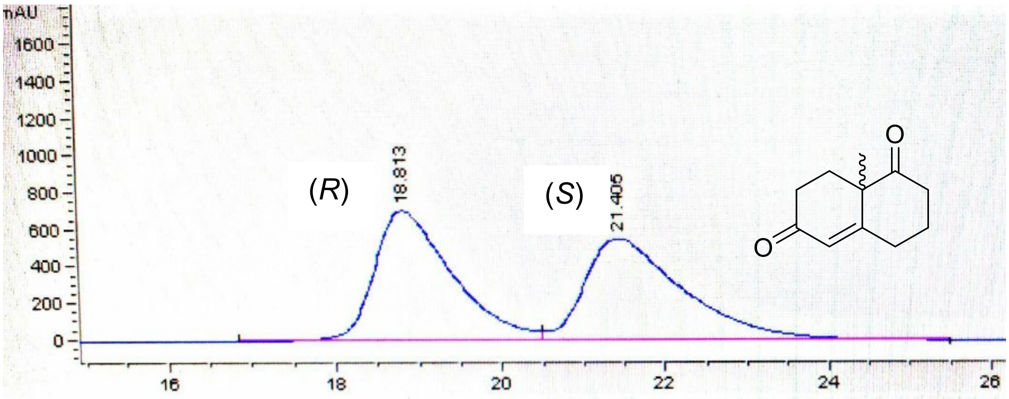

#	Time	Area	Height
1	18.813	46668	709.6
2	21.405	47070.4	563.1

Figure S29. HPLC chromatogram - *R*-**8**

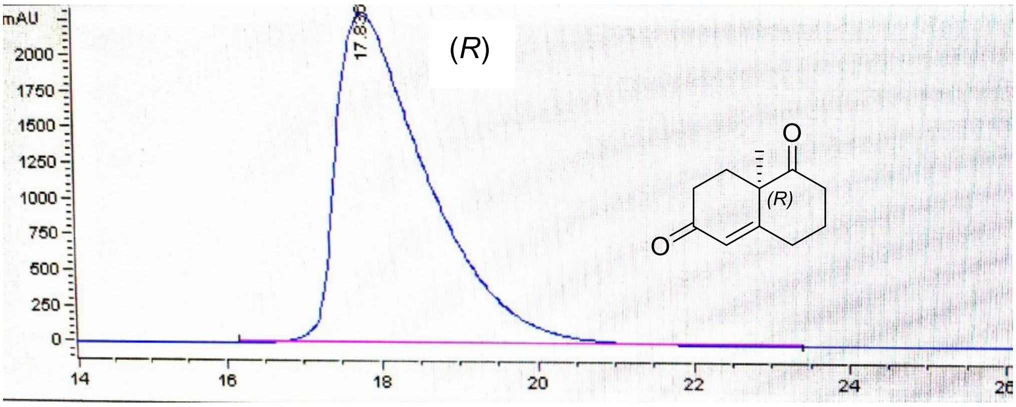

Time	Area	Height
17.836	184757.4	2270.8

Figure S30. HPLC chromatogram - reduction of *S*-**8** with pQR1907 (no starting material remaining).

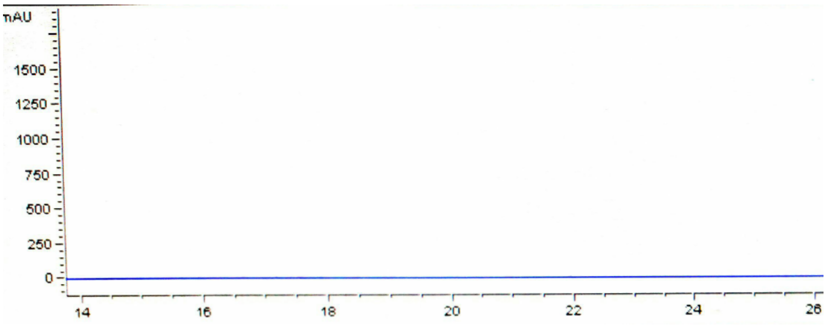

NMR spectra

Synthesis of (2*R*,5*S*)-2-methyl-5-(prop-1-en-2-yl)cyclohexan-1-one (6)

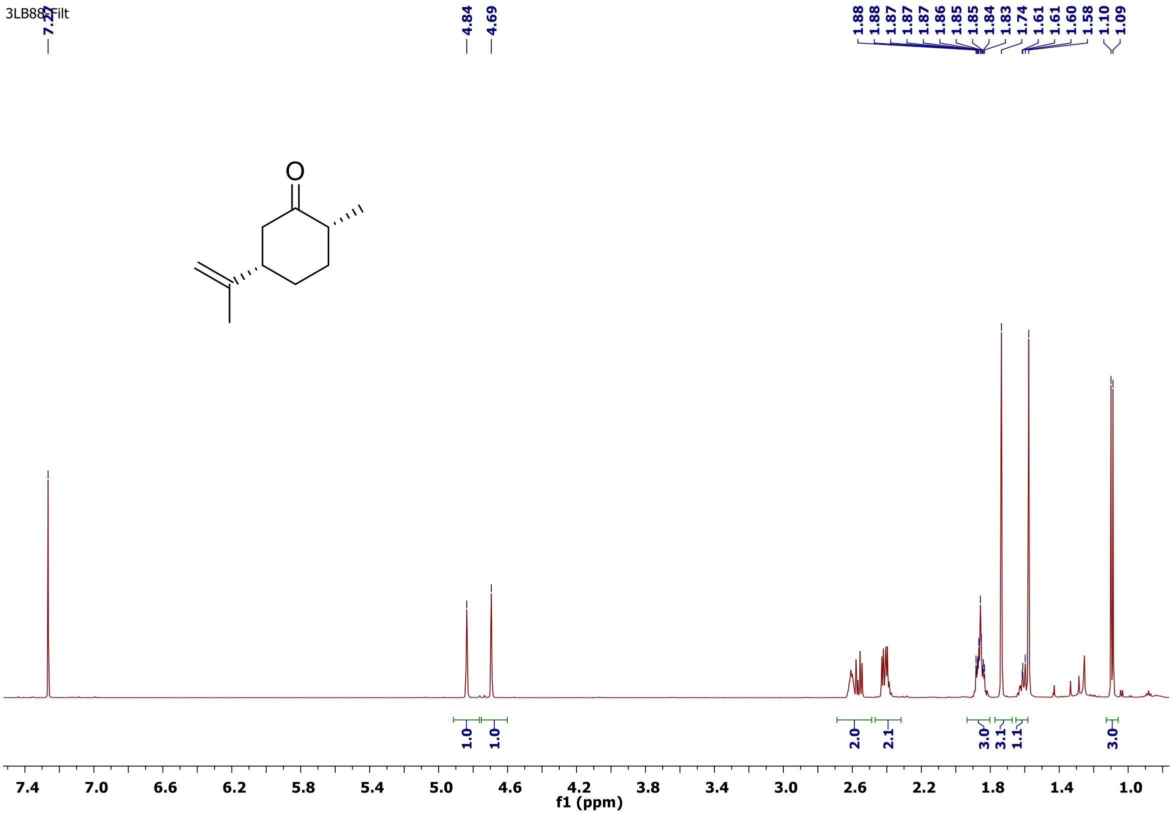

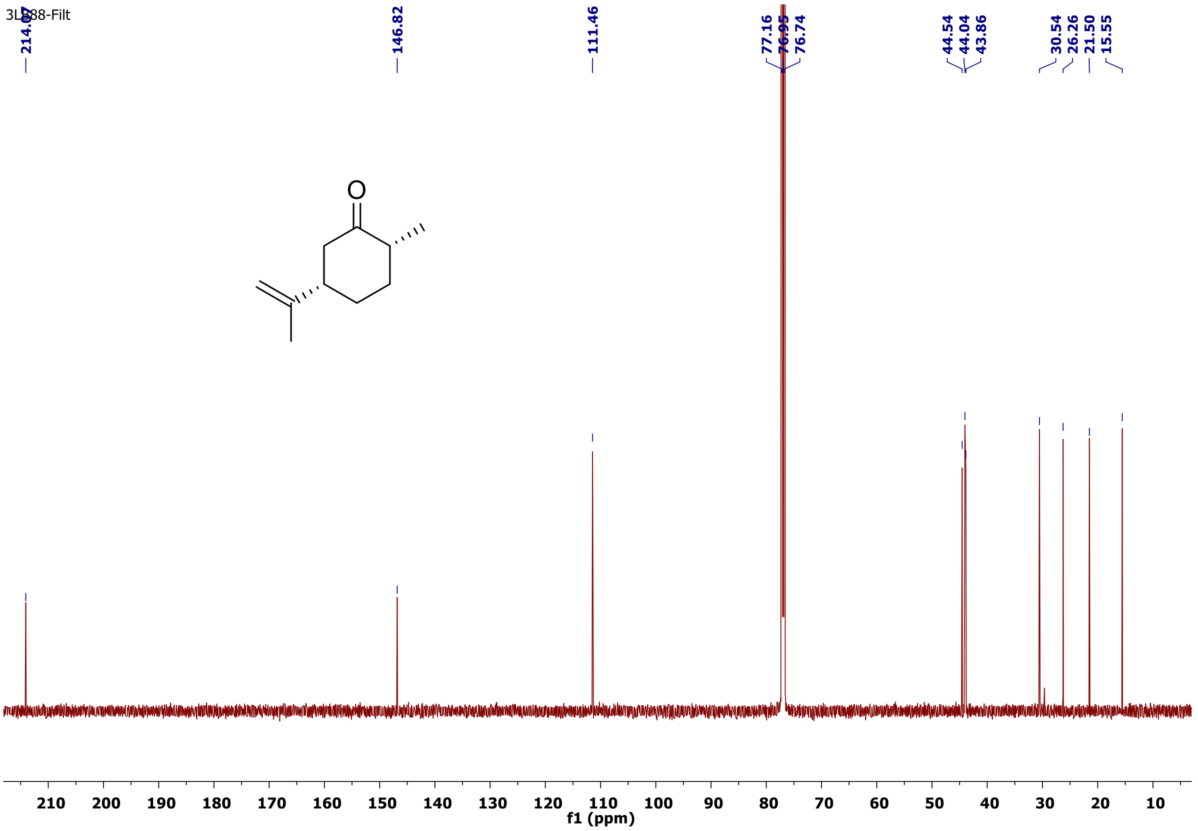

Synthesis of (8a*S*)-8a-methylhexahydronaphthalene-1,6(2*H*,5*H*)-dione (9)

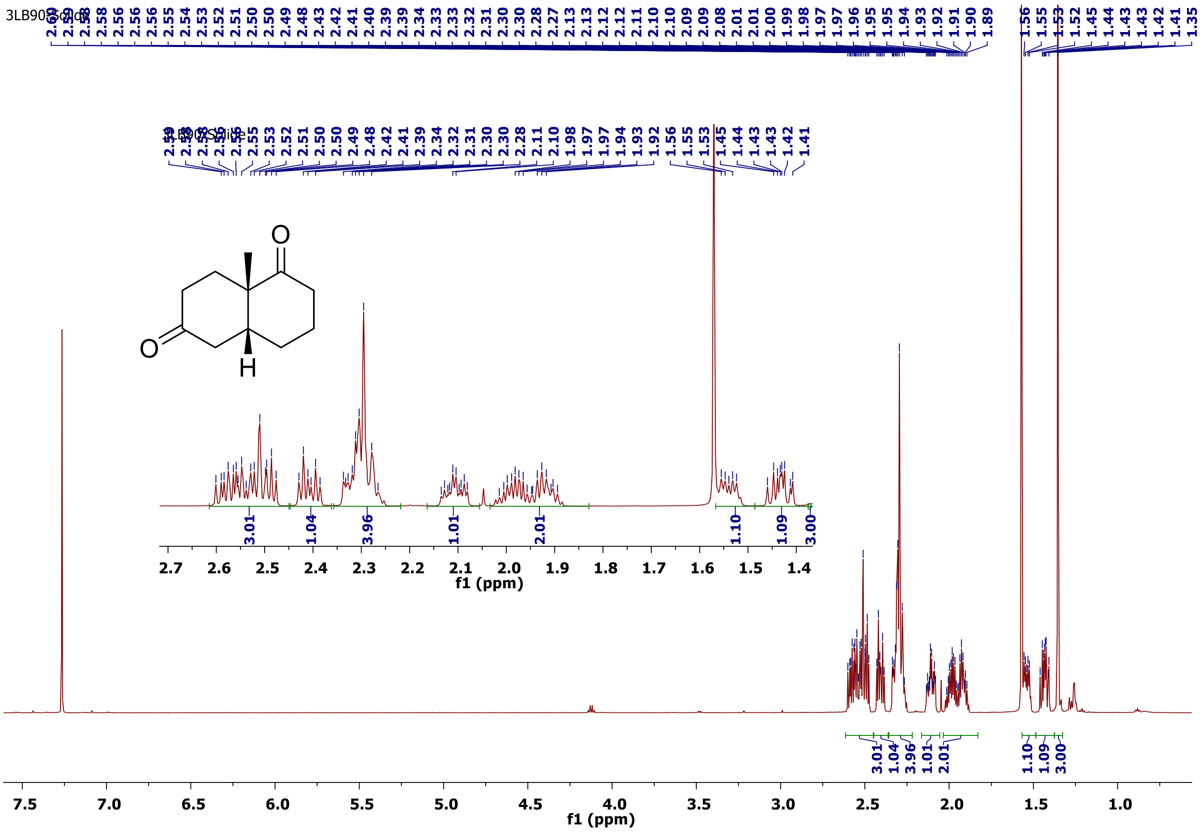

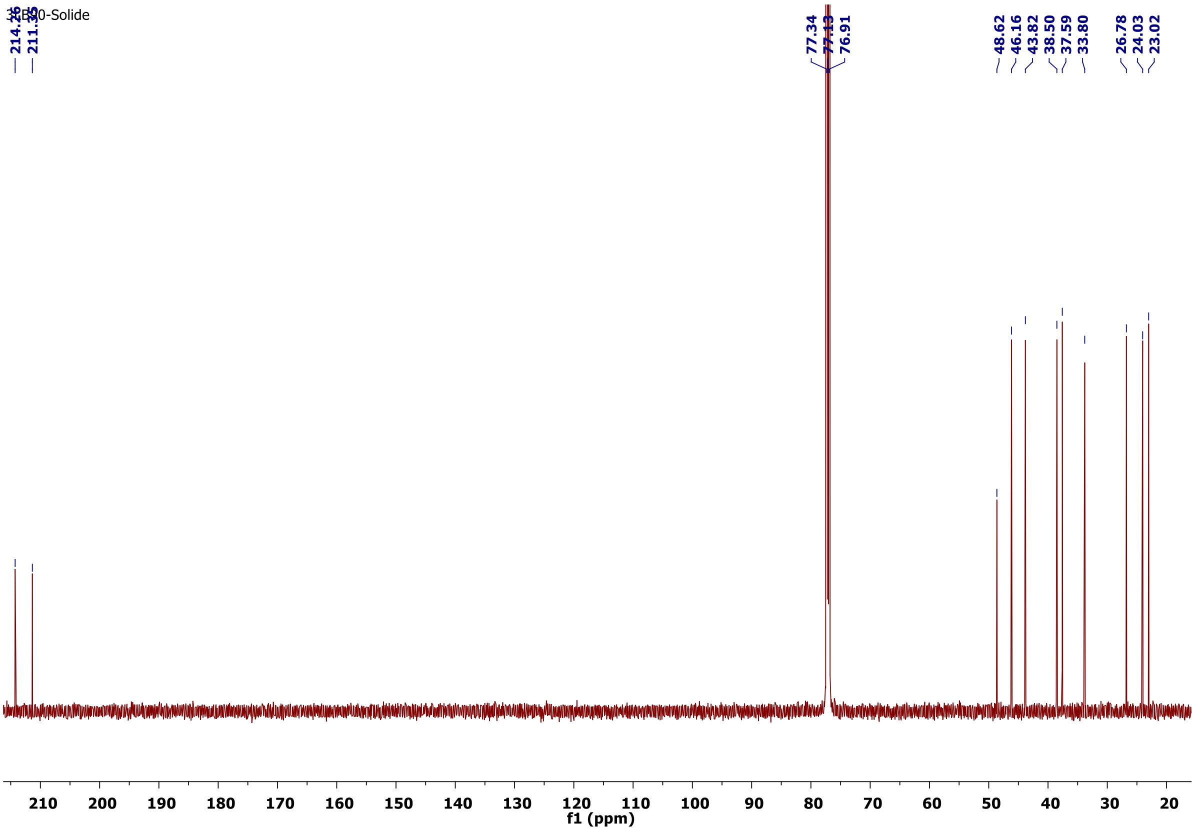

Synthesis of (4a*R*,5*R*,8a*S*)-5,8a-dimethylhexahydronaphthalene-1,6(2*H*,5*H*)-dione (12)

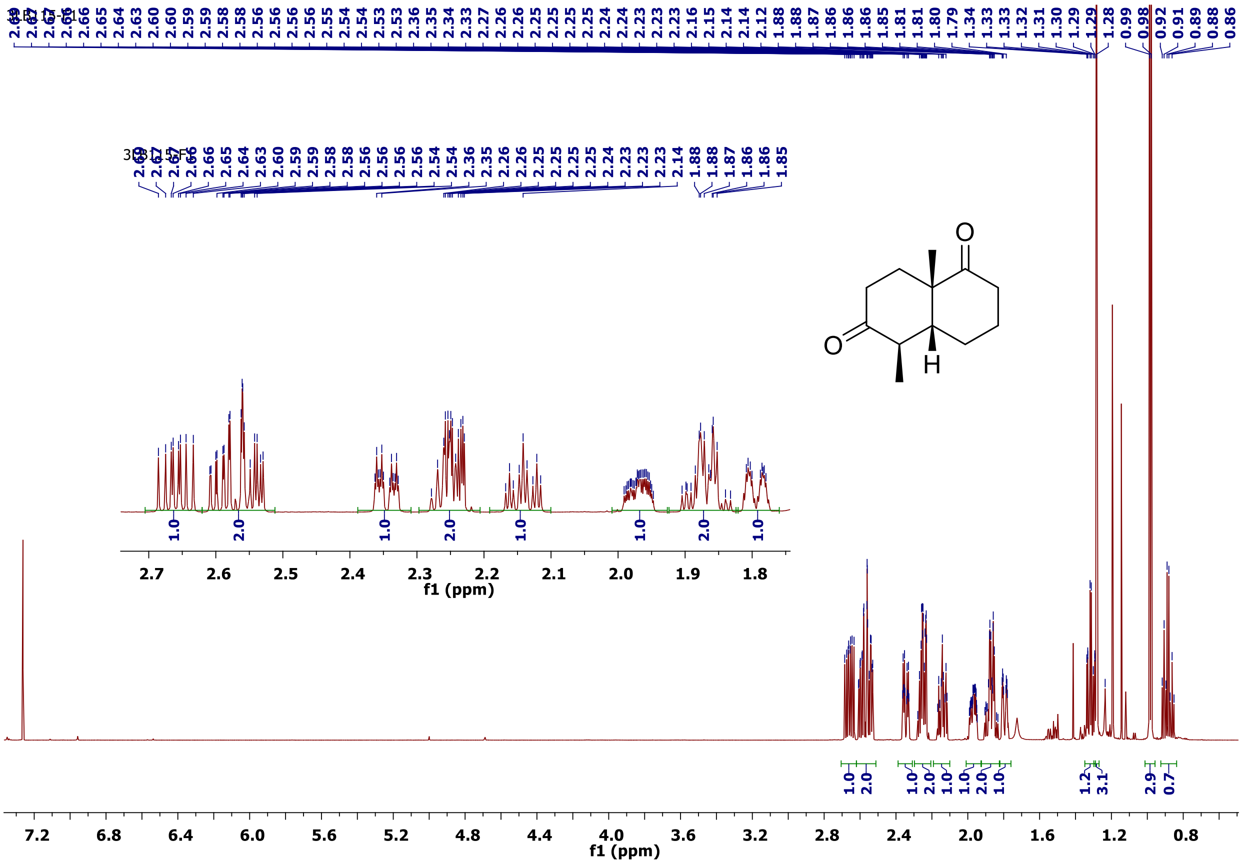

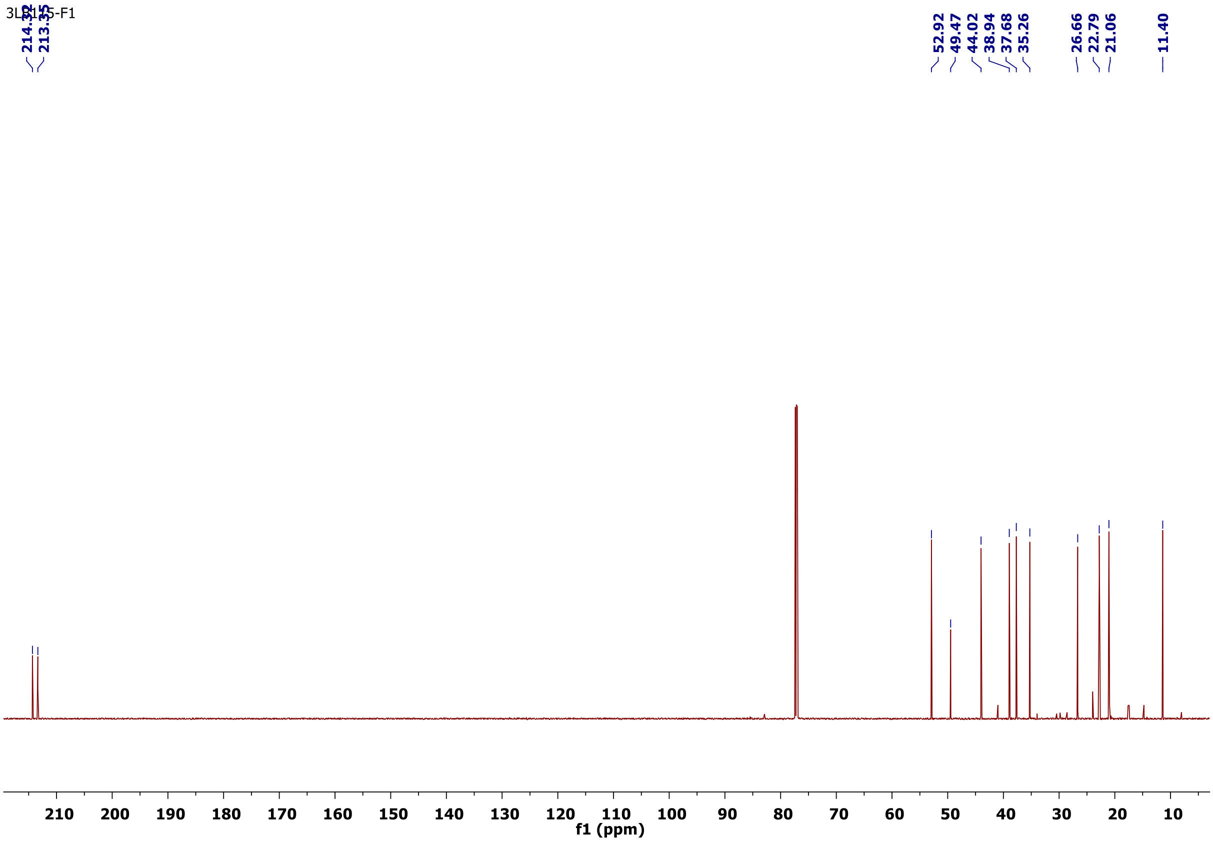

(4aR,5R,8aR)-5,8a-dimethylhexahydronaphthalene-1,6(2H,5H)-dione (13)

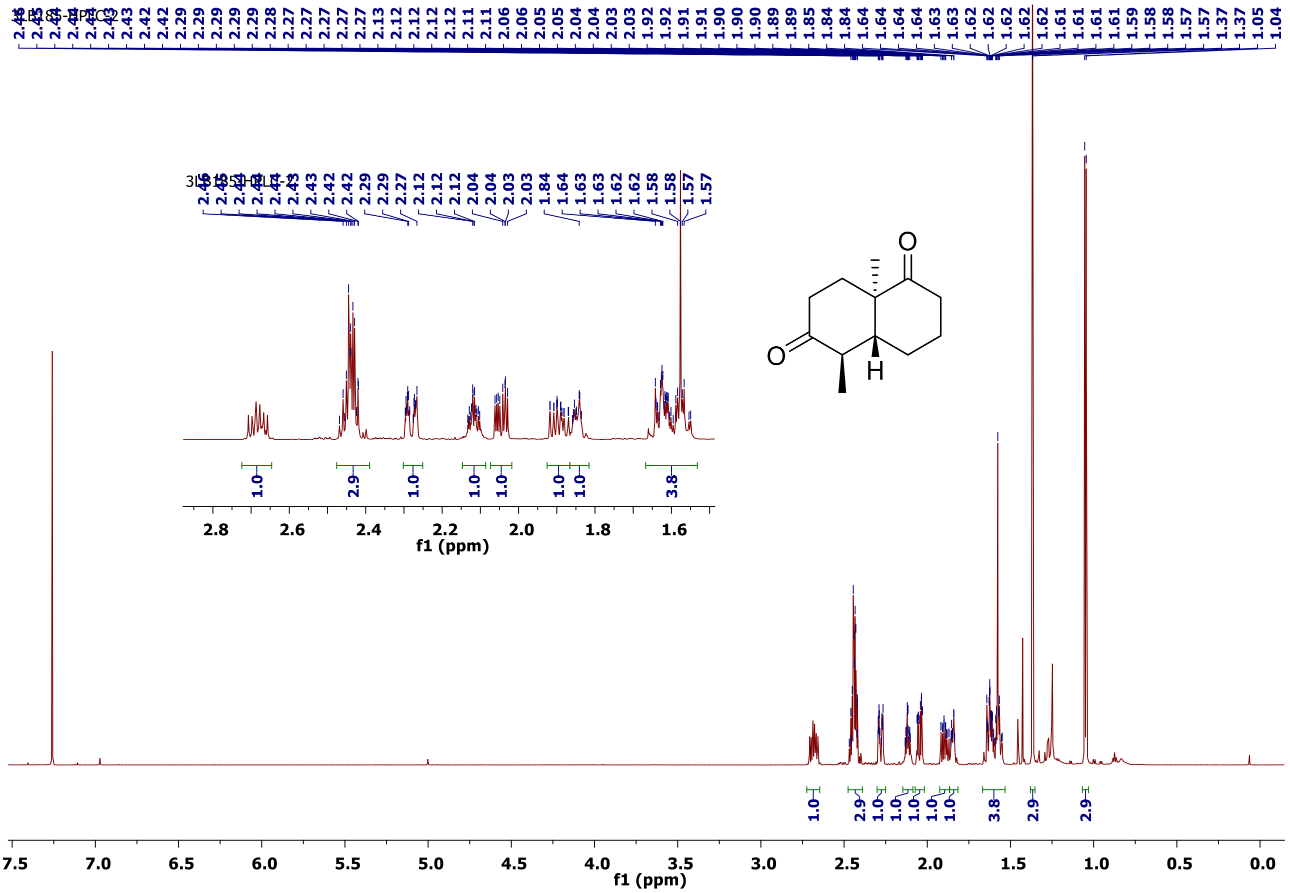

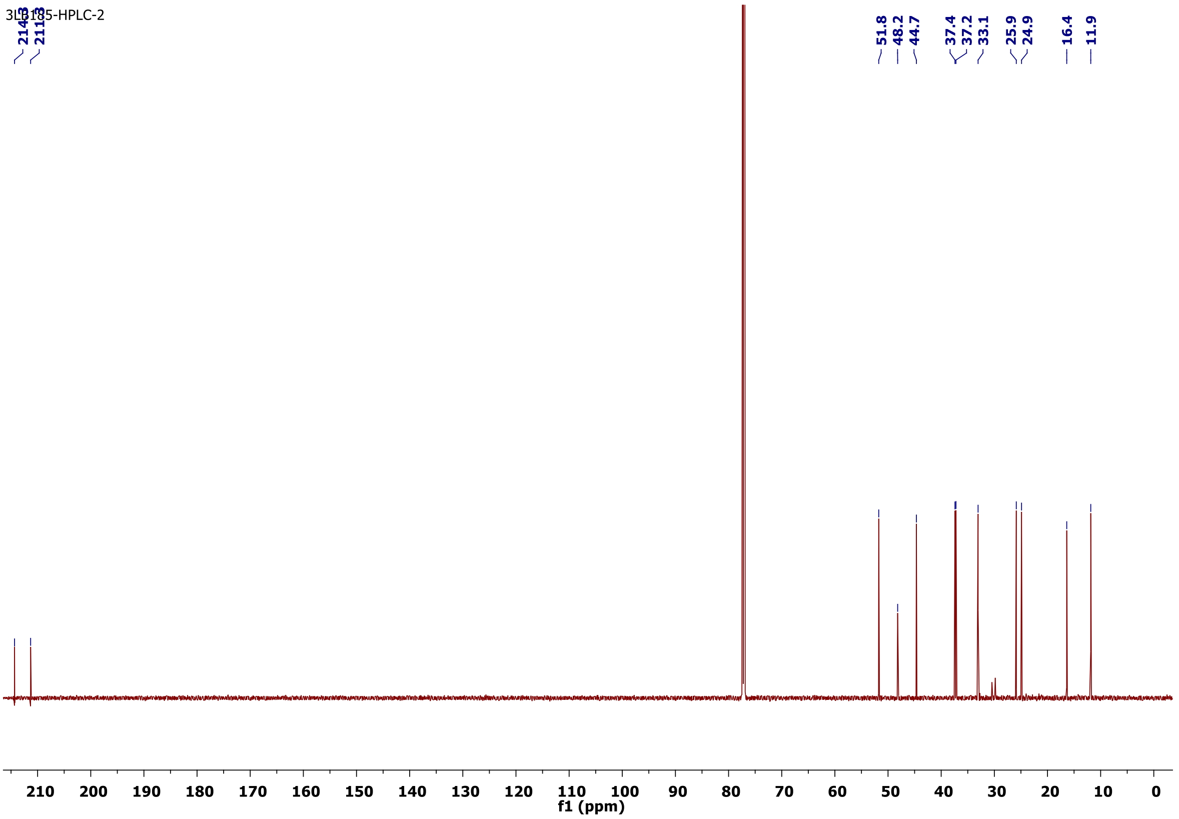

Synthesis of (3a*R*,7a*S*)-7a-methylhexahydro-1*H*-indene-1,5(4*H*)-dione (15)

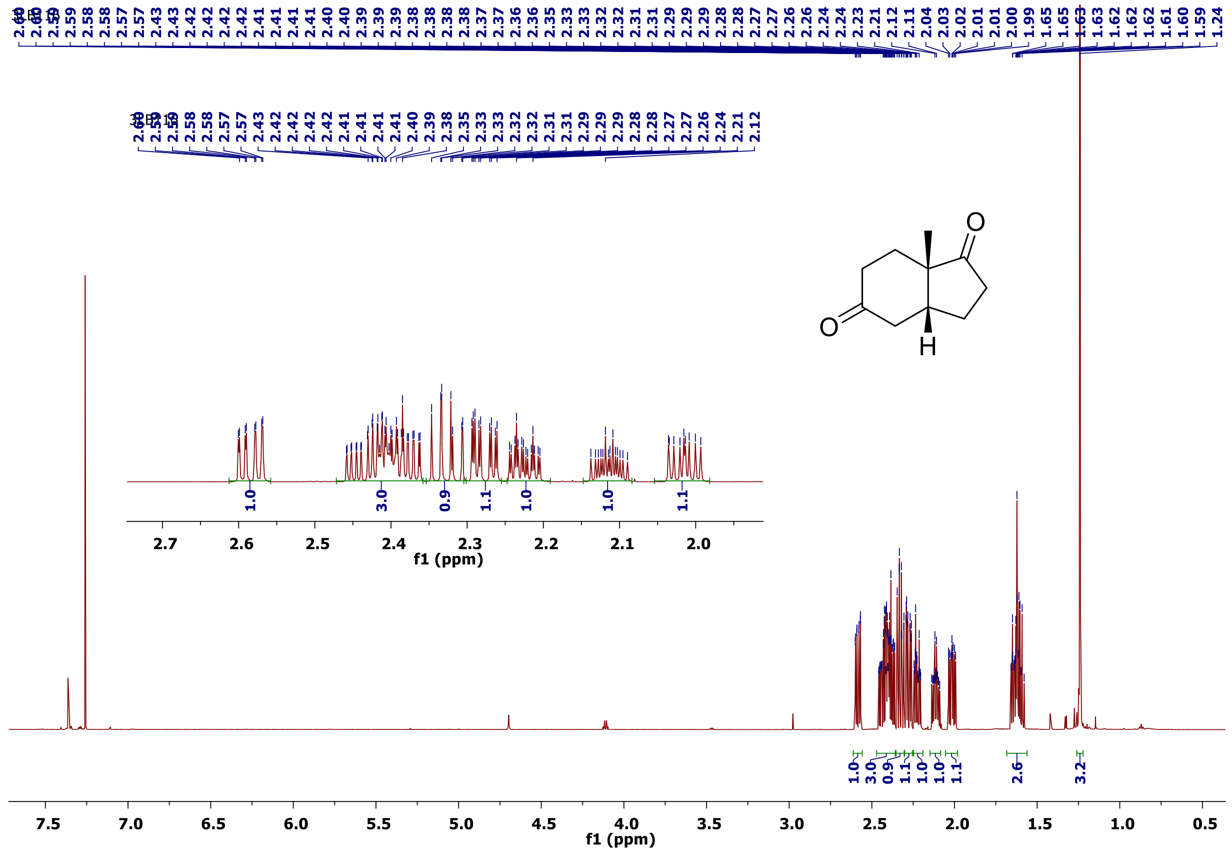

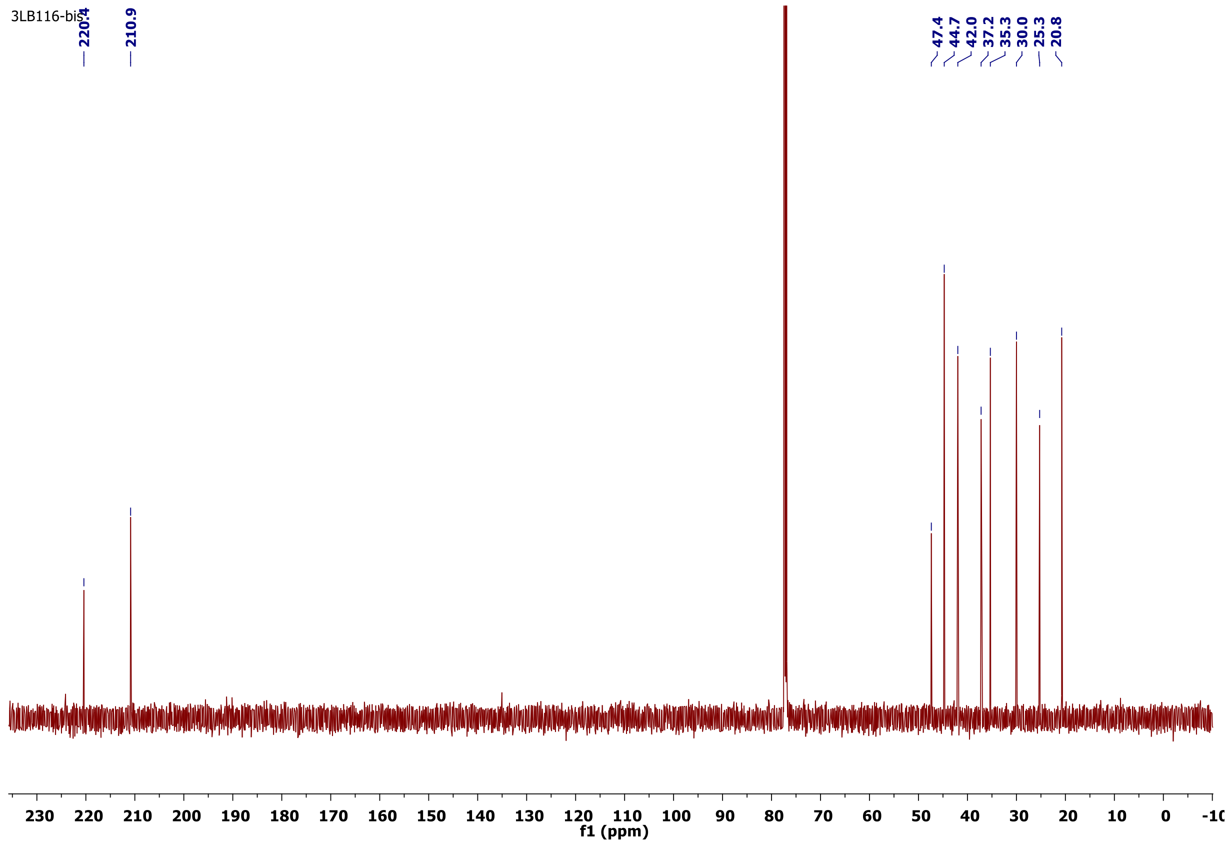

4a-methyl-3,4,4a,9,10,10a-hexahydrophenanthren-2(1H)-one (17)

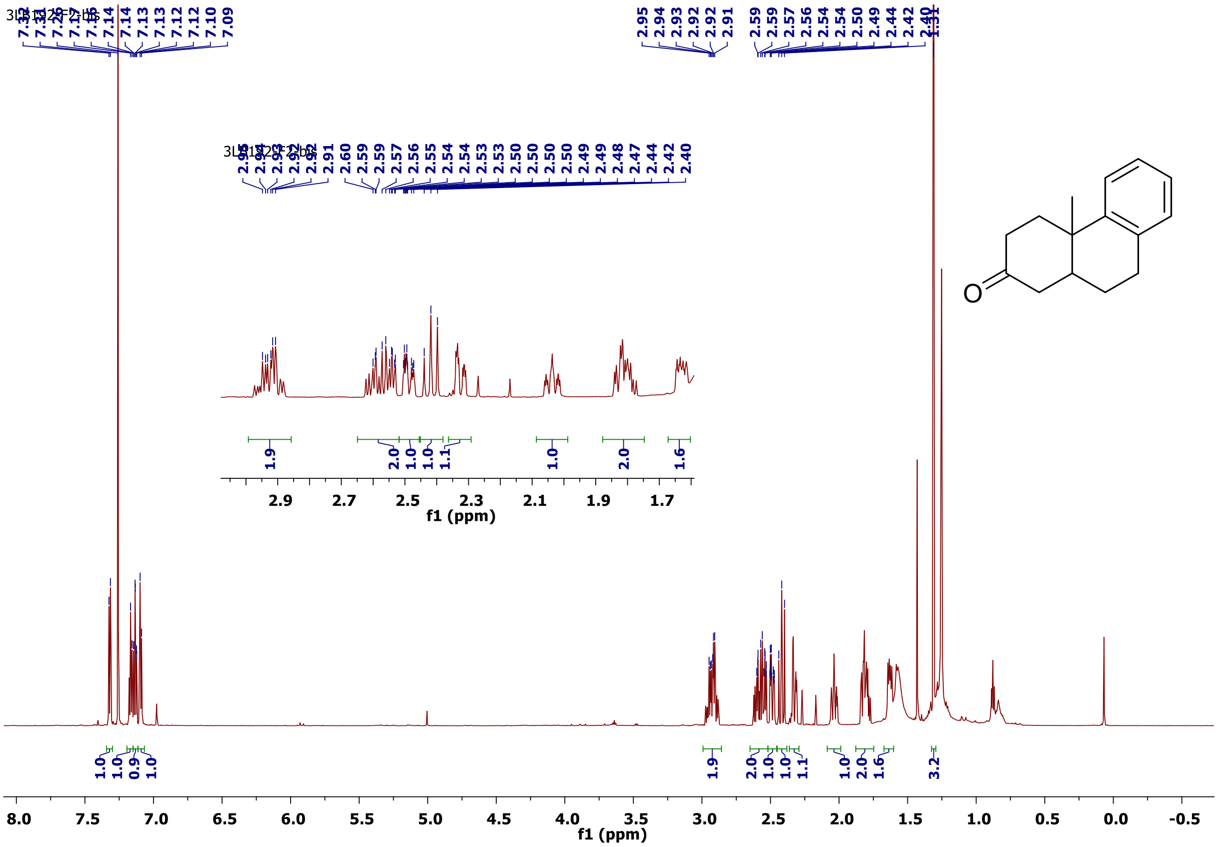

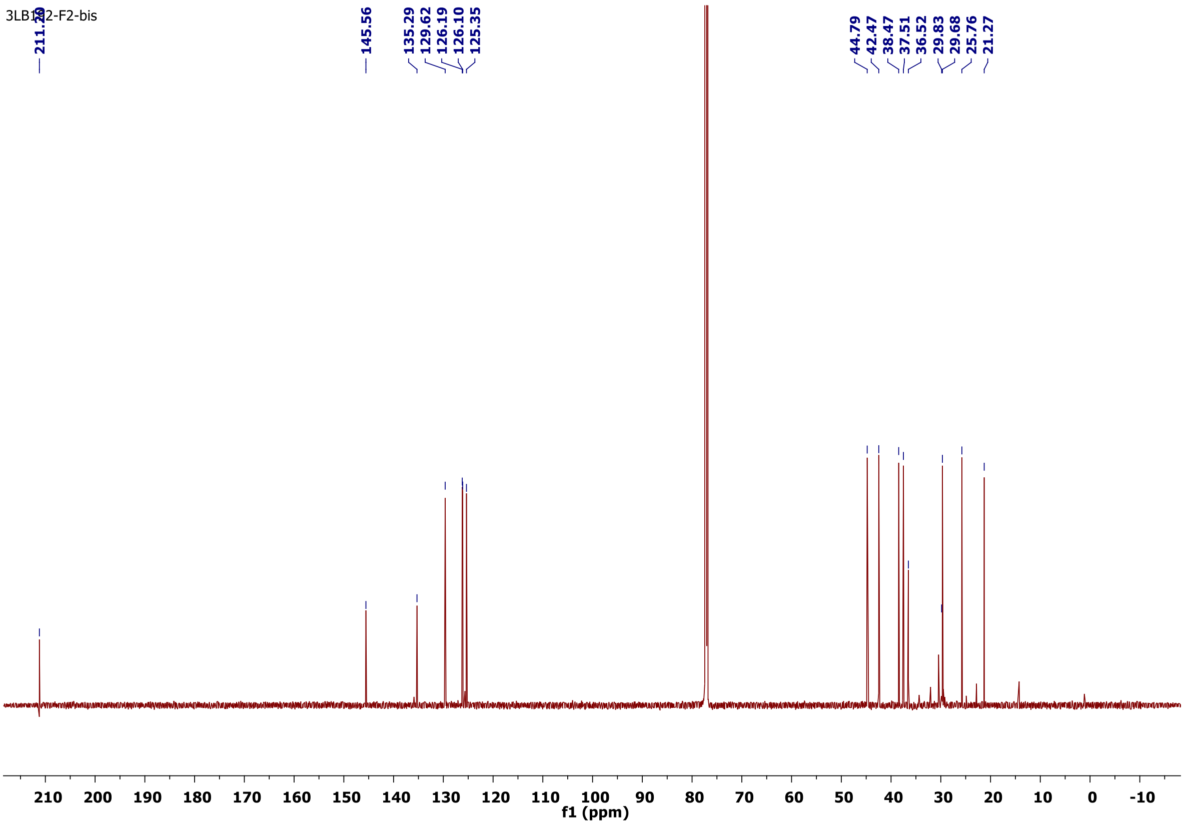

REFERENCES

(1) Peng, Y.; Leung, H. C. M.; Yiu, S. M.; Chin, F. Y. L. IDBA-UD: A de Novo Assembler for Single-Cell and Metagenomic Sequencing Data with Highly Uneven Depth. *Bioinformatics* **2012**, *28*, 1420–1428.

(2) Hyatt, D.; Chen, G.-L.; LoCascio, P. F.; Land, M. L.; Larimer, F. W.; Hauser, L. J. Prodigal: Prokaryotic Gene Recognition and Translation Initiation Site Identification. *BMC Bioinformatics* **2010**, *11*, 119.

(3) Finn, R. D.; Coggill, P.; Eberhardt, R. Y.; Eddy, S. R.; Mistry, J.; Mitchell, A. L.; Potter, S. C.; Punta, M.; Qureshi, M.; Sangrador-Vegas, A.; et al. The Pfam Protein Families Database: Towards a More Sustainable Future. *Nucleic Acids Res.* **2016**, *44*, D279–D285.

(4) Lees, J.; Yeats, C.; Redfern, O.; Clegg, A.; Orengo, C. Gene3D: Merging Structure and Function for a Thousand Genomes. *Nucleic Acids Res.* **2009**, *38*, 296–300.

(5) Müller, A.; Hauer, B.; Rosche, B. Asymmetric Alkene Reduction by Yeast Old Yellow Enzymes and by a Novel Zymomonas Mobilis Reductase. *Biotechnol. Bioeng.* **2007**, *98*, 22–29.

(6) McWilliam, H.; Li, W.; Uludag, M.; Squizzato, S.; Park, Y. M.; Buso, N.; Cowley, A. P.; Lopez, R. Analysis Tool Web Services from the EMBL-EBI. *Nucleic Acids Res.* **2013**, *41*, 597–600.

(7) Kumar, S.; Stecher, G.; Tamura, K. MEGA7: Molecular Evolutionary Genetics Analysis Version 7.0 for Bigger Datasets. *Mol. Biol. Evol.* **2016**, *33*, 1870–1874.

(8) Letunic, I.; Bork, P. Interactive Tree Of Life (ITOL) v4: Recent Updates and New Developments. *Nucleic Acids Res.* **2019**, *47*, W256–W259.

(9) Scholtissek, A.; Tischler, D.; Westphal, A.; van Berkel, W.; Paul, C. Old Yellow Enzyme-Catalysed Asymmetric Hydrogenation: Linking Family Roots with Improved Catalysis. *Catalysts* **2017**, *7*, 130.

(10) Peters, C.; Frasson, D.; Sievers, M.; Buller, R. Novel Old Yellow Enzyme Subclasses. *ChemBioChem* **2019**, *20*, 1569–1577.

(11) Kelley, L. A.; Mezulis, S.; Yates, C. M.; Wass, M. N.; Sternberg, M. J. E. The Phyre2 Web Portal for Protein Modeling, Prediction and Analysis. *Nat. Protoc.* **2015**, *10*, 845.

(12) Benkert, P.; Biasini, M.; Schwede, T. Toward the Estimation of the Absolute Quality of Individual Protein Structure Models. *Bioinformatics* **2011**, *27*, 343–350.

(13) Waterhouse, A.; Bertoni, M.; Bienert, S.; Studer, G.; Tauriello, G.; Gumienny, R.; Heer, F. T.; de Beer, T. A. P.; Rempfer, C.; Bordoli, L.; et al. SWISS-MODEL: Homology Modelling of Protein Structures and Complexes. *Nucleic Acids Res.* **2018**, *46*, W296–W303.

(14) Studer, G.; Biasini, M.; Schwede, T. Assessing the Local Structural Quality of Transmembrane Protein Models Using Statistical Potentials (QMEANBrane). *Bioinformatics* **2014**, *30*, i505–i511.

(15) Trott, O.; Olson, A., J. Software News and Update AutoDock Vina: Improving the Speed and Accuracy of Docking with a New Scoring Function, Efficient Optimization, and Multithreading. *J. Comput. Chem.* **2010**, *31*, 455–461.

(16) Iqbal, N.; Rudroff, F.; Brigé, A.; Van Beeumen, J.; Mihovilovic, M. D. Asymmetric Bioreduction of Activated Carbon-Carbon Double Bonds Using Shewanella Yellow Enzyme (SYE-4) as Novel Enoate Reductase. *Tetrahedron* **2012**, *68*, 7619–7623.

(17) Ramachary, D. B.; Sakthidevi, R. Combining Multi-Catalysis and Multi-Component Systems for the Development of One-Pot Asymmetric Reactions: Stereoselective Synthesis of Highly Functionalized Bicyclo[4.4.0]Decane-1,6-Diones. *Org. Biomol. Chem.* **2008**, *6*, 2488–2492.

(18) Stephenson, D. S.; Binsch, G. Automated Analysis of High-Resolution NMR Spectra. I. Principles and Computational Strategy. *J. Magn. Reson.* **1980**, *37*, 395–407.

(19) Aliev, A. E.; Courtier-Murias, D. Conformational Analysis of L-Prolines in Water. *J. Phys. Chem. B* **2007**, *111*, 14034–14042.

(20) Haasnoot, C. A. G.; de Leeuw, F. A. A. M.; Altona, C. The Relationship between Proton-Proton NMR Coupling Constants and Substituent Electronegativities—I: An Empirical Generalization of the Karplus Equation. *Tetrahedron* **1980**, *36*, 2783–2792.

(21) Haasnoot, C. A. G.; de Leeuw, F. A. A. M.; Altona, C. The Relationship between Proton-Proton NMR Coupling Constants and Substituent Electronegativities—I: An Empirical Generalization of the Karplus Equation. *Tetrahedron* **1980**, *36*, 2783–2792.

(22) Hall, M.; Stueckler, C.; Hauer, B.; Stuermer, R.; Friedrich, T.; Breuer, M.; Kroutil, W.; Faber, K. Asymmetric Bioreduction of Activated C=C Bonds Using Zymomonas Mobilis NCR Enoate Reductase and Old Yellow Enzymes OYE 1-3 from Yeasts. *European J. Org. Chem.* **2008**, No. 9, 1511–1516.

(23) Reich, S.; Hoeffken, H. W.; Rosche, B.; Nestl, B. M.; Hauer, B. Crystal Structure Determination and Mutagenesis Analysis of the Ene Reductase NCR. *ChemBioChem* **2012**, *13*, 2400–2407.

(24) Scholtissek, A.; Tischler, D.; Westphal, H. A.; van Berkel, J. W.; Paul, E. C. Old Yellow Enzyme-Catalysed Asymmetric Hydrogenation: Linking Family Roots with Improved Catalysis. *Catalysts*. 2017, pp 130–154.

(25) Nett, N.; Duewel, S.; Richter, A. A.; Hoebenreich, S. Revealing Additional Stereocomplementary Pairs of Old Yellow Enzymes by Rational Transfer of Engineered Residues. *ChemBioChem* **2017**, *18*, 685–691.
